# Pregnancy-specific malarial immunity and risk of malaria in pregnancy and adverse birth outcomes: a systematic review

**DOI:** 10.1186/s12916-019-1467-6

**Published:** 2020-01-16

**Authors:** Julia C. Cutts, Paul A. Agius, Zaw Lin, Rosanna Powell, Kerryn Moore, Bridget Draper, Julie A. Simpson, Freya J. I. Fowkes

**Affiliations:** 1 Burnet Institute, 85 Commercial Road, Melbourne, Victoria 3004 Australia; 2 Department of Epidemiology and Preventive Medicine, Monash University, Melbourne, Australia; 3 Centre for Epidemiology and Biostatistics, Melbourne School of Population and Global Health, The University of Melbourne, Melbourne, Australia; 4 Department of Infectious Diseases, Monash University, Melbourne, Australia

**Keywords:** Malaria, *Plasmodium falciparum*, Pregnancy, Immunity, Antibodies, Epidemiology, Systematic review, Meta-analysis

## Abstract

**Background:**

In endemic areas, pregnant women are highly susceptible to *Plasmodium falciparum* malaria characterized by the accumulation of parasitized red blood cells (pRBC) in the placenta. In subsequent pregnancies, women develop protective immunity to pregnancy-associated malaria and this has been hypothesized to be due to the acquisition of antibodies to the parasite variant surface antigen VAR2CSA. In this systematic review we provide the first synthesis of the association between antibodies to pregnancy-specific *P. falciparum* antigens and pregnancy and birth outcomes.

**Methods:**

We conducted a systematic review and meta-analysis of population-based studies (published up to 07 June 2019) of pregnant women living in *P. falciparum* endemic areas that examined antibody responses to pregnancy-specific *P. falciparum* antigens and outcomes including placental malaria, low birthweight, preterm birth, peripheral parasitaemia, maternal anaemia, and severe malaria.

**Results:**

We searched 6 databases and identified 33 studies (30 from Africa) that met predetermined inclusion and quality criteria: 16 studies contributed estimates in a format enabling inclusion in meta-analysis and 17 were included in narrative form only. Estimates were mostly from cross-sectional data (10 studies) and were heterogeneous in terms of magnitude and direction of effect. Included studies varied in terms of antigens tested, methodology used to measure antibody responses, and epidemiological setting. Antibody responses to pregnancy-specific pRBC and VAR2CSA antigens, measured at delivery, were associated with placental malaria (9 studies) and may therefore represent markers of infection, rather than correlates of protection. Antibody responses to pregnancy-specific pRBC, but not recombinant VAR2CSA antigens, were associated with trends towards protection from low birthweight (5 studies).

**Conclusions:**

Whilst antibody responses to several antigens were positively associated with the presence of placental and peripheral infections, this review did not identify evidence that any specific antibody response is associated with protection from pregnancy-associated malaria across multiple populations. Further prospective cohort studies using standardized laboratory methods to examine responses to a broad range of antigens in different epidemiological settings and throughout the gestational period, will be necessary to identify and prioritize pregnancy-specific *P. falciparum* antigens to advance the development of vaccines and serosurveillance tools targeting pregnant women.

## Background

In malaria-endemic areas, individuals can acquire clinical immunity to *Plasmodium falciparum* malaria after repeated exposure and symptomatic episodes in adults are relatively rare [1]. Despite this acquired immunity, pregnant women are highly susceptible to *P. falciparum* malaria. Pregnancy-associated malaria (PAM) represents a major public health problem, leading to poor outcomes for both mother and infant, including maternal mortality, maternal anaemia, miscarriage, stillbirth, low birthweight, and preterm birth [2–7]. In endemic regions, primigravidae are at greatest risk of PAM, and the frequency and density of both placental and peripheral *P. falciparum* infection decreases with successive pregnancies [3, 8–12].

Malaria in pregnancy is characterized by the accumulation of *P. falciparum* parasitized red blood cells (pRBC) in the placental intervillous space, often observed with macrophage infiltration, fibrinoid, and parasite pigment deposits [13, 14]. Parasites taken from infected placentas display preferential binding to the glycosaminoglycan chondroitin sulfate A (CSA) [15], present on the surface of placental syncytiotrophoblasts and intervillous spaces [15–17]. This binding phenotype is rarely observed in parasites taken from non-pregnant individuals [15, 18–21], which are more likely to bind to receptors CD36 and ICAM-1 in the vascular endothelium. Thus, the parasites that infect pregnant women are understood to constitute a distinct population to those that infect non-pregnant individuals. Parasite binding to CSA on syndecan-1 [22] is mediated by the *P. falciparum* erythrocyte membrane protein 1 (PfEMP1) family member VAR2CSA [23–27], expressed on the surface of pRBC. VAR2CSA is a large (350 kDa) protein with six Duffy-binding-like (DBL) domains (DBL1–6) and three interdomain (ID) regions [28–30]. The development of protective immunity to PAM over successive pregnancies has largely been assumed to be due to the acquisition of antibodies to VAR2CSA, specifically those that block adhesion to CSA. Two vaccine candidates based on the N-terminal CSA-binding region of VAR2CSA [28] have entered early-stage clinical trials: PAMVAC is comprised of domains ID1-DBL2X-ID2a from the *P. falciparum* strain FCR3 [31, 32] and PRIMVAC is comprised of domains DBL1X–DBL2X from *P.f.* 3D7 [33].

Several studies have demonstrated parity-dependent increases in antibody responses to pregnancy-specific variant surface antigens on pRBC [18, 21, 34–36], anti-adhesion antibodies to CSA-binding parasites [37, 38], and some, but not all, VAR2CSA domains [39–42], and a stronger correlation between parity and antibody responses has been observed in areas of more intense transmission [43]. Despite strong evidence for parity-dependent acquisition of antibodies to VAR2CSA, and evidence for a role for VAR2CSA in mediating adhesion to CSA in the placenta, direct epidemiological evidence for a protective effect of VAR2CSA antibodies in preventing PAM and associated adverse pregnancy and birth outcomes has been inconsistent. Furthermore, the specific antigenic targets, and functional responses necessary for protection against malaria in pregnancy and poor birth outcomes has not been established across multiple populations. We conducted a systematic review and meta-analysis of population-based studies examining associations between antibodies to pregnancy-specific *P. falciparum* antigens, and pregnancy and birth outcomes including placental malaria, low birthweight, preterm birth, maternal anaemia, and severe malaria. A more comprehensive understanding of the acquired immune response to PAM will inform vaccine development and may help to identify serological correlates of immunity that could be employed in serosurveillance tools.

## Methods

### Review protocol

The Meta-analysis of Observational Studies in Epidemiology (MOOSE) working group [44] guidelines and the Preferred Reporting Items for Systematic Reviews and Meta-Analyses (PRISMA) specifications were adhered to in the conducting and reporting of this systematic review and meta-analysis [45]. A completed PRISMA checklist is included in Additional file 1.

### Search methods for identification of studies

PubMed, Web of Science, Scopus, African Index Medicus, LILACS (Latin American and Caribbean Health Sciences Literature), and the Malaria in Pregnancy Consortium databases were searched for studies published in all years up to and including 07 June 2019 that examined the association of antibody responses to pregnancy-specific *P. falciparum* antigens and pregnancy and birth outcomes. Key words included VAR2CSA, falciparum, pregnancy, parasitaemia, IgG, DBL, placental infection, antibody, immunity, protection, VSA, variant surface antigen, PfEMP1, birth outcome, birthweight, gestational age, preterm birth, and intrauterine growth restriction. Google Scholar was used to identify additional studies by senior authors of some studies identified through other database searches but was not used in a systematic manner due to the unwieldy number of records returned using key words listed above. The reference lists of obtained papers were searched for further studies. Studies reported in languages other than English were included and translated into English using online translation applications. We did not formally attempt to identify unpublished population studies because it would require us to provide substantial descriptions of the study design, sample testing and analysis used in the studies, and a review of ethical and other issues. The full search strategy for one database (PubMed) is provided (Additional file 2).

### Criteria for considering studies for this review

#### Study designs and study participants

Population-based cross-sectional, case-control, cohort studies, including treatment to reinfection studies, and randomized controlled trials (RCTs), excluding vaccine efficacy trials, were included. The primary criterion for study inclusion was pregnant women living in areas endemic for *P. falciparum* infection. All geographical locations were included. Studies which included multiple population subsets were assessed on a sub-population basis to determine eligibility for inclusion.

#### Antibody measures

We included studies that measured immunoglobulin G (IgG) responses to placental isolates, pregnancy-specific parasite strains including CS2, and other strains that had been selected for binding to chondroitin sulfate/chondroitin sulfate proteoglycans, and recombinant or synthetic defined pregnancy-specific variant surface antigens. Studies that employed the following types of assays to measure total antibody responses were considered: enzyme-linked immunosorbent assay (ELISA), multiplex assay, and flow cytometry. We also included studies that measured functional antibody responses to pregnancy-specific pRBC, including CSA binding inhibition (anti-adhesion assay), pRBC cell-agglutination, and phagocytosis. Studies in which antibodies were measured in peripheral blood taken during pregnancy and/or the immediate postpartum period were considered. For cohort studies and RCTs, if antibody responses were measured at more than two time points, results from enrolment and the latest (e.g. delivery) sampling time were extracted. Estimates from cohort studies and RCTs in which antibody responses were determined after the outcome measures of interest were excluded.

#### Maternal and birth outcomes

Outcome data were measured during pregnancy, at birth, or during the immediate postpartum period (within 72 h of delivery). If not presented in the requisite format in the original papers, authors were asked to provide data for maternal *P. falciparum* placental malaria, maternal peripheral *P. falciparum* infection, low birth weight (< 2500 g), premature birth (delivery before 37 weeks of gestation), and anaemia or severe anaemia (as defined in each study), and severe malaria, where relevant. We included estimates where women with active or active-chronic placental infection were compared to women with no placental infection, but we did not include past placental infection (often characterized by the presence of haemozoin in fibrin) as an outcome because in such cases the temporal relationship between antibody responses and infection would be difficult to ascertain, nor did we include estimates where active placental infection was compared with past infection.

#### Quality criteria

The minimum quality criteria for inclusion of studies were as follows: for placental malaria, confirmation of *P. falciparum* placental infection by slide microscopy of placental blood, polymerase chain reaction (PCR), or placental histology for the examination of *P. falciparum* parasites; for peripheral parasitaemia, detection by slide microscopy or PCR; for low birth weight, defined as less than 2500 g and birth weight was measured within 72 h of birth; and for preterm birth, defined as delivery at less than 37 weeks gestation, where gestational age must have been confirmed using Crown Rump Length (CRL) from Ultrasound and Robinson’s chart or date of last menstrual period (LMP). Studies that used rapid diagnostic tests as the sole method of diagnosis for *P. falciparum* infection were excluded. Antibody levels must have been determined in maternal peripheral blood samples preceding or at the same time as outcome measurement. Studies in which antibodies were measured in cord blood, placental blood, or infant peripheral blood were excluded. Cut-offs for positive antibody responses by ELISA or other means must have been defined using unexposed (malaria-naïve) controls or men/children from the malaria-endemic area.

#### Selection of studies

Three authors (JCC, RP, and ZL) identified possible studies and assessed the methodological quality of included studies independently, with discrepancies resolved by discussion with a fourth review author (FJIF).

#### Approaches to include all available studies and data

For studies that analysed antibody levels as the outcome variable rather than the exposure variable, where possible, data were extracted and re-analysed with the specified maternal/birth outcome as the outcome variable. If the raw data were not presented, authors of the study were invited to re-analyse or provide data for the inclusion of their study in the systematic review. In addition, we contacted several authors whose studies did not meet the inclusion criteria but contained data that were eligible for the systematic review. Contact was established through an initial email explaining the nature of the systematic review and the information required, together with a data extraction form for authors to complete (Additional file 3). If the corresponding author did not respond within three email attempts, then no further action was taken.

#### Risk of bias

For cross-sectional, RCTs, and cohort studies, selection bias was assessed by reviewing inclusion and exclusion criteria of each study. For case-control studies, the comparability of cases and controls was assessed. An additional selection bias can occur in case-control studies when cases and/or controls are selected based on criteria relating to their exposure (i.e. antibody) status or there are differences in the reporting of exposure between cases and controls. However, this is unlikely because immunoassays were after enrolment into the study. Information bias (resulting from flaws in measuring antibody and *P. falciparum* outcome data) is unlikely because antibodies are measured using immunoassays that are standardized within each study and across outcome groups. Furthermore, the quality criterion of this review ensures accurate measurement of maternal and infant outcomes and it is unlikely that measurement of outcomes would differ according to antibody groups. Initially, two authors (JCC and RP) independently assessed bias, with discrepancies resolved by discussion with a third review author (FJIF). Risk of bias assessment was collated by JC using the Risk of Bias in Non-randomized Studies—of Interventions (ROBINS-I) tool (Additional file 4) [46]. The risk of bias assessment pertains to the association between antibody responses and pregnancy and birth outcomes derived from the study, rather than the study itself.

### Data analysis

#### Data collection

Measures of association between antibody responses and maternal and birth outcomes (odds ratios [ORs], risk ratios [RRs], incidence rate ratios [IRRs], or hazard ratios [HRs]) and corresponding 95% confidence intervals (CIs) were extracted or derived using reported data or unpublished data provided by authors. OR, RR, HR, and IRR are hereinafter denoted as RR. Data extraction was performed independently by two review authors (JCC and RP) using the data extraction form (Additional file 3). For cross-sectional and case-control studies, odds ratios (ORs) were extracted or calculated where possible. For cohort studies and RCTs, risk ratios (RR), hazard ratios (HR), and incidence rate ratios (IRR) were extracted or calculated where possible or unadjusted ORs were converted to RR [47]. If provided, cross-sectional data from RCTs and cohort studies were extracted for inclusion in cross-sectional analyses. Basic information about each study, including enrolment years, age of women, and IPTp use, was extracted from individual publications where available. *P. falciparum* endemicity was categorized as low, intermediate, or high using information in the published papers. If insufficient information was provided in the publication, we used the Malaria Atlas Project website (https://map.ox.ac.uk) to obtain estimates of the *Plasmodium falciparum* parasite rate in 2–10 year olds (globally, 2000–2017) for each study site (longitude and latitude). We then categorized the endemicity of the study sites as follows: low [<10%], intermediate [≥10% to <50%], or high [≥50%].

#### Standardization of antibody measures

Measurement of antibody levels by established assays (ELISA or flow cytometry) does not produce a common metric among studies. Antibody data classified as “responders” or “non-responders” relative to a negative control (unexposed sera) were pooled, whereas categories based upon arbitrary cut-offs (including categories of responders based on statistical rankings) were simply reported in tables, but not included in the forest plots or meta-analyses. For studies where the antibody measures were analysed as continuous exposure variables, authors were asked to re-analyse their data by collapsing the antibody data into categories. If antibody or outcome data could not be provided in categorical form, the study’s key findings on the association between antibody responses and outcomes of interest were described in Table 2 and in the text, that is, the study was included in narrative terms rather than quantitative terms. For studies in which responses to multiple allelic forms of an antigen or multiple parasite isolates or strains were analysed, estimates for the most seroprevalent antibody response were presented for that population. Data on total IgG responses or the most seroprevalent subclass response were also extracted. If antibody responses to the same antigen, in the same population-based study, were reported in several publications, results from the largest sample size were presented. Separate estimates were obtained for the RR associated with pRBC VSA, VAR2CSA: DBL1, DBL1+2, ID1-ID2, ID1-ID2a, DBL2, DBL3X, DBL3–4, DBL4, DBL5, and full-length VAR2CSA (FV2). Separate estimates were calculated for functional antibody responses. Sub-group analyses were performed on women stratified by gravidity (primigravidae and multigravidae), where this was possible.

#### Synthesis of results: meta-analysis

A meta-analysis was performed, stratified by outcome, and where relevant VAR2CSA antigen and trimester of antibody and outcome determination. Cross-sectional estimates from all study designs, except for case-control studies, were combined where possible; prospective estimates from RCTs and cohort studies were combined where possible. Where there were sufficient data, a pooled estimate for each malaria outcome was calculated using a random effects model. The standard error of the natural logarithm (ln) of the RR was calculated using the formula SE(ln RR) = (ln(upper limit of 95% CI) – ln(RR)) / 1.96. The random effects meta-analyses were weighted using the inverse of the sum of the individual study sampling variances and a between-study variance component [48]. The application of weights to individual study estimates in pooled effect estimation ensure (typically smaller) studies exhibiting higher standard error do not bias point estimates and contribute to under estimation of pooled effect confidence intervals. Heterogeneity between studies was tested with the *I*^2^ statistic [49]. If the *I*^2^ statistic was ≤ 75%, a meta-analysis based on a random effects model was conducted; when the *I*^2^ statistic was > 75% and/or the lower 95% confidence limit was between 50 and 100%, the studies were not combined [49, 50]. All analyses were performed using STATA version 15.1.

## Results

### Identification and description of included studies

**Fig. 1:**
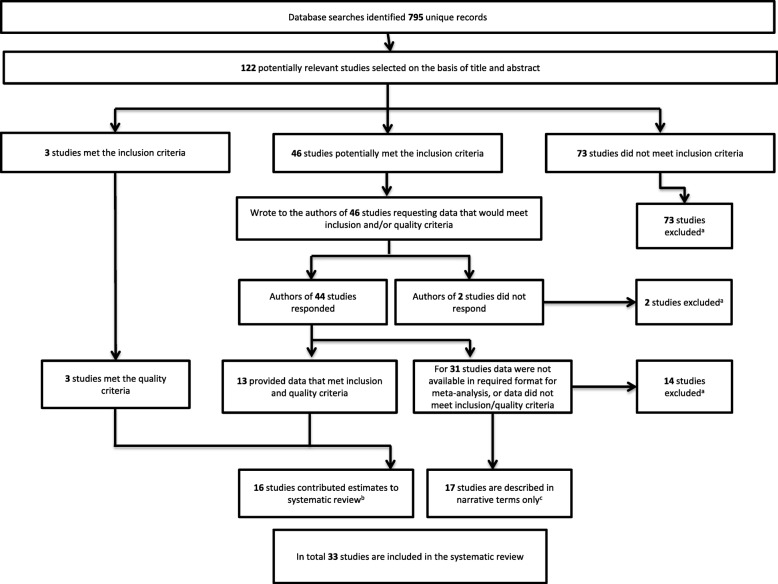
Flow chart of study identification. ^a^Excluded studies are annotated in Additional file 5. ^b^Characteristics of included studies that provided estimates are presented in Table 1. ^c^Characteristics of studies included in narrative terms are presented in Table 2

Database searches identified 795 records, from which 122 potentially relevant studies were selected based upon title and abstract. The full texts of these studies were examined to determine whether they complied with eligibility criteria: 73 did not meet the inclusion criteria; 3 fulfilled the inclusion and quality criteria; 46 studies potentially met inclusion and quality criteria and authors were contacted with responses received from authors of 44 studies (Fig. 1). Of the 44 responders, 13 authors provided data or estimates to fulfill inclusion and quality criteria, and for the remaining 31 studies, the data were not available or did not meet inclusion/quality criteria. Details of excluded studies are provided in Additional file 5. A total of 33 studies were included in the systematic review: 16 studies contributed estimates in a format enabling inclusion in meta-analysis [35, 42, 43, 51–63] (Table 1) and 17 studies are included in narrative terms only because data were not available in the required format [27, 34, 38, 39, 64–76] (Table 2). Of those 16 studies that contributed estimates, 4 were cross-sectional, 4 were cohort (two of which contributed only cross-sectional data), 6 were case-control studies, and 2 were randomized controlled trials. The included studies reported data from ten countries and the sample sizes ranged from 29 to 1377 participants. Estimates for antibody responses to the following antigens were included: pregnancy-associated pRBC, including CSA-adherent lines and isolates taken from infected placentas; full-length VAR2CSA (FV2) and VAR2CSA domains including ID1-ID2a, DBL2, DBL3, DBL3X, DBL3–4, DBL4, and DBL5. Details of the antigens included in the review are presented in Additional file 6. The most commonly examined malaria outcome was placental infection and the most common birth outcome examined was low birthweight (Tables 1 and 2). Herein, we focus on placental infection, peripheral infection, and low birthweight as key outcomes of interest. Additional forest plots and result text are included in Additional file 7 for anaemia (Additional file 7: Figures S1 and S10), severe malaria (Additional file 7: Figure S2), and preterm birth (Additional file 7: Figures S3, 11 and 12).

**Table 1:** Characteristics of the 16 included studies which contributed estimates to the systematic review

Author, year	Region, country	Enrolment period	Endemicity^a^	Study design (*n*)	Gravidity; % PG	Mean (SD) age^b^	IPTp use	Antibody responses included in review^c^	Time of antibody measurement	Clinical outcomes^d^
Aitken, 2010 [51]	Lungwena, Malawi	2003–2006	High	RCT (549)^e^	All; 23.9	24.9 (6.7)	Women randomized to standard 2 dose SP or monthly SP	pRBC: CS2	T2; T3^f^	LBW, PI, PTB, Anaemia^g^
Babakhanyan, 2014 [52]	Yaounde, Cameroon	1996–2001	Intermediate	CC (464)^h^	MG	PM+ 28 (5); PM− 30 (6)	76.3% took chemoprophylaxis	VAR2CSA: 1D1-1D2a, FV2	Delivery	PM, LBW, PI
Beeson, 2004 [53]	Blantyre, Malawi	1998–2000	Intermediate	CC (181)^i^	All; 55	PM+ 20.9; PM- 21.5	SP	pRBC: CS2 (total IgG, agglutination, CSA adhesion inhibition)	Delivery	PM
Chandrasiri, 2014 [54]	Central and southern regions, Sudan	2010	Low	CC (121)^j^	All; 33	median (IQR) 28 (24, 32)	None reported	pRBC: CS2, VAR2CSA: DBL5ε	T2/T3	SM
Cox, 2005 [55]	Brong Ahafo, Ghana	2001	High	RCT (101)^k^	PG	21.1 (2.9)	CQ at enrolment	pRBC: FCR3	T1/T2 and T2/T3	PM, PI
Duffy, 2003 [56]	Kisumu, Kenya	1995–1997	High	CS (168)	All; 28	23.5 (5.3)	None reported	pRBC: Kenyan placental isolate (CSA adhesion inhibition)	Delivery	PM, LBW, PTB, anaemia^g^
Fowkes, 2012 [57]	Maela, Thailand	1998–2000	Low	nCC (467)^l^	All; Case 22.1, control 15.7	Cases: median (IQR) 24.5 (20–30.5); Controls: 26 (21–31*)*	Pregnant women were randomized to CQ or placebo for *P. vivax* chemoprophylaxis	VAR2CSA DBL5ε	T1	PI
Fried, 2018 [42]	Ouelessebougou, Mali	2010–2013	Intermediate	cohort (657)	All; 25.8	24.1 (6.4)	SP	VAR2CSA: ID1-ID2a, DBL2, DBL3, DBL3–4, DBL4, DBL5	T1/T2/T3 and delivery^m^	PM, LBW, PTB, PI
Gnidehou, 2014 [58]	Cordoba, Colombia	DNS	Low	CC (55)^n^	All; 16	21 (6)	No	VAR2CSA: DBL5ε, DBL3X, ID1-ID2	T2/T3/delivery	PI
Guitard, 2008 [59]	Thiadiaye, Senegal	2001	Low	cohort (261)	All^o^; 22.3	24.1 (6.1)	No	VAR2CSA DBL5ε	T1/T2 and delivery	PM
Lloyd, 2018 [60]	Yaounde, Cameroon	1995–2001	Intermediate	CC (1377)^p^	All; 35.7	25.8 (5.9)	No	VAR2CSA: FV2	Delivery	Anaemia^q^, PM, PTB, and LBW.
McLean, 2017 [63]	Madang, PNG	2005–2007	Low	CS from cohort (204)	All; 43.2	median (IQR) 24 (21–28)	Chloroquine prophylaxis	VAR2CSA: DBL5^r^	Delivery	PM
Megnekou, 2005 [43]	Etoa and Yaounde, Cameroon	1996–1998	Etoa: High; Yaounde: Intermediate	Cohort (Etoa 29; Yaounde 186)	All; Yaounde 47, Etoa 48	Yaounde range 15–40; Etoa range 14–38	< 40% used chemoprophylaxis	pRBC: FCR3	T1/T2, T2/T3^s^	PI
Staalsoe, 2001 [35]	Ebolowa and Yaounde, Cameroon	1992–1996	Ebolowa: High; Yaounde: Intermediate	Ebolowa: CS (113); Yaounde: CS (45);	Ebolowa: All; 48.7 Yaounde: SG/MG	DNS	Some chemoprophylaxis	pRBC: Palo Alto	Delivery	PM
Staalsoe, 2004 [61]	Kilifi, Kenya	1996–1997	Intermediate	CS (477)^t^	All; 22.2	Range 14–35+	No	pRBC: EJ24	Delivery	PM^u^, PI, SA^v^, LBW
Teo, 2014 [62]	Blantyre, Malawi	1999–2006	Intermediate	Malawi: CS (332)	SG/MG	Median (IQR) 25 (21–27)	SP	pRBC: CS2, VAR2CSA DBL5Ɛ	Delivery	LBW^w^

Abbreviations: *CC* case control, *CS* cross sectional, *LBW* low birthweight, *MG* multigravidae, *PI* peripheral infection, *PM* placental malaria, *PG* primigravidae *PTB* preterm birth, *SA* severe anaemia, *SG* secundigravidae, *SM* severe malaria, *T1* first trimester, *T2* second trimester, *T3* third trimester

^a^Endemicity as reported in original paper, in paper referenced in original paper, or as estimated for the latitude and longitude of study site and first year of enrolment using data from Malaria Atlas Project (*P. falciparum* parasite rate in 2–10 year olds globally, 2000–2017). If study was conducted before 2000 and information on endemicity was not provided in the paper, then the P. f. PR2–10 for the year 2000 was used

^b^Age is presented as mean (SD) except where indicated

^c^This table includes only those Ab responses that met the inclusion criteria of the review. In some studies, additional Ab responses, Ab measurement time points, and subclass responses were reported in the original paper. Unless indicated otherwise, estimates for total IgG responses were included. Details of antigens are included in Additional file 6

^d^Herein we tabulate only those outcomes that met the inclusion criteria of this review and were available in categorical format (positive or negative for outcome)

^e^Study population was a subset of women randomly selected from the Lungwena Antenatal Intervention Study (LAIS) cohort in which women received IPTp (sulfadoxine-pyramethamine) on at least 2 occasions and some women also received two doses of azithromycin on two occasions

^f^Women were enrolled at 14–26 week gestation (T2); Antibodies were also measured at 28–34 week gestation (T3) and at 1, 3, and 6 months postpartum

^g^Anaemia was defined as haemoglobin < 110 g/l

^h^Samples were originally from a cross-sectional study (total 1944 samples): All samples from PM^+^ women (*n* = 116) and 348 randomly selected samples from PM^−^ women were selected from women who had ≥ 3 pregnancies, were ≥ 20 years of age, and had term or premature deliveries. Authors reported that all PM^−^ women had been exposed to malaria since they had Ab to DBL5

^i^Infected women were individually matched by gravidity, age (± 2 years), and date of delivery (± 2 months) to women without evidence of current or previous placental infection according to the results of placental histological analysis and placental, peripheral, and cord blood smears, and/or to women with evidence of past placental infection (malaria pigment present by placental histological analysis but no parasites seen, together with negative placental, peripheral, and cord blood smears)

^j^Pregnant women who were blood film positive and diagnosed with one or more of the following clinical manifestations were categorized as having SM (*n* = 39): severe anaemia, haemoglobin levels < 7 g/dL; cerebral malaria, unrousable coma; hypoglycaemia, blood glucose levels < 40 mg/dL; hypotension, systolic blood pressure < 90 mmHg; jaundice, physical diagnosis or bilirubin levels > 3 mg/dL and hyperparasitaemia, > 10,000 parasites/μl. Pregnant women who were parasitaemic without these features were defined as uncomplicated malaria (UM; *n* = 41). An additional 41 pregnant women with negative blood films and no signs of clinical malaria were enrolled as uninfected controls. Estimates included in this review used uninfected women as the comparator group

^k^Trial of maternal vitamin A supplementation

^l^Participants were identified from 1000 pregnant Karen women who participated in a placebo randomized controlled trial of chloroquine prophylaxis against *P. vivax* infection. Cases (*n* = 136) in the nCC study included all women with *Plasmodium* infection detected at any time during pregnancy and controls (*n* = 331) were randomly selected from the 864 women with no detectable parasitemia at any time during pregnancy. Antibodies were measured fortnightly from enrolment to delivery, but only antibody responses at enrolment are included in estimates

^m^Women were enrolled up to T3; also measured antibodies at T3 (weeks 30–32), but these estimates are not presented in this review

^n^Pregnant women who were parasite positive (by quantitative PCR) and pregnant women who were parasite negative (quantitative PCR negative) were enrolled during T2 or T3 or at delivery

^o^Antibody responses were only analysed in women who had at least one detected malarial infection during follow-up

^p^Study was conducted using archival samples from pregnant women residing in Yaoundé, Cameroon, including 341 PM^+^ women and 1036 PM^−^ women

^q^Anaemia was defined as < 30% packed cell volume

^r^Estimates included in this review are for IgG3 responses because total IgG was not reported in original paper

^s^Time points correspond to first- (6–29 weeks) and third- (25–41 weeks) antenatal visits; antibodies were also measured at second visit (16–36 weeks) but data is not included in this review

^t^Women selected from a larger cohort of 910 women on the basis of HIV-1 status, gravidity, and placental histology

^u^Original paper grouped women according to placental histology; for this review we combined women who had acute infection with those who had chronic infection, but excluded those with ‘past’ infection

^v^Severe anaemia defined as Haemoglobin < 70 g/l

^w^Only samples from uninfected women were included

**Table 2:** Characteristics of studies described in narrative terms in the systematic review

Author, year	Region, country	Enrolment period	Endemicity	Study design (*n*)	Gravidity; % PG	Mean (SD) age^a^	IPTp use	Antibody responses	Ab time	Clinical outcomes^b^	Key findings relating to review inclusion criteria
Ataide, 2010 [76]^c^	Blantyre, Malawi	2000–2002	Intermediate	CS (263)^d^	PG	20.0 (3.3)	SP	pRBC: CS2 (total IgG and phagocytic Abs)	T3	PM	Total IgG and phagocytic Abs higher in PM+ than PM−
										LBW	No association with Ab responses
										Anaemia^e^	No association with Ab responses
Ataide, 2011 [64]^c^	Blantyre, Malawi	2000–2002	Intermediate	CS (187)^f^	SG	20.0 (3.3)	SP	pRBC: CS2 (total IgG and phagocytic Abs)	T3	PM	Total IgG and phagocytic Abs higher in PM+ than PM−
										Hb	No correlation of IgG or phagocytic Abs w/ Hb.
										Birthweight	+ve correlation between phagocytic Abs (but not total IgG) and birthweight
Babakhanyan, 2015 [65]	Yaounde, Cameroon	1996–2001	Intermediate	CC (420)^g^	MG	PM+ : 27.6 (4.6); PM- 29.4 (5.6)	No	VAR2CSA: FV2, DBL1; DBL2; DBL1+2; DBL3, DBL4; DBL5; DBL6; DBL4	Delivery	PM	Ab levels similar in PM+ and PM- women; proportion of high avidity FV2 Abs was higher in PM- than PM+ women
Chandrasiri, 2016 [66]	Mangochi District, Malawi	2011–2012	High	RCT (1002)^h^	All; 19.9)	24.5 (5.8)	SP	pRBC: CS2 (total IgG and opsonizing Abs)	T2	Hb at 36 weeks	+ve association between total IgG and opsonizing Abs to CS2 and Hb at 36 gw
										Birthweight	No association
Feng, 2009 [67]	Blantyre, Malawi	2003–2004	Intermediate	RCT (141)^i^	All; 56.7)	21.2 (4.7)	SP, SP + azithromycin or SP + artesunate	pRBC: CS2 (total IgG and opsonizing Abs)	T2	Anaemia^e^	Both higher levels of IgG and opsonic phagocytosis to CS2 were associated with decreased anaemia at delivery
										LBW	No association
Hommel, 2010 [68]	Blantyre, Malawi	1998–2000	Intermediate	CC (62)^j^	All; DNS	PM+ 20.9, PM- 21.5	SP	pRBC: Pf2006-CSA; Pf2004-CSA;3D7-CSA; HCS3; HB3-CSA; XIE-CSA CS2	Delivery	PM	PM+ PG had had higher Ab levels to isolates than PM- PG. Difference not observed for MG PM+ versus MG PM- women.
Khattab, 2004 [34]	Lambaréné, Gabon	2002	Intermediate	CS (151)	All; 27.2	PG 19.1 (1.9), MG 22.9 (4.0)	DNS	pRBC: Gb218, Gb337,vip43, and vip42	Delivery	PM	PM+ PG had higher Ab levels than PM- PG; association not observed for MG.
Mayor, 2011 [69]	Manhiça, Mozambique	2003–2005	Intermediate	CS from RCT^k^ (90)	All; 33.3	PG 19.1 (1.9); MG 22.9 (4.0)	Placebo group for IPTp trial	pRBC:193 T, CS2, FCR-CSA, Plac1–4, Mot1–8	Delivery	PM	PM+ had higher Abs to all parasites tested.
Mayor, 2013 [70]	Manhiça, Mozambique	2003–2005	Intermediate	CS from RCT (293)^l^	All; 27	Placebo 24.3 (6.6); SP 24.0 (6.6)	Randomized to SP or placebo	pRBC: Plac1, Plac2 VAR2CSA: DBL3X, DBL2X, DBL5ε, DBL6ε	Delivery	PM	PM+ had higher Abs against all pRBC and antigens tested.
										PI	Abs levels not associated with PI after adjusting for PM.
										Birthweight	Higher Abs against DBL2X and Plac2 associated with lower birthweight; Among women w/. ≥ 1 malaria episode during pregnancy, high Abs to Plac2, DBL3X and DBL6ε associated with increased BW.
										GA	Higher Abs against DBL2X associated with younger GA; Among women w/. ≥1 malaria episode during pregnancy, high Abs to Plac2, DBL3X and DBL6Ɛ associated with increased GA.
										Hct	No association
O’Neil-Dunne, 2001 [38]	Yaounde, Cameroon	DNS	Intermediate	CS (198)	All; 23.7	G1: 20; G2: 22; G3: 24; G ≥ 5: 31	No	pRBC: 3D7 (CSPG adhesion inhibitory Abs)	Delivery	PM or PI: “malaria”	Malaria+ve/−ve women had similar levels of anti-adhesion Abs. Malaria +ve women w/ high anti-adhesion Abs had lower levels of placental parasitaemia.
Salanti, 2004 [27]	Kilifi, Kenya	1996–1997	Intermediate	CS (110)	All; DNS	DNS	No	VAR2CSA: DBL5ε	Delivery	Birthweight	PM+ women w/. high levels of anti- DBL5ε IgG gave birth to heavier babies.
Serra-Casas, 2010 [71]^m^	Manhiça, Mozambique	2003–2005	Intermediate	RCT (302)	All; 24	Placebo: 23.9; SP: 24.6	Randomized to SP (152) or placebo (150)	pRBC: CS2	Delivery	PM	PM+ women had higher Abs to CS2 than PM- women
										PI	PI+ women at delivery had higher Abs to CS2 than PI- women
										Anaemia^n^	No association
										LBW	No association
										PTB	No association
Siriwardhana, 2017 [72]	Yaoundé, Cameroon	1996–2001	Intermediate	CS (1377)	All; 35.7	25.8 (5.9)	No	VAR2CSA: FV2, DBL1+2, ID1-ID2a, DBL1, DBL2, DBL3, DBL4, DBL5, DBL6	Delivery	PM	PM+ women recognized more DBL domains than PM- women and had higher IgG to FV2, DBL1+2, DBL2, DBL3, DBL4, DBL5, DBL6 and ID1-ID2a (3D7).
Tuikue Ndam, 2006 [39]	Thiadiaye, Senegal	2001	Low	cohort (275)	All; 21.8	24.1 (6.1)	No	VAR2CSA: DBL5ε, DBL6ε, DBL1-x at	T1/T2, delivery	PM	PM+ women had higher Ab levels than PM- women at delivery but no difference in Ab levels at T1/T2.
										Birthweight	No significant association with Abs
										Anaemia^o^	No significant association with Abs
Tuikue Ndam, 2015 [73]	Comé, Benin	2008–2010	High	cohort (710)	All; 18.2	26.4 (6.2)	SP	pRBC: FCR3 (CSPG binding inhibition and total IgG); VAR2CSA: FV2, DBL1-2, DBL3, DBL4, DBL5, DBL6	T1/T2, delivery	PM	High DBL3 Abs at T1/T2 associated with reduced prevalence of PM at delivery. Trend between strong anti-FCR-3 VSA Abs and reduced prevalence of PM. For PI –ve women at inclusion, higher CSPG-binding inhibitory capacity at delivery (but not T1/T2) was associated with lower risk of PM.
										PI	High FV2 and DBL3 Abs at T1/T2 associated w/ reduced risk of PI during pregnancy
										LBW	High Abs to DBL1-DBL2 and DBL3 at T1/T2 were associated with reduced prevalence of LBW babies; High CSPG-binding inhibitory activity at delivery but not T1/T2 associated with reduced risk of LBW. No association with total IgG to FCR3
										PTB	For women PI –ve at inclusion, higher CSPG-binding inhibitory capacity, but not total IgG, associated with lower risk of PTB.
										Anaemia^e^	No significant association with Abs
Tutterrow, 2012a [74]	Ngali II and Yaounde, Cameroon	2001–2005	Ngali II: High Yaounde: intermediate	cohort (Ngali II 27; Yaounde 48)^p^	All; Ngali 33.3; Yaounde 34.0	Ngali II: 23.2 (5.4); Yaounde: 25.0 (5.1)	CQ prophylaxis	VAR2CSA: DBL1, DBL3, DBL4, DBL5, DBL6, DBL1+2	T1, T2, T3	PM	PM- women from high transmission Ngali II, but not low transmission Yaoundé had higher Ab levels to DBL3, DBL4, DBL5, and DBL6 domains, but not to DBL1, throughout pregnancy, compared to PM+ women from the same village.
Tutterrow, 2012b [75]	Ngali II and Yaounde, Cameroon	2001–2005	Ngali II: High Yaounde: intermediate	cohort (Ngali II 27; Yaounde 48)^p^	All; Ngali 33.3; Yaounde 34.0	Ngali II: 23.2 ± 5.4; Yaounde: 25.0 ± 5.1	CQ prophylaxis	pRBC: 7G8; VAR2CSA: FV2	T1, T2, T3	PM	PM- had higher Abs to FV2 (FCR3) than PM+ women throughout pregnancy. In Ngali II women, high avidity FV2 Abs at T2 were associated with reduced risk of PM.

Abbreviations: *Abs* antibodies, *CSPG* chondroitin sulfate proteoglycan, *GA* gestational age, *Hb* haemoglobin, *Hct*, haematocrit, *LBW* low birthweight, *MG* multigravidae, *PI* peripheral infection,

*PM* placental malaria, *PG* primigravidae, *PTB* preterm birth, *SG* secundigravidae, *T1* first trimester, *T2* second trimester, *T3* third trimester

^a^Age is presented as mean (SD) except where indicated

^b^We only tabulate those antibody responses and clinical outcomes that met the inclusion criteria of this review. In some studies, other antibody responses and outcomes were measured. Where outcomes were presented as both continuous and categorical variables in original papers (e.g. birthweight and low birthweight), we report on findings for the categorical variable

^c^Ataide et al. [76] and [64] describe the same study cohort, with the gravidity of subjects differing between publications (as specified)

^d^Women were selected based on HIV status, thus 40% of women in this study were HIV positive

^e^Anaemia defined as haemoglobin < 110 g/l

^f^Women were selected based on HIV status, thus 65% of women in this study were HIV positive

^g^Retrospective case-control study using archival plasma samples from a cross-sectional study. All PM^+^ women meeting inclusion criteria (≥ 3 pregnancies, 18 years or older, singleton live deliveries; babies that were > 28 weeks of gestation, women positive for Ab to FV2) were included (*n* = 96). For comparison, a random selection of PM− women that met the inclusion criteria were included (*n* = 324)

^h^Trial of maternal nutrient supplementation to increase birth weight

^i^Women who were 14–26 weeks pregnant and were positive for peripheral parasitaemia by blood film examination were eligible to participate in trial and were randomized to one of three antimalarial treatment groups

^j^Same matched case-control study as Beeson et al. [53]. Infected women were individually matched by gravidity, age (± 2 years), and date of delivery (± 2 months) to women without evidence of current or previous placental infection according to the results of placental histological analysis and placental, peripheral, and cord blood smears, and/or to women with evidence of past placental infection (malaria pigment present by placental histological analysis but no parasites seen, together with negative placental, peripheral, and cord blood smears)

^k^Subgroup of women randomly selected from women who received placebo in IPTp trial

^l^This study included the last 200 women who received SP or placebo in a randomized controlled trial and delivered in 2005, plus a random selection of 20% of the women who received placebo and delivered in 2003 or 2004

^m^Same IPTp trial as Mayor et al. [69] and [70]

^n^Maternal anaemia was defined as haematocrit < 33%

^o^Definition of anaemia not shown

^p^All women had confirmed malaria infection during ≤ 6 months of pregnancy based on slide or PCR data

### Placental infection

**Fig. 2:**
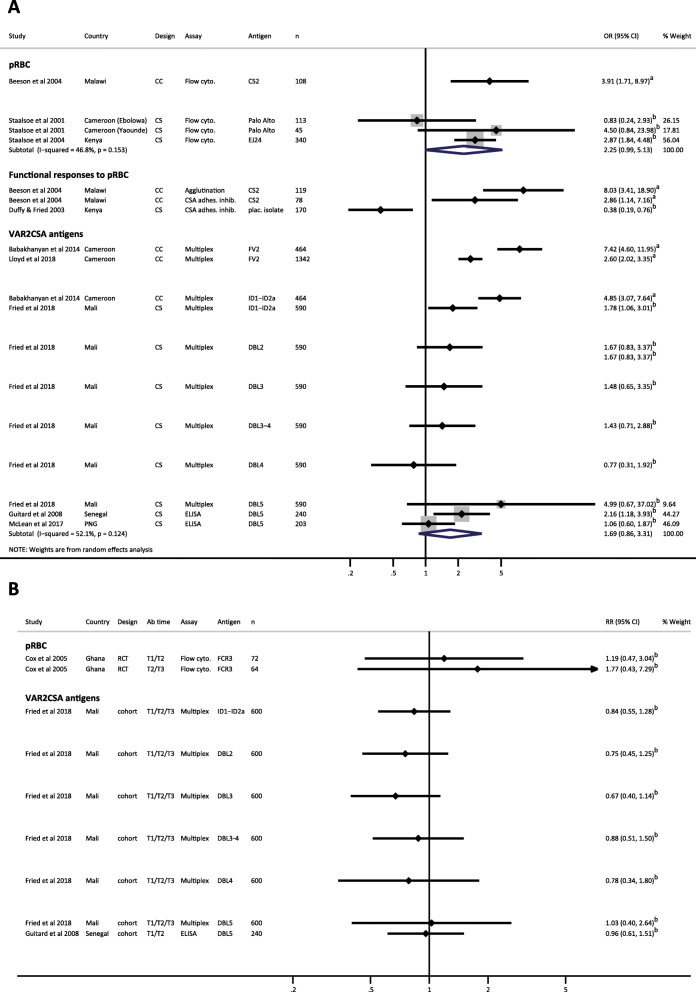
Forest plot of the association between antibodies to pregnancy-associated *P. falciparum* antigens and placental malaria. **a** Estimates represent the odds of placental malaria in Ab responders compared with Ab non-responders, where antibodies were measured at delivery (cross-sectional studies). **b** Estimates represent the relative risk of placental malaria in Ab responders compared to Ab non-responders, where antibodies were measured at various times during pregnancy, as indicated (prospective studies). Estimates are for all gravidities included in original studies. Staalsoe et al. (Yaounde site) included secundigravidae and multigravidae only; Babakhanyan et al. [52] included multigravidae only; and Cox et al. [55] included primigravidae only. Estimate for McLean et al. [63] represents IgG3 responders only as total IgG was not available. Meta-analysis was only performed on estimates where VAR2CSA antigen or functional assay (where applicable) and timing of antibody determination were the same. Meta-analysis of Ab responses to FV2 and odds of PM showed a high degree of heterogeneity (*I*^2^ = 93.1%, *P* < 0.001) so results were not pooled. ^a^Estimate calculated by current authors from data in original publication; ^b^Data supplied by original authors and estimate calculated by current authors. CC, case-control; CS, cross-sectional; CSA adhes. inhib., CSA adhesion inhibition assay; flow cyto., flow cytometry; n, number of participants included in estimate; OR, odds ratio; plac. isolate, placental isolate; pRBC, parasitized red blood cells; RCT, randomized controlled trial; RR, risk ratio

We included estimates from nine studies that investigated the association between antibodies (Abs) to pregnancy-specific pRBC or VAR2CSA domains measured at delivery, and placental infection (Fig. 2a) [35, 42, 52, 53, 56, 59–61, 63]. In all studies, placental infection was confirmed by slide microscopy of placental blood or placental histology. Most studies demonstrated either no difference in the odds of placental infection in antibody responders compared to non-responders, or increased odds of placental infection in antibody responders at delivery (Fig. 2a). Total IgG responders to variant surface antigens (VSA) on CSA-adherent parasite lines had increased odds of placental infection in pooled estimates from cross-sectional studies [35] [61] (reOR = 2.25, 95% CI 0.99–5.13, *I*^2^ = 46.8%) (Fig. 2a) and in a case-control study (OR 3.91, 95% CI 1.71–8.97) [53]. Functional antibodies to CS2 (agglutinating, CSA adhesion inhibitory) were associated with increased odds of placental infection [53] in a Malawian case-control study (OR of 8 and 2.9 fold, respectively). In contrast, a Kenyan cross-sectional study showed that women who had antibodies that could inhibit adhesion of a placental isolate to CSA had a 62% reduction in odds of placental infection (OR 0.38, 95% CI 0.19–0.76) [56] (Fig. 2a). Antibody responders to FV2, and VAR2CSA domains, with the exception of DBL4, had increased odds of placental infection compared to non-responders [42, 52, 59, 60, 63] (Fig. 2a). Estimates for women sub-grouped by parity showed similar patterns of association between antibody responses at delivery and placental infection (Additional file 7: Figures S4A and S5A).

Twelve studies included in narrative terms measured antibodies at delivery or in the third trimester and examined placental infection as an outcome [34, 38, 39, 64, 65, 68–73, 76]. Nine of these studies found that Abs to pregnancy-specific pRBC and VAR2CSA antigens were positively associated with placental infection or placental parasite density [34, 39, 64, 68–76, 76], but in some studies, this relationship was restricted to gravidity group, most commonly in primigravidae [34, 68] (Table 2). One study reported no significant difference in total levels of VAR2CSA antibodies at delivery, but higher levels of high avidity Abs to FV2 in women who were negative for placental infection compared to those who were positive for placental infection (Table 2) [65]. A Cameroonian cross-sectional study reported that among malaria-positive women, those with high anti-adhesion Abs had reduced placental parasitaemia, but levels of anti-adhesion Abs were similar between women positive and negative for malaria [38]. To summarize, evidence from studies included in narrative terms suggests that whilst high avidity Abs and anti-adhesion Abs measured at delivery may be associated with protection from placental infection [65] and reduced placental parasitaemia [38], respectively, total IgG responses to VAR2CSA antigens and pregnancy-specific pRBC are positively associated with the presence of placental malaria [34, 39, 64, 68–72, 76].

Three prospective studies, including one RCT [55] and two cohort studies [42, 59], provided estimates for the association between antibody responses measured during pregnancy and risk of placental infection, but no clear pattern of association was found (Fig. 2b and Additional file 7: Figures S4B and S5B). Of the three cohort studies included in narrative terms, two provided evidence for an association between Ab responses to some, but not all VAR2CSA domains, and pRBC strains, measured earlier in pregnancy, and reduced risk of placental infection [73, 75], and one indicated no difference in Ab levels to VAR2CSA domains measured in first or second trimester (T1/T2) among women who were negative or positive for placental infection (Table 2) [39]. Overall, the majority of estimates included in this review, and studies included in narrative terms, indicate that when measured at delivery, antibody responses to pregnancy-specific pRBC and VAR2CSA antigens are associated with the presence of placental infection and may therefore represent markers of infection, rather than correlates of protection. Of the five studies that measured antibodies earlier in pregnancy, and followed women until delivery, two provided evidence for a protective effect of anti-VAR2CSA antibodies [73–75] and three found no significant association with placental infection [42, 55, 59].

### Peripheral *P. falciparum* infection

**Fig. 3:**
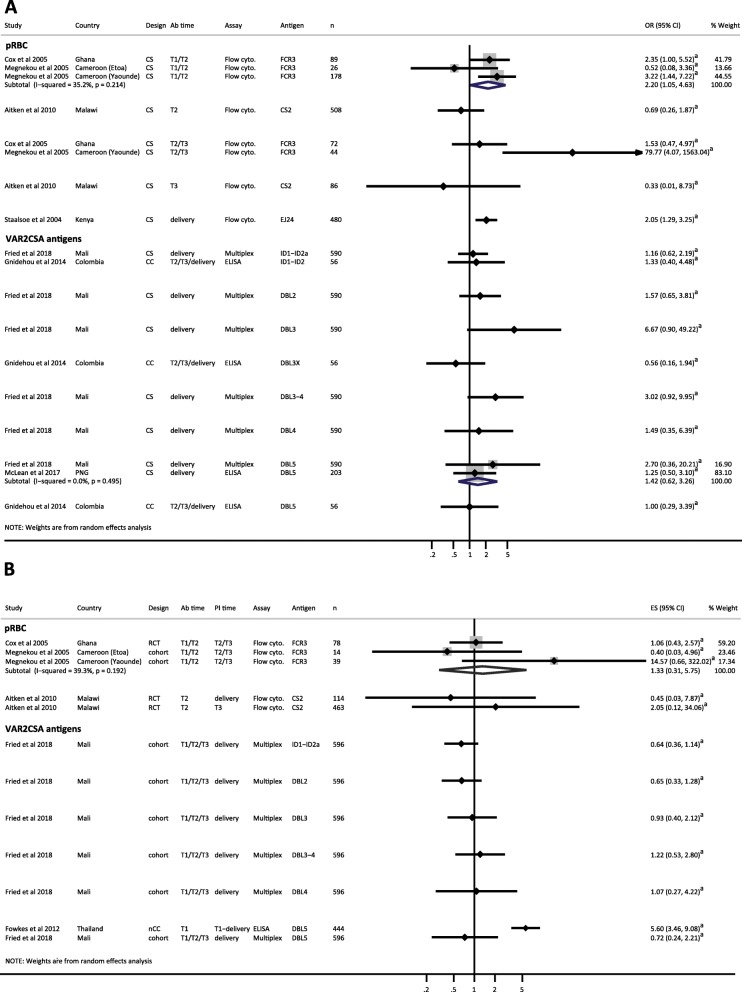
Forest plot of the association between antibodies to pregnancy-associated *P. falciparum* antigens and peripheral parasitaemia. **a** Estimates represent the odds of peripheral *P. falciparum* parasitaemia in Ab responders compared to Ab non-responders, where antibodies were measured at the same time point as parasitaemia (cross-sectional studies). **b** Estimates of peripheral *P. falciparum* parasitaemia in Ab responders compared to Ab non-responders, where antibodies were measured at time points prior to parasitaemia determination. Estimates are risk ratios for RCT and cohort studies and odds ratio for the nested case-control study. Estimates are for women of all gravidities included in original studies: Cox et al. [55] included primigravidae only. The timing of antibody and parasitaemia determination is indicated. Estimate for McLean et al. [63] represents IgG3 responders only as total IgG was not available. DBL5 estimates were only combined when study design was the same. Estimates for FCR3 responses at T2/T3 were not combined because *I*^2^ = 82.9%. ^a^Data supplied by original authors and estimate calculated by current authors; ^b^Estimate calculated by current authors from data in original publication. CC, case control; CS, cross-sectional; ES, estimate; n, number of participants included in estimate; Flow cyto., flow cytometry; nCC, nested case-control; OR, odds ratio; PI time, timing of determination of parasitaemia; pRBC, parasitized red blood cells; RCT, randomized controlled trial; T1, first trimester; T2, second trimester; T3, third trimester

Seven studies provided cross-sectional estimates for the association between antibody responses to pregnancy-specific antigens, measured at various time points, and peripheral *P. falciparum* infection, yielding heterogeneous results (Fig. 3a and Additional file 7: Figures S6A and S7A) [42, 43, 51, 55, 58, 61, 63]. For many of the antigens examined, antibody responders had increased odds of peripheral infection compared to non-responders, but these associations were rarely significant. Early in pregnancy (T1/T2), antibody responders to CS2-adherent FCR3 had increased odds of peripheral infection in pooled estimates from cross-sectional studies (reOR 2.20, 95% CI 1.05–4.63, *I*^2^ = 35.2%) (Fig. 3a) [43, 55]. Similarly, antibody responders to a placental isolate at delivery had twofold increased odds of peripheral infection (OR 2.05, 95% CI 1.29–3.25) [61].

Three studies included in narrative terms examined peripheral *P. falciparum* infection as an outcome [70, 71, 73]. In an IPTp trial in Mozambique, women who were parasitaemic at delivery had higher Abs to CS2 than non-parasitaemic women [71], but antibody levels to a suite of placental isolates and VAR2CSA domains were not associated with peripheral infection after adjusting for placental malaria [70]. In women from Benin, high FV2 and DBL3 Abs early in pregnancy (T1/T2) were associated with reduced risk of peripheral infection during pregnancy, but this association was not observed for the four other VAR2CSA domains analysed nor to CSPG-binding inhibitory Abs to FCR-3 [73].

In addition to studies that provided cross-sectional estimates, four prospective studies and one nested case-control study provided estimates for the association between antibody responses earlier in pregnancy, and risk of peripheral parasitaemia later in pregnancy or at delivery (Fig. 3b and Additional file 7: Figures S6B and S7B) [42, 43, 51, 55, 57]. One study found that Thai women positive for antibodies to DBL5 in the first trimester had increased odds of having a peripheral infection during pregnancy (OR 5.60, 95% CI 3.46–9.08) [57], but the remaining studies showed no clear association between positive Ab responses to pregnancy-specific antigens and prospective risk of peripheral infection [42, 43, 51, 55].

In summary, whilst some studies found an association between Ab responses to pregnancy-specific pRBC and VAR2CSA antigens and presence of parasitaemia, either concurrently or at a later time point in pregnancy, we found no evidence for a protective association between these antibodies and peripheral parasitaemia. Thus, antibodies to VAR2CSA either early in pregnancy or at delivery do not appear to reduce the incidence or level of peripheral parasitaemia throughout pregnancy.

### Low birthweight

**Fig. 4:**
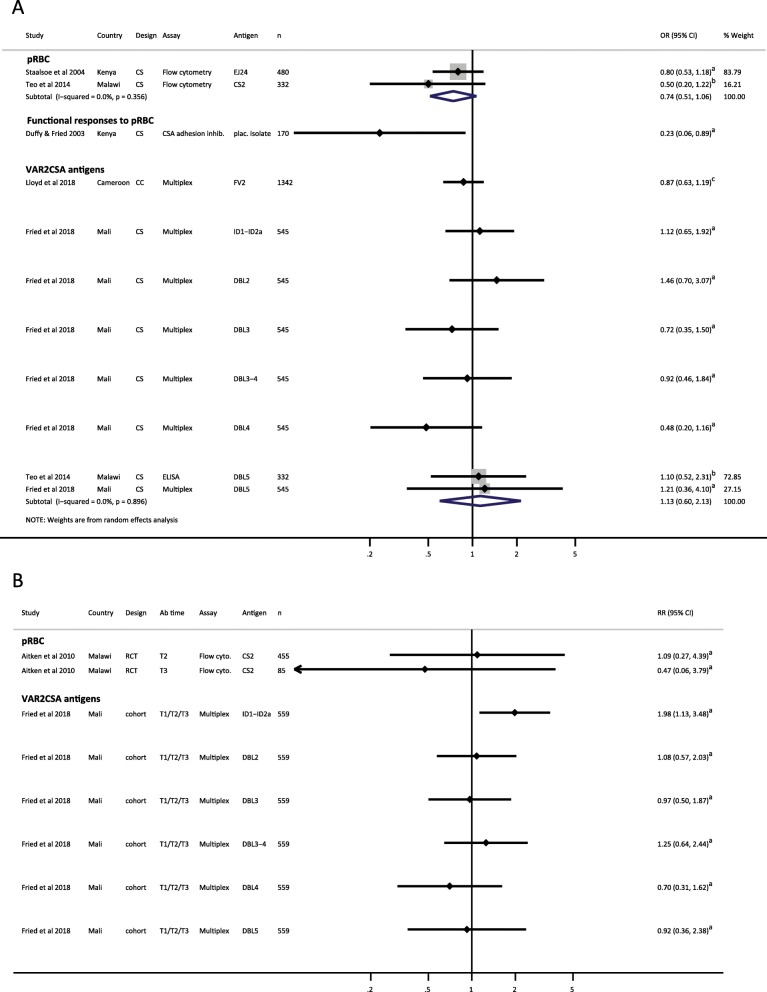
Forest plot of the association between antibodies to pregnancy-associated *P. falciparum* antigens and low birthweight. **a** Estimates represent the odds of low birthweight birth in Ab responders compared with Ab non-responders, where antibodies were measured at delivery (cross-sectional studies). **b** Estimates represent the risk of low birthweight in Ab responders compared with Ab non-responders, where antibodies were measured at time points prior to delivery, as indicated (prospective studies). Estimates are for women of all gravidities included in the original studies. Teo et al. included secundigravidae and multigravidae only. ^a^Data supplied by original authors and estimate calculated by current authors; ^b^Estimate provided by original author; ^c^Estimate calculated by current authors from data in original publication. CC, case-control; CS, cross-sectional; n, number of participants included in estimate; OR, odds ratio; pRBC, parasitized red blood cells; RCT, randomized controlled trial; RR, risk ratio; T1, first trimester; T2, second trimester; T3, third trimester

The association of antibodies to pregnancy-specific pRBC, measured at delivery, and low birthweight was examined in three studies (Fig. 4a and Additional file 7: Figures S8A and S9A): two studies measured total IgG antibodies [61, 62] and one study measured CSA adhesion inhibitory antibodies [56]. Pooled analyses indicated that compared to non-responders, IgG responders to CSA-binding pRBC, measured at delivery, had a clinically significant 26% reduction in the odds of low birthweight delivery (reOR = 0.74, 95% CI 0.51–1.06, *I*^2^ = 0.0%), but the confidence interval was wide and captured a scenario of a small increase in odds of low birthweight (Fig. 4a) [61, 62]. Kenyan women with anti-CSA adhesion activity had an 77% reduction in odds of low birthweight birth [56] compared to women who did not have anti-CSA adhesion activity. This association was also observed in sub-group analysis of secundigravidae/multigravidae (Additional file 7: Figure S9A) but not among primigravidae (Additional file 7: Figure S8A), most likely because an insufficient number of women had acquired anti-adhesion activity in their first pregnancy in this study population. In contrast, there was no significant pattern of association between antibody responses to VAR2CSA antigens measured at delivery and low birthweight (Fig. 4a) [42, 60, 62]. Estimates stratified by gravidity gave similarly heterogeneous results for association between antibodies to VAR2CSA at delivery and low birthweight (Additional file 7: Figures S8A and S9A).

When antibodies were measured earlier in pregnancy, estimates from one study of Malian women indicated that Ab responders to ID1-ID2a but not DBL2, DBL3, DBL3–4, DBL4, and DBL5 had increased risk of low birthweight births (RR 1.98, 95% CI 1.13–3.48) compared to non-responders (Fig. 4b). In a sub-group analysis, this association remained significant in primigravidae (Additional file 7: Figure S8B) but not multigravidae (Additional file 7: Figure S9B) [42].

Of the studies included in narrative terms, high antibody responses to some VAR2CSA antigens including DBL5ε [27], DBL1-DBL2, and DBL3 [73] and functional antibodies to pRBC [64, 73], were associated with reduced risk of low birthweight (or Ab levels were correlated with birthweight), but these associations were dependent on the timing of antibody determination [73] and the gravidity of subjects [64]. In a Senegalese cohort, no significant association was found for antibodies to DBL1-x, DBL5ε, and DBL6ε, measured at T1/T2 and delivery, and low birthweight [39] (Table 2).

In summary, several studies provided evidence for an association between antibody responses to pregnancy-specific *P. falciparum* antigens and decreased risk of low birthweight [27, 56, 61, 62, 64, 73].

## Discussion

In this systematic review and meta-analysis, we aimed to synthesize the existing epidemiological evidence for the association between antibodies to pregnancy-specific antigens and the risk of malaria in pregnancy and its associated adverse maternal and birth outcomes. Overall, we found that estimates for the association between pregnancy-specific *P. falciparum* antibody responses and the pregnancy and birth outcomes examined were heterogeneous in terms of both the direction and magnitude of effect. Whilst antibody responses to several antigens were positively associated with the presence of placental and peripheral infections, this review did not identify evidence that any specific antibody response is associated with protection from PAM and its clinical consequences across multiple populations.

Cross-sectional estimates for the association between antibody responses and *P. falciparum* infections during pregnancy suggest that antibody responses may serve as markers of current infections. Indeed, previous studies have reported a concurrent increase in pregnancy-specific antibodies in women with placental infection [53, 61, 69, 70]. We found that positive antibody responses to pregnancy-specific antigens were associated with increased odds of peripheral infection in some studies, with varying degrees of significance [43, 55, 57, 61]. At delivery, antibody responders (IgG) to the pRBC surface [35, 53, 61], to full-length VAR2CSA (FV2) [52, 60], and to the vaccine candidate ID1-ID2a, which includes interdomain region 1, DBL2X, and interdomain region 2a, had increased odds of placental infection compared to non-responders [42, 52] (Fig. 2a). In contrast, antibodies to single domains (DBL2, DBL3, DBL4, DBL5), measured at delivery, were not associated with placental infection (Fig. 2a). This finding suggests that reactivity to VAR2CSA epitopes in their native formation may be more relevant correlates of disease than responses to individual recombinant domains. Indeed, previous in vitro studies using the full-length extracellular VAR2CSA have suggested that the overall folding of the protein may be important for ability to bind CSA [77, 78]. Furthermore, it has been suggested that functional antibodies to VAR2CSA may have affinity to conformational epitopes not displayed by individual recombinant constructs from single alleles [79]. Further studies designed to measure antibody responses to multiple VAR2CSA antigens concurrently, will be essential to confirm which antibody responses are the best markers of infection and could therefore be utilized as serosurveillance tools [80].

An effective vaccine against placental malaria should induce broadly active and strain-transcending antibodies to block the adhesion of VAR2CSA-expressing parasites to CSA [81, 82]. A single cross-sectional study found that women positive for CSA-binding inhibitory antibodies had significantly decreased odds of placental infection, as well as low birthweight and preterm birth [56]. Although two vaccines based on the N-terminal CSA-binding region of VAR2CSA are currently in early clinical trials, this review highlights a scarcity of evidence for a protective association between antibodies to recombinant VAR2CSA domains, including the vaccine candidates, and placental malaria. Only two prospective cohort studies, included in narrative terms, found that antibody responses to selected VAR2CSA antigens and pRBC were associated with protection from placental infection [73–75] and responses to individual recombinant VAR2CSA antigens were associated with protection against low birthweight and preterm birth in individual studies only [27, 73]. None of these studies reported an association between antibodies to the vaccine candidates (ID1-DBL2X-ID2a and DBL1X-DBL2X) and protection from placental malaria, low birthweight, or preterm birth. Interestingly, Malian primigravidae who were positive for antibodies to ID1-ID2a at enrolment (during T2/T3) were more likely to deliver preterm babies, suggesting that this antigen may be a marker of parasite exposure [42]. It is likely that protection from placental malaria and its associated adverse pregnancy outcomes develops with the acquisition of an increasingly broad antibody response to different VAR2CSA domains and allelic variants, rather than to a single domain [74, 75]. Furthermore, high avidity antibodies and functional antibody responses are probably more important measures than simple quantification of IgG responses to recombinant proteins [65, 75].

In the absence of a robust, cross-population estimate of the effect of pregnancy-specific *P. falciparum* antibody responses, we are unable to quantify the fraction of clinical disease or adverse birth outcomes that may be averted by these immune responses. This has implications for understanding any impact of potential vaccines based on pregnancy-specific antigens on the burden of pregnancy-associated malaria in a given population. Only nine of the studies examined antibody responses to multiple recombinant antigens concurrently in the same populations [39, 42, 52, 58, 65, 70, 72–74], and only three of these contributed estimates in the format required for meta-analysis [42, 52, 58]. This precluded assessment of the relative clinical importance of antibody responses to individual VAR2CSA domains.

A key strength of this systematic review was that we contacted authors directly, and for 13 studies, we were able to obtain estimates that were not originally published. A limitation was the heterogeneity observed between estimates, which is likely due to methodological and epidemiological differences between the included studies. We included studies that collectively examined a broad range of antibody responses, including to the surface of pRBC isolates from infected placentas, to CSA-binding pRBC strains, and to recombinant VAR2CSA antigens. With respect to the antigens examined, parasite isolates differed in their geographical origin and recombinant antigens varied by allele, domain boundary, and expression system (Additional file 6), potentially impacting variation in antibody reactivity between studies [83, 84]. Importantly, the influence of the global genetic diversity of the var2csa gene on antibody reactivity, and consequently, vaccine development, has not been adequately determined [84]. Furthermore, inconsistencies in estimates across studies may reflect a dual role for antibodies to VAR2CSA, whereby antibodies contribute to protective immunity via blocking of pRBC adhesion to CSA, but also contribute to local pathology by activating inflammatory monocytes and macrophages in the placenta [85, 86]. In addition, differences in the methodology employed to measure antibody responses may have contributed to heterogeneity in estimates. Furthermore, due to the limited availability of stratified data, we only presented estimates for women sub-grouped into primigravidae and secundigravidae/multigravidae groups. In some earlier studies, antibodies to pregnancy-specific *P. falciparum* antigens were associated with improved pregnancy outcomes in secundigravidae only [56], or in women with chronic pregnancy-associated malaria infections [61]. Therefore, further studies examining antibody responses in specific parity and clinical groups may be warranted.

Due to the breadth of study designs and antigens examined, in most cases estimates for the association between a specific antibody response and clinical outcome across different populations could not be pooled. Importantly, estimates for the association between antibody responses and PAM outcomes derived from a single population may not be generalizable to other malaria-endemic areas. We identified a lack of cohort studies examining associations between antibody responses and prospective risk of malaria in pregnancy. The majority of the included estimates were derived from cross-sectional analyses, usually at delivery, thus limiting the ability to establish a causal relationship between antibody responses and PAM outcomes. Moreover, measurements of immunity inferred by antibody levels at a single time point can be misleading as antibody production is highly dynamic [39, 51, 57, 87]. Importantly, many of the studies were not originally designed to detect differences in the risk of placental infection and associated outcomes between antibody responders and non-responders and were therefore not statistically powered to detect such associations.

Study populations varied with respect to the proportion of primigravidae versus multigravidae, the percentage of women receiving intermittent preventive treatment in pregnancy (IPTp), the prevalence of HIV and other infectious diseases, the transmission intensity of the study site, and the malaria exposure history of the women. Both parity and transmission intensity can influence the kinetics of anti-VAR2CSA antibody responses during pregnancy [35, 38, 39, 73]. Moreover, the transmission intensity may affect the relationship between antibody responses and PAM [74, 75] as women living in areas of stable/intense malaria transmission may develop a broader repertoire of functional antibody responses to pregnancy-associated *P. falciparum* earlier in pregnancy, than women facing low/seasonal transmission [74]. Because antibody responses to the same antigen were rarely examined across multiple populations using comparable study designs, it was difficult to assess the effect of transmission intensity. Understanding the impact of transmission intensity on acquired immunity has implications for both serosurveillance and vaccine development.

## Conclusion

Overall, this systematic review found that pregnancy-specific *P. falciparum* antibody responses likely serve as markers of exposure to malaria in pregnancy, rather than correlates of protection. In order to objectively identify and prioritize antigens for vaccine development, it is important to demonstrate that a candidate antigen is a specific target of immune responses associated with protection from symptomatic disease in naturally exposed populations [88, 89]. Rational vaccine design and the development of immuno-serosurveillance tools would benefit from additional prospective cohort studies examining antibody responses early in pregnancy and at multiple time points throughout to a broad range of pregnancy-specific *P. falciparum* antigens across different epidemiological settings.

## Supplementary information

**Additional file 1.** Prisma 2009 Checklist.

**Additional file 2.** Full search strategy for Pubmed database.

**Additional file 3.** Data extraction form.

**Additional file 4.** Risk of bias assessment.

**Additional file 5.** Excluded studies.

**Additional file 6.** Details of parasites and recombinant antigens featured in review.

**Additional file 7.** Supplementary forest plots and results for additional outcomes.

## Data Availability

The dataset used and analysed during the current study are available from the corresponding author on reasonable request.
