# Supplementary material for: Pregnancy-specific malarial immunity and risk of malaria in pregnancy and adverse birth outcomes: a systematic review
Source: BMC Med. 2020 Jan 16;18:14. doi: 10.1186/s12916-019-1467-6 (PMC6964062; doi:10.1186/s12916-019-1467-6)
Supplement: Supplementary file 2 — Additional file 2. Full search strategy for Pubmed database. [file 12916_2019_1467_MOESM2_ESM.docx]

Additional file 2: Full search strategy for Pubmed database

| Database | Search string | Qualifiers / Filters | Results |
| --- | --- | --- | --- |
| Pubmed | ((((VAR2CSA OR VSA OR "variant surface antigen" OR PfEMP1 OR DBL OR "Duffy binding like"))) AND ((immune OR immunity OR antibod* OR IgG))) AND (( birth* OR preterm OR prematur* OR growth OR IUGR OR gestational OR SGA OR placenta* OR anaemia OR malaria OR plasmodium)) | Publication date: to 07-June-2019 | 583 |
