# Supplementary material for: Pregnancy-specific malarial immunity and risk of malaria in pregnancy and adverse birth outcomes: a systematic review
Source: BMC Med. 2020 Jan 16;18:14. doi: 10.1186/s12916-019-1467-6 (PMC6964062; doi:10.1186/s12916-019-1467-6)
Supplement: Supplementary file 3 — Additional file 3. Data extraction form. [file 12916_2019_1467_MOESM3_ESM.doc]

# **Author: Year:**

#

# **2 x 2 tables for categorical variables**

Outcome of interest:

Primigravidae

| Antigen^#^:  Antibody class or subclass: IgG | Outcome positive (n) | Outcome negative (n) |
| --- | --- | --- |
| Antibody positive (n) |  |  |
| Antibody negative (n) |  |  |

**Secund**igravidae

| Antigen^#^:  Antibody class or subclass: IgG | Outcome positive (n) | Outcome negative (n) |
| --- | --- | --- |
| Antibody positive (n) |  |  |
| Antibody negative (n) |  |  |

**Mult**igravidae

| Antigen^#^:  Antibody class or subclass: IgG | Outcome positive (n) | Outcome negative (n) |
| --- | --- | --- |
| Antibody positive (n) |  |  |
| Antibody negative (n) |  |  |

^#^Use separate 2x2 tables for different antigens ^#^Use separate 2x2 tables for different antigens

# **Author: Year:**

| **Antigen**  **(allele)** | **Antibody** | **Sero-prevalence** | **Antibody data*** | **Outcome** | **Measure of Association**** | **Estimate** | **Lower 95% CI** | **Upper 95% CI** | **Adjustments** | **Notes** |
| --- | --- | --- | --- | --- | --- | --- | --- | --- | --- | --- |
| *Eg.CS2 infected RBC* | *IgG* | *25%* | *Pos/neg* | *Placental infection* | *OR* | *0.75* | *0.55* | *0.95* | *Age* |  |
|  |  |  |  |  |  |  |  |  |  |  |
|  |  |  |  |  |  |  |  |  |  |  |
|  |  |  |  |  |  |  |  |  |  |  |
|  |  |  |  |  |  |  |  |  |  |  |
|  |  |  |  |  |  |  |  |  |  |  |
|  |  |  |  |  |  |  |  |  |  |  |
|  |  |  |  |  |  |  |  |  |  |  |
|  |  |  |  |  |  |  |  |  |  |  |
|  |  |  |  |  |  |  |  |  |  |  |
|  |  |  |  |  |  |  |  |  |  |  |
|  |  |  |  |  |  |  |  |  |  |  |
|  |  |  |  |  |  |  |  |  |  |  |
|  |  |  |  |  |  |  |  |  |  |  |
|  |  |  |  |  |  |  |  |  |  |  |

^#^Use separate lines for different antibodies (eg IgM, IgG, IgG1 etc) and antigens * i.e. positive/negative, high/medium/low, log base 2. For continuous antibody data please state the mean (SD) and for categorical data please state how these were defined.

** e.g. OR, RR, HR.
