# Supplementary material for: Pregnancy-specific malarial immunity and risk of malaria in pregnancy and adverse birth outcomes: a systematic review
Source: BMC Med. 2020 Jan 16;18:14. doi: 10.1186/s12916-019-1467-6 (PMC6964062; doi:10.1186/s12916-019-1467-6)
Supplement: Supplementary file 4 — Additional file 4. Risk of bias assessment. [file 12916_2019_1467_MOESM4_ESM.docx]

**Additional file 4: Risk of bias assessment**

Risk of bias was assessed for each study using the Risk of Bias in Non-randomized Studies – of Interventions (ROBINS-I) assessment tool. The ROBINS-I tool enables assessment of bias with seven domains: 1) confounding; 2) selection of participants; 3) classification of ‘interventions’ (i.e. exposures); 4) deviations from intended ‘interventions’; 5) missing data; 6) measurement of outcomes; 7) and selection of reported results. Our risk of bias assessment pertains to the association between pregnancy specific *P. falciparum* antibody responses and pregnancy and birth outcomes derived from the study, rather than the study itself. Many studies were classified as having a ‘moderate’ or ‘serious’ risk of bias due to confounding for reasons that were usually not related to the quality of the study, including:

- Inclusion of studies in which the primary research question was not the association between *P. falciparum* antibodies and pregnancy associated malaria
- Inclusion of studies in which very few women had pregnancy associated malaria
- Authors responding to our request for cross-tabulated (i.e. unadjusted) data for antibody responses and malaria in pregnancy


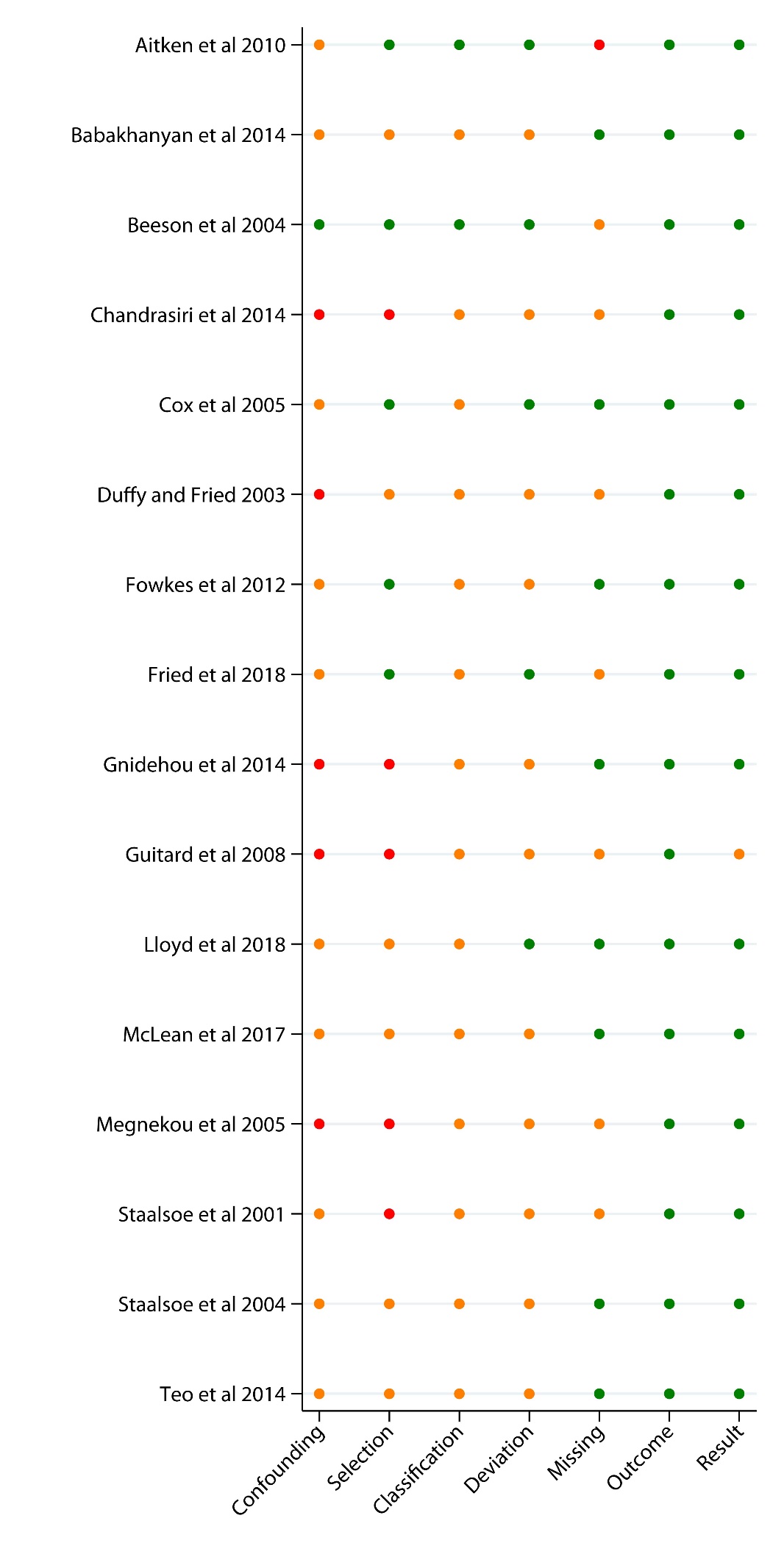


Figure S1. Risk of bias assessment. (Black – critical risk); red – serious risk; orange – moderate risk; green – low risk; (hollow circle – no information).
