# Supplementary material for: Pregnancy-specific malarial immunity and risk of malaria in pregnancy and adverse birth outcomes: a systematic review
Source: BMC Med. 2020 Jan 16;18:14. doi: 10.1186/s12916-019-1467-6 (PMC6964062; doi:10.1186/s12916-019-1467-6)
Supplement: Supplementary file 5 — Additional file 5. Excluded studies. [file 12916_2019_1467_MOESM5_ESM.docx]

**Additional file 5: Details of excluded studies**


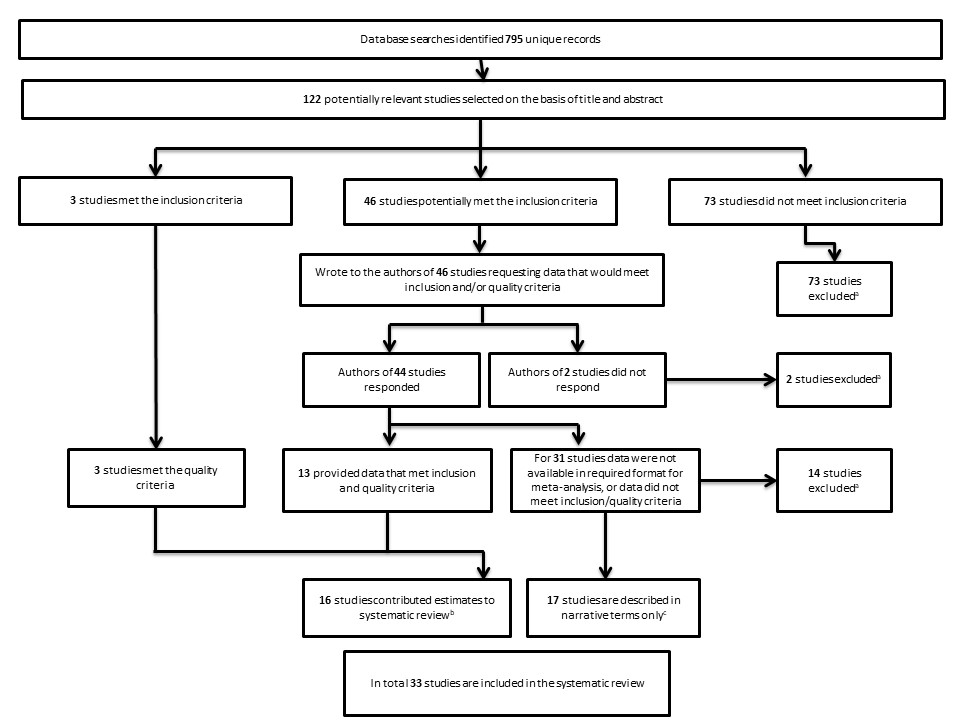


*Figure 1 from manuscript*

A total of 122 potentially relevant studies were selected on the basis of title and abstract.

The full texts of these studies were examined to determine whether the studies met the inclusion criteria outlined in the manuscript:

- 3 studies met the inclusion and quality criteria enabling inclusion in the review [1-3].
- 73 studies did not meet the inclusion criteria, with specific reasons for exclusion provided below:
  - 35 studies were excluded because pregnant women’s antibody responses were either not determined, only total serum/plasma immunoglobulin levels were determined, or responses to non-pregnancy specific antigens or ill-defined antigens were determined [4-38].
  - 29 studies were excluded because no outcomes of interest were included in study [39-67]
  - 2 studies were excluded because the relationship between antibody responses and outcomes of interest were not analysed at all [68, 69].
  - 2 studies were excluded because infants rather than pregnant women were recruited into the study [70, 71].
  - 1 study was excluded because relevant estimates were included in another study with larger sample size or a more complete/available dataset [72].
  - 1 study was excluded because women were excluded from study if they were parasitaemic by microscopy at enrolment [73].
  - 1 study was excluded because antibody responses were measured postpartum, rather than during pregnancy [74].
  - 2 studies were excluded because they were not population-based studies [75, 76].
- 46 studies potentially met the inclusion criteria but either data needed to be re-analysed to enable inclusion, or more information about the study was necessary to determine whether it ought to be included. We therefore wrote to the authors of 46 studies requesting data that would meet inclusion and/or quality criteria
  - The authors of 2 studies did not respond and these studies were excluded due to a lack of information about the study and/or data to enable inclusion [77, 78].
  - The authors of 13 studies responded and were able to provide data/information to enable data from their studies to be included in a format enabling inclusion in meta-analysis [79-91]
  - For 31 studies, the authors responded and reported that data was not available in required format for meta-analysis, or data did not meet inclusion/quality criteria:
    - For 17 studies, authors reported that data was not available in required format for meta-analysis, but enough information was included in the publication to enable inclusion in narrative (descriptive) terms (table 2) [92-108]
    - 14 studies were excluded following responses from authors:
      - The authors of 3 studies responded but their studies were ultimately excluded because the relevant estimates were available from other publications investigating the same study population [109-111].
      - The authors of 11 studies reported that the requested data/information was not available, so these studies were excluded [112-122]

References

1. Beeson JG, Mann EJ, Elliott SR, Lema VM, Tadesse E, Molyneux ME, Brown GV, Rogerson SJ: **Antibodies to variant surface antigens of Plasmodium falciparum - Infected erythrocytes and adhesion inhibitory antibodies are associated with placental malaria and have overlapping and distinct targets**. *Journal of Infectious Diseases* 2004, **189**(3):540-551.

2. Chandrasiri UP, Randall LM, Saad AA, Bashir AM, Rogerson SJ, Adam I: **Low antibody levels to pregnancy-specific malaria antigens and heightened cytokine responses associated with severe malaria in pregnancy**. *J Infect Dis* 2014, **209**(9):1408-1417.

3. Lloyd YM, Fang R, Bobbili N, Vanda K, Ngati E, Sanchez-Quintero MJ, Salanti A, Chen JJ, Leke RGF, Taylor DW: **Association of Antibodies to VAR2CSA and Merozoite Antigens with Pregnancy Outcomes in Women Living in Yaounde, Cameroon**. *Infect Immun* 2018, **86**(9).

4. Achidi EA, Perlmann H, Salimonu LS, Asuzu MC, Perlmann P, Berzins K: **Antibodies to Pf155/RESA and circumsporozoite protein of Plasmodium falciparum in paired maternal-cord sera from Nigeria**. *Parasite Immunol* 1995, **17**(10):535-540.

5. Achidi EA, Anchang JK, Minang JT, Ahmadou MJ, Troye-Blomberg M: **Studies on Plasmodium falciparum isotypic antibodies and numbers of IL-4 and IFN-gamma secreting cells in paired maternal cord blood from South West Cameroon**. *Int J Infect Dis* 2005, **9**(3):159-169.

6. Akanmori BD, Afari EA, Sakatoku H, Nkrumah FK: **A LONGITUDINAL-STUDY OF MALARIA INFECTION, MORBIDITY AND ANTIBODY-TITERS IN INFANTS OF A RURAL-COMMUNITY IN GHANA**. *Transactions of the Royal Society of Tropical Medicine and Hygiene* 1995, **89**(5):560-561.

7. Akim NI, Urassa H, Drakeley CJ, Sauerwein RW, Kitua AY: **Immunity to the sexual stages of Plasmodium falciparum in mothers, neonates and infants subject to intense and perennial malarial transmission**. *Ann Trop Med Parasitol* 2002, **96**(7):735-737.

8. Akum AE, Minang JT, Kuoh AJ, Ahmadou MJ, Troye-Blomberg M: **Plasmodium falciparum inhibitory capacities of paired maternal-cord sera from south-west province, Cameroon**. *J Trop Pediatr* 2005, **51**(3):182-190.

9. Astagneau P, Steketee RW, Wirima JJ, Khoromana CO, Millet P: **Antibodies to ring-infected erythrocyte surface antigen (Pf155/RESA) protect against P. falciparum parasitemia in highly exposed multigravidas women in Malawi**. *Acta Trop* 1994, **57**(4):317-325.

10. Chizzolini C, Trottein F, Bernard FX, Kaufmann MH: **ISOTYPIC ANALYSIS, ANTIGEN-SPECIFICITY, AND INHIBITORY FUNCTION OF MATERNALLY TRANSMITTED PLASMODIUM-FALCIPARUM-SPECIFIC ANTIBODIES IN GABONESE NEWBORNS**. *American Journal of Tropical Medicine and Hygiene* 1991, **45**(1):57-64.

11. Deloron P, Dubois B, Le Hesran JY, Riche D, Fievet N, Cornet M, Ringwald P, Cot M: **Isotypic analysis of maternally transmitted Plasmodium falciparum-specific antibodies in Cameroon, and relationship with risk of P. falciparum infection**. *Clin Exp Immunol* 1997, **110**(2):212-218.

12. Dent A, Malhotra I, Mungai P, Muchiri E, Crabb BS, Kazura JW, King CL: **Prenatal malaria immune experience affects acquisition of Plasmodium falciparum merozoite surface protein-1 invasion inhibitory antibodies during infancy**. *Journal of Immunology* 2006, **177**(10):7139-7145.

13. Dent AE, Malhotra I, Wang X, Babineau D, Yeo KT, Anderson T, Kimmel RJ, Angov E, Lanar DE, Narum D *et al*: **Contrasting Patterns of Serologic and Functional Antibody Dynamics to Plasmodium falciparum Antigens in a Kenyan Birth Cohort**. *Clin Vaccine Immunol* 2016, **23**(2):104-116.

14. Egwunyenga OA, Ajayi JA, Duhlinska-Popova DD: **Transplacental passage of Plasmodium falciparum and seroevaluation of newborns in northern Nigeria**. *Southeast Asian J Trop Med Public Health* 1997, **28**(4):741-745.

15. Ezeoke AC, Ibanga NJ, Braide EI: **Congenital malaria at University of Calabar Teaching Hospital with reference to haemoglobin and immunoglobulin**. *Cent Afr J Med* 1985, **31**(12):241-247.

16. Fievet N, Cot M, Ringwald P, Bickii J, Dubois B, LeHesran JY, Migot F, Deloron P: **Immune response to Plasmodium falciparum antigens in Cameroonian primigravidae: Evolution after delivery and during second pregnancy**. *Clinical and Experimental Immunology* 1997, **107**(3):462-467.

17. Garin YJ, Blot P, Walter P, Pinon JM, Vernes A: **[Malarial infection of the placenta. Parasitologic, clinical and immunologic aspects]**. *Arch Fr Pediatr* 1985, **42 Suppl 2**:917-920.

18. King CL, Malhotra I, Wamachi A, Kioko J, Mungai P, Wahab SA, Koech D, Zimmerman P, Ouma J, Kazura JW: **Acquired immune responses to Plasmodium falciparum merozoite surface protein-1 in the human fetus**. *J Immunol* 2002, **168**(1):356-364.

19. Kirch AK, Agossou A, Banla M, Hoffmann WH, Schulz-Key H, Soboslay PT: **Parasite-specific antibody and cytokine profiles in newborns from Plasmodium falciparum and Entamoeba histolytica/dispar-infected mothers**. *Pediatric Allergy and Immunology* 2004, **15**(2):133-141.

20. Maubert B, Fievet N, Tami G, Boudin C, Deloron P: **Cytoadherence of Plasmodium falciparum-infected erythrocytes in the human placenta**. *Parasite Immunol* 2000, **22**(4):191-199.

21. McGregor IA, Wilson RJ: **Precipitating antibodies and immunoglobulins in P. falciparum infections in The Gambia, West Africa**. *Trans R Soc Trop Med Hyg* 1971, **65**(2):136-151.

22. Mvondo JL, James MA, Sulzer AJ, Campbell CC: **Malaria and pregnancy in Cameroonian women. Naturally acquired antibody responses to asexual blood-stage antigens and the circumsporozoite protein of Plasmodium falciparum**. *Transactions of the Royal Society of Tropical Medicine and Hygiene* 1992, **86**(5):486-490.

23. Nasr A, Hamid O, Al-Ghamdi A, Allam G: **Anti-malarial IgG subclasses pattern and Fc gamma RIIa (CD32) polymorphism among pregnancy-associated malaria in semi-immune Saudi women**. *Malaria Journal* 2013, **12**.

24. Ned RM, Price AE, Crawford SB, Ayisi JG, van Eijk AM, Otieno JA, Nahlen BL, Steketee RW, Slutsker L, Shi YP *et al*: **Effect of placental malaria and HIV infection on the antibody responses to Plasmodium falciparum in infants**. *J Infect Dis* 2008, **198**(11):1609-1619.

25. Onyenekwe CC, Arinola OG, Salimonu LS: **Detection of Plasmodium falciparum-IgG and incidence of asymptomatic malaria in pregnant women in Nigeria**. *Indian J Malariol* 2002, **39**(1-2):39-42.

26. Onyenekwe CC, Arinola OG, Meludu SC, Salimonu LS, Adewale IF, Obisesan AK: **Malaria parasitaemia and plasmodium falciparum specific-IgG in maternal peripheral, placental and cord circulaton**. *J Vector Borne Dis* 2004, **41**(3-4):72-75.

27. Owalla TJ, Palacpac NMQ, Shirai H, Horii T, Egwang TG: **Association of naturally acquired IgG antibodies against Plasmodium falciparum serine repeat antigen-5 with reduced placental parasitemia and normal birth weight in pregnant Ugandan women: A pilot study**. *Parasitology International* 2013, **62**(3):237-239.

28. Rasheed FN, Bulmer JN, De Francisco A, Jawla MF, Jakobsen PH, Jepson A, Greenwood BM: **Relationships between maternal malaria and malarial immune responses in mothers and neonates**. *Parasite Immunol* 1995, **17**(1):1-10.

29. Riley EM, Wagner GE, Ofori MF, Wheeler JG, Akanmori BD, Tetteh K, McGuinness D, Bennett S, Nkrumah FK, Anders RF *et al*: **Lack of association between maternal antibody and protection of African infants from malaria infection**. *Infect Immun* 2000, **68**(10):5856-5863.

30. Schleiermacher D, Rogier C, Spiegel A, Tall A, Trape JF, Mercerau-Puijalon O: **Increased multiplicity of Plasmodium falciparum infections and skewed distribution of individual MSP1 and MSP2 alleles during pregnancy in Ndiop, a Senegalese village with seasonal, mesoendemic malaria**. *American Journal of Tropical Medicine and Hygiene* 2001, **64**(5-6):303-309.

31. Serghides L, Patel SN, Ayi K, Kain KC: **Placental chondroitin sulfate A - Binding malarial isolates evade innate phagocytic clearance**. *Journal of Infectious Diseases* 2006, **194**(1):133-139.

32. Suguitan AL, Gowda DC, Fouda G, Thuita L, Zhou AN, Djokam R, Metenou S, Leke RGF, Taylor DW: **Lack of an association between antibodies to Plasmodium falciparum glycosylphosphatidylinositols and malaria-associated placental changes in cameroonian women with preterm and full-term deliveries**. *Infection and Immunity* 2004, **72**(9):5267-5273.

33. Tena-Tomas C, Bouyou-Akotet MK, Kendjo E, Kombila M, Kremsner PG, Kun JFJ: **Prenatal immune responses to Plasmodium falciparum erythrocyte membrane protein 1 DBL-alpha domain in Gabon**. *Parasitology Research* 2007, **101**(4):1045-1050.

34. Uneke C, Sagay A, Nwanna G, Jagun B, Nwakpu K: **Effects of maternal malaria and anaemia acquired during pregnancy on neonatal birth weight**. *Journal of Health and Visual Sciences* 2004, **6**(3).

35. Walker PGT, Griffin JT, Cairns M, Rogerson SJ, van Eijk AM, ter Kuile F, Ghani AC: **A model of parity-dependent immunity to placental malaria**. *Nature Communications* 2013, **4**.

36. Williams AI, McFarlane H: **Immunoglobulin levels, malarial antibody titres and placental parasitaemia in Nigerian mothers and neonates**. *Afr J Med Sci* 1970, **1**(4):369-376.

37. Xi GL, Leke RGF, Thuita LW, Zhou AN, Leke RJI, Mbu R, Taylor DW: **Congenital exposure to Plasmodium falciparum antigens: Prevalence and antigenic specificity of in utero-produced antimalarial immunoglobulin M antibodies**. *Infection and Immunity* 2003, **71**(3):1242-1246.

38. Zhou A, Megnekou R, Leke R, Fogako J, Metenou S, Trock B, Taylor DW, Leke RF: **Prevalence of Plasmodium falciparum infection in pregnant Cameroonian women**. *Am J Trop Med Hyg* 2002, **67**(6):566-570.

39. Beeson JG, Brown GV, Molyneux ME, Mhango C, Dzinjalamala F, Rogerson SJ: **Plasmodium falciparum isolates from infected pregnant women and children are associated with distinct adhesive and antigenic properties**. *Journal of Infectious Diseases* 1999, **180**(2):464-472.

40. Beeson JG, Mann EJ, Byrne TJ, Caragounis A, Elliott SR, Brown GV, Rogerson SJ: **Antigenic differences and conservation among placental Plasmodium falciparum-infected erythrocytes and acquisition of variant-specific and cross-reactive antibodies**. *Journal of Infectious Diseases* 2006, **193**(5):721-730.

41. Dembo EG, Mwapasa V, Montgomery J, Craig AG, Porter KA, Meshnick SR, Molyneux ME, Rogerson SJ: **Impact of human immunodeficiency virus infection in pregnant women on variant-specific immunity to malaria**. *Clin Vaccine Immunol* 2008, **15**(4):617-621.

42. Doritchamou JY, Herrera R, Aebig JA, Morrison R, Nguyen V, Reiter K, Shimp RL, MacDonald NJ, Narum DL, Fried M *et al*: **VAR2CSA Domain-Specific Analysis of Naturally Acquired Functional Antibodies to Plasmodium falciparum Placental Malaria**. *J Infect Dis* 2016, **214**(4):577-586.

43. Gnidehou S, Jessen L, Gangnard S, Ermont C, Triqui C, Quiviger M, Guitard J, Lund O, Deloron P, Ndam NT: **Insight into Antigenic Diversity of VAR2CSA-DBL5 epsilon Domain from Multiple Plasmodium falciparum Placental Isolates**. *Plos One* 2010, **5**(10).

44. Haase RN, Megnekou R, Lundquist M, Ofori MF, Hviid L, Staalsoe T: **Plasmodium falciparum parasites expressing pregnancy-specific variant surface antigens adhere strongly to the choriocarcinorna cell line BeWo**. *Infection and Immunity* 2006, **74**(5):3035-3038.

45. Hogh B, Marbiah NT, Burghaus PA, Andersen PK: **Relationship between maternally derived anti-Plasmodium falciparum antibodies and risk of infection and disease in infants living in an area of Liberia, west Africa, in which malaria is highly endemic**. *Infect Immun* 1995, **63**(10):4034-4038.

46. Jensen ATR, Zornig HD, Buhmann C, Salanti A, Koram KA, Riley EM, Theander TG, Hviid L, Staalsoe T: **Lack of gender-specific antibody recognition of products from domains of a var gene implicated in pregnancy-associated Plasmodium falciparum malaria**. *Infection and Immunity* 2003, **71**(7):4193-4196.

47. Khattab A, Kun J, Deloron P, Kremsner PG, Klinkert MQ: **Variants of Plasmodium falciparum erythrocyte membrane protein 1 expressed by different placental parasites are closely related and adhere to chondroitin sulfate A**. *J Infect Dis* 2001, **183**(7):1165-1169.

48. Khattab A, Kremsner PG, Klinkert MQ: **Common surface-antigen var genes of limited diversity expressed by Plasmodium falciparum placental isolates separated by time and space**. *Journal of Infectious Diseases* 2003, **187**(3):477-483.

49. Le Hesran JY, Cot M, Personne P, Fievet N, Dubois B, Beyeme M, Boudin C, Deloron P: **Maternal placental infection with Plasmodium falciparum and malaria morbidity during the first 2 years of life**. *Am J Epidemiol* 1997, **146**(10):826-831.

50. Le Port A, Cottrell G, Chandre F, Cot M, Massougbodji A, Garcia A: **Importance of adequate local spatiotemporal transmission measures in malaria cohort studies: application to the relation between placental malaria and first malaria infection in infants**. *Am J Epidemiol* 2013, **178**(1):136-143.

51. Magistrado P, Salanti A, Ndam NGT, Mwakalinga SB, Resende M, Dahlback M, Hviid L, Lusingu J, Theander TG, Nielsen MA: **VAR2CSA expression on the surface of placenta-derived Plasmodium falciparum - Infected erythrocytes**. *Journal of Infectious Diseases* 2008, **198**(7):1071-1074.

52. Magistrado PA, Minja D, Doritchamou J, Ndam NT, John D, Schmiegelow C, Massougbodji A, Dahlback M, Ditlev SB, Pinto VV *et al*: **High efficacy of anti DBL4 epsilon-VAR2CSA antibodies in inhibition of CSA-binding Plasmodium falciparum-infected erythrocytes from pregnant women**. *Vaccine* 2011, **29**(3):437-443.

53. Mann EJ, Rogerson SJ, Beeson JG: **An alternative agglutination assay to measure antibodies to variant surface antigens of Plasmodium falciparum-infected erythrocytes**. *Trans R Soc Trop Med Hyg* 2003, **97**(6):717-719.

54. May K, Grube M, Malhotra I, Long CA, Singh S, Mandaliya K, Siegmund W, Fusch C, Schneider H, King CL: **Antibody-Dependent Transplacental Transfer of Malaria Blood-Stage Antigen Using a Human Ex Vivo Placental Perfusion Model**. *Plos One* 2009, **4**(11).

55. Nambei WS, Goumbala M, Spiegel A, Dieye A, Perraut R, Garraud O: **Imbalanced distribution of IgM and IgG antibodies against Plasmodium falciparum antigens and merozoite surface protein-1 (MSP1) in pregnancy**. *Immunology Letters* 1998, **61**(2-3):197-199.

56. Nunes MC, Sterkers Y, Gamain B, Scherf A: **Investigation of host factors possibly enhancing the emergence of the chondroitin sulfate A-binding phenotype in Plasmodium falciparum**. *Microbes and Infection* 2008, **10**(8):928-932.

57. Ricke CH, Staalsoe T, Koram K, Akanmori BD, Riley EM, Theander TG, Hviid L: **Plasma antibodies from malaria-exposed pregnant women recognize variant surface antigens on Plasmodium falciparum-infected erythrocytes in a parity-dependent manner and block parasite adhesion to chondroitin sulfate A**. *Journal of Immunology* 2000, **165**(6):3309-3316.

58. Rovira-Vallbona E, Dobano C, Bardaji A, Cistero P, Romagosa C, Serra-Casas E, Quinto L, Bassat Q, Sigauque B, Alonso PL *et al*: **Transcription of var Genes Other Than var2csa in Plasmodium falciparum Parasites Infecting Mozambican Pregnant Women**. *Journal of Infectious Diseases* 2011, **204**(1):27-35.

59. Rovira-Vallbona E, Monteiro I, Bardaji A, Serra-Casas E, Neafsey DE, Quelhas D, Valim C, Alonso P, Dobano C, Ordi J *et al*: **VAR2CSA Signatures of High Plasmodium falciparum Parasitemia in the Placenta**. *Plos One* 2013, **8**(7).

60. Saveria T, Oleinikov AV, Wiliamson K, Chaturvedi R, Lograsso J, Keitany GJ, Fried M, Duffy P: **Antibodies to Escherichia coli-Expressed C-Terminal Domains of Plasmodium falciparum Variant Surface Antigen 2-Chondroitin Sulfate A (VAR2CSA) Inhibit Binding of CSA-Adherent Parasites to Placental Tissue**. *Infection and Immunity* 2013, **81**(4):1031-1039.

61. Singh K, Gitti RK, Diouf A, Zhou H, Gowda DC, Miura K, Ostazeski SA, Fairhurst RM, Garboczi DN, Long CA: **Subdomain 3 of Plasmodium falciparum VAR2CSA DBL3x Is Identified as a Minimal Chondroitin Sulfate A-binding Region**. *Journal of Biological Chemistry* 2010, **285**(32):24855-24862.

62. Staalsoe T, Shulman CE, Dorman EK, Kawuondo K, Marsh K, Hviid L: **Intermittent preventive sulfadoxine-pyrimethamine treatment of primigravidae reduces levels of plasma immunoglobulin G, which protects against pregnancy-associated Plasmodium falciparum malaria**. *Infection and Immunity* 2004, **72**(9):5027-5030.

63. Ndam NGT, Fievet N, Bertin G, Cottrell G, Gaye A, Deloron P: **Variable adhesion abilities and overlapping antigenic properties in placental Plasmodium falciparum isolates**. *Journal of Infectious Diseases* 2004, **190**(11):2001-2009.

64. Ndam NGT, Salanti A, Bertin G, Dahlback M, Fievet N, Turner L, Gaye A, Theander T, Deloron P: **High level of var2csa transcription by Plasmodium falciparum isolated from the placenta**. *Journal of Infectious Diseases* 2005, **192**(2):331-335.

65. Yang JC, Blanton RE, King CL, Fujioka H, Aikawa M, Sam-Yellowe TY: **Seroprevalence and specificity of human responses to the Plasmodium falciparum rhoptry protein Rhop-3 determined by using a C-terminal recombinant protein**. *Infect Immun* 1996, **64**(9):3584-3591.

66. Yosaatmadja F, Andrews KT, Duffy MF, Brown GV, Beeson JG, Rogerson SJ: **Characterization of VAR2CSA-deficient Plasmodium falciparum-infected erythrocytes selected for adhesion to the BeWo placental cell line**. *Malaria Journal* 2008, **7**.

67. Zhou Z, Xiao L, Branch OH, Kariuki S, Nahlen BL, Lal AA: **Antibody responses to repetitive epitopes of the circumsporozoite protein, liver stage antigen-1, and merozoite surface protein-2 in infants residing in a Plasmodium falciparum-hyperendemic area of western Kenya. XIII. Asembo Bay Cohort Project**. *Am J Trop Med Hyg* 2002, **66**(1):7-12.

68. Benet A, Khong TY, Ura A, Samen R, Lorry K, Mellombo M, Tavul L, Baea K, Rogerson SJ, Cortes A: **Placental malaria in women with South-East Asian ovalocytosis**. *American Journal of Tropical Medicine and Hygiene* 2006, **75**(4):597-604.

69. Fonseca AM, Quinto L, Jimenez A, Gonzalez R, Bardaji A, Maculuve S, Dobano C, Ruperez M, Vala A, Aponte JJ *et al*: **Multiplexing detection of IgG against Plasmodium falciparum pregnancy-specific antigens**. *PLoS One* 2017, **12**(7):e0181150.

70. Branch OH, Udhayakumar V, Hightower AW, Oloo AJ, Hawley WA, Nahlen BL, Bloland PB, Kaslow DC, Lal AA: **A longitudinal investigation of IgG and IgM antibody responses to the merozoite surface protein-1 19-kiloDalton domain of Plasmodium falciparum in pregnant women and infants: associations with febrile illness, parasitemia, and anemia**. *Am J Trop Med Hyg* 1998, **58**(2):211-219.

71. Branch OH, Oloo AJ, Nahlen BL, Kaslow D, Lal AA: **Anti-merozoite surface protein-1 19-kDa IgG in mother-infant pairs naturally exposed to Plasmodium falciparum: subclass analysis with age, exposure to asexual parasitemia, and protection against malaria. V. The Asembo Bay Cohort Project**. *J Infect Dis* 2000, **181**(5):1746-1752.

72. Elliott SR, Duffy MF, Byrne TJ, Beeson JG, Mann EJ, Wilson DW, Rogerson SJ, Brown GV: **Cross-reactive surface epitopes on chondroitin sulfate A-adherent Plasmodium falciparum-infected erythrocytes are associated with transcription of var2csa**. *Infection and Immunity* 2005, **73**(5):2848-2856.

73. Gavina K, Gnidehou S, Arango E, Hamel-Martineau C, Mitran C, Agudelo O, Lopez C, Karidio A, Banman S, Carmona-Fonseca J *et al*: **Clinical Outcomes of Submicroscopic Infections and Correlates of Protection of VAR2CSA Antibodies in a Longitudinal Study of Pregnant Women in Colombia**. *Infect Immun* 2018, **86**(4).

74. Keen J, Serghides L, Ayi K, Patel SN, Ayisi J, van Eijk A, Steketee R, Udhayakumar V, Kain KC: **HIV impairs opsonic phagocytic clearance of pregnancy-associated malaria parasites**. *PLoS Med* 2007, **4**(5):e181.

75. Nielsen MA, Pinto VV, Resende M, Dahlback M, Ditlev SB, Theander TG, Salanti A: **Induction of Adhesion-Inhibitory Antibodies against Placental Plasmodium falciparum Parasites by Using Single Domains of VAR2CSA**. *Infection and Immunity* 2009, **77**(6):2482-2487.

76. Nielsen MA, Salanti A: **High-Throughput Testing of Antibody-Dependent Binding Inhibition of Placental Malaria Parasites**. *Methods Mol Biol* 2015, **1325**:241-253.

77. Brustoski K, Kramer M, Moller U, Kremsner PG, Luty AJF: **Neonatal and maternal immunological responses to conserved epitopes within the DBL-gamma 3 chondroitin sulfate A-binding domain of Plasmodium falciparum erythrocyte membrane protein 1**. *Infection and Immunity* 2005, **73**(12):7988-7995.

78. Dechavanne S, Srivastava A, Gangnard S, Nunes-Silva S, Dechavanne C, Fievet N, Deloron P, Chene A, Gamain B: **Parity-dependent recognition of DBL1X-3X suggests an important role of the VAR2CSA high-affinity CSA-binding region in the development of the humoral response against placental malaria**. *Infect Immun* 2015, **83**(6):2466-2474.

79. Aitken EH, Mbewe B, Luntamo M, Maleta K, Kulmala T, Friso MJ, Fowkes FJI, Beeson JG, Ashorn P, Rogerson SJ: **Antibodies to Chondroitin Sulfate A- Binding Infected Erythrocytes: Dynamics and Protection during Pregnancy in Women Receiving Intermittent Preventive Treatment**. *Journal of Infectious Diseases* 2010, **201**(9):1316-1325.

80. Babakhanyan A, Leke RG, Salanti A, Bobbili N, Gwanmesia P, Leke RJ, Quakyi IA, Chen JJ, Taylor DW: **The antibody response of pregnant Cameroonian women to VAR2CSA ID1-ID2a, a small recombinant protein containing the CSA-binding site**. *PLoS One* 2014, **9**(2):e88173.

81. Duffy PE, Fried M: **Antibodies that inhibit Plasmodium falciparum adhesion to chondroitin sulfate A are associated with increased birth weight and the gestational age of Newborns**. *Infection and Immunity* 2003, **71**(11):6620-6623.

82. Fowkes FJ, McGready R, Cross NJ, Hommel M, Simpson JA, Elliott SR, Richards JS, Lackovic K, Viladpai-Nguen J, Narum D *et al*: **New insights into acquisition, boosting, and longevity of immunity to malaria in pregnant women**. *J Infect Dis* 2012, **206**(10):1612-1621.

83. Fried M, Kurtis JD, Swihart B, Morrison R, Pond-Tor S, Barry A, Sidibe Y, Keita S, Mahamar A, Andemel N *et al*: **Antibody levels to recombinant VAR2CSA domains vary with Plasmodium falciparum parasitaemia, gestational age, and gravidity, but do not predict pregnancy outcomes**. *Malar J* 2018, **17**(1):106.

84. Guitard J, Cottrell G, Magnouha NM, Salanti A, Li T, Sow S, Deloron P, Ndam NT: **Differential evolution of anti-VAR2CSA-IgG3 in primigravidae and multigravidae pregnant women infected by Plasmodium falciparum**. *Malaria Journal* 2008, **7**.

85. McLean ARD, Stanisic D, McGready R, Chotivanich K, Clapham C, Baiwog F, Pimanpanarak M, Siba P, Mueller I, King CL *et al*: **P. falciparum infection and maternofetal antibody transfer in malaria-endemic settings of varying transmission**. *PLoS One* 2017, **12**(10):e0186577.

86. Megnekou R, Staalsoe T, Taylor DW, Leke R, Hviid L: **Effects of pregnancy and intensity of Plasmodium falciparum transmission on immunoglobulin G subclass responses to variant surface antigens**. *Infection and Immunity* 2005, **73**(7):4112-4118.

87. Staalsoe T, Megnekou R, Fievet N, Ricke CH, Zornig HD, Leke R, Taylor DW, Deloron P, Hviid L: **Acquisition and decay of antibodies to pregnancy-associated variant antigens on the surface of Plasmodium falciparum-infected erythrocytes that protect against placental parasitemia**. *Journal of Infectious Diseases* 2001, **184**(5):618-626.

88. Staalsoe T, Shulman CE, Bulmer JN, Kawuondo K, Marsh K, Hviid L: **Variant surface antigen-specific IgG and protection against clinical consequences of pregnancy-associated Plasmodium falciparum malaria**. *Lancet* 2004, **363**(9405):283-289.

89. Teo A, Hasang W, Randall LM, Feng G, Bell L, Unger H, Langer C, Beeson JG, Siba PM, Mueller I *et al*: **Decreasing malaria prevalence and its potential consequences for immunity in pregnant women**. *J Infect Dis* 2014, **210**(9):1444-1455.

90. Cox SE, Staalsoe T, Arthur P, Bulmer JN, Hviid L, Yeboah-Antwi K, Kirkwood BR, Riley EM: **Rapid acquisition of isolate-specific antibodies to chondroitin sulfate A-adherent plasmodium falciparum isolates in Ghanaian primigravidae**. *Infect Immun* 2005, **73**(5):2841-2847.

91. Gnidehou S, Doritchamou J, Arango EM, Cabrera A, Arroyo MI, Kain KC, Ndam NT, Maestre A, Yanow SK: **Functional antibodies against VAR2CSA in nonpregnant populations from colombia exposed to Plasmodium falciparum and Plasmodium vivax**. *Infect Immun* 2014, **82**(6):2565-2573.

92. Ataide R, Hasang W, Wilson DW, Beeson JG, Mwapasa V, Molyneux ME, Meshnick SR, Rogerson SJ: **Using an Improved Phagocytosis Assay to Evaluate the Effect of HIV on Specific Antibodies to Pregnancy-Associated Malaria**. *Plos One* 2010, **5**(5).

93. Ataide R, Mwapasa V, Molyneux ME, Meshnick SR, Rogerson SJ: **Antibodies That Induce Phagocytosis of Malaria Infected Erythrocytes: Effect of HIV Infection and Correlation with Clinical Outcomes**. *Plos One* 2011, **6**(7).

94. Babakhanyan A, Fang R, Wey A, Salanti A, Sama G, Efundem C, Leke RJ, Chen JJ, Leke RG, Taylor DW: **Comparison of the specificity of antibodies to VAR2CSA in Cameroonian multigravidae with and without placental malaria: a retrospective case-control study**. *Malar J* 2015, **14**:480.

95. Chandrasiri UP, Fowkes FJ, Beeson JG, Richards JS, Kamiza S, Maleta K, Ashorn P, Rogerson SJ: **Association between malaria immunity and pregnancy outcomes among Malawian pregnant women receiving nutrient supplementation**. *Malar J* 2016, **15**(1):547.

96. Feng GQ, Aitken E, Yosaatmadja F, Kalilani L, Meshnick SR, Jaworowski A, Simpson JA, Rogerson SJ: **Antibodies to Variant Surface Antigens of Plasmodium falciparum-Infected Erythrocytes Are Associated with Protection from Treatment Failure and the Development of Anemia in Pregnancy**. *Journal of Infectious Diseases* 2009, **200**(2):299-306.

97. Hommel M, Elliott SR, Soma V, Kelly G, Fowkes FJI, Chesson JM, Duffy MF, Bockhorst J, Avril M, Mueller I *et al*: **Evaluation of the Antigenic Diversity of Placenta-Binding Plasmodium falciparum Variants and the Antibody Repertoire among Pregnant Women**. *Infection and Immunity* 2010, **78**(5):1963-1978.

98. Khattab A, Reinhardt C, Staalsoe T, Fievet N, Kremsner PG, Deloron P, Hviid L, Klinkert MQ: **Analysis of IgG with specificity for variant surface antigens expressed by placental Plasmodium falciparum isolates**. *Malaria Journal* 2004, **3**.

99. Mayor A, Rovira-Vallbona E, Machevo S, Bassat Q, Aguilar R, Quinto L, Jimenez A, Sigauque B, Dobano C, Kumar S *et al*: **Parity and Placental Infection Affect Antibody Responses against Plasmodium falciparum during Pregnancy**. *Infection and Immunity* 2011, **79**(4):1654-1659.

100. Mayor A, Kumar U, Bardaji A, Gupta P, Jimenez A, Hamad A, Sigauque B, Singh B, Quinto L, Kumar S *et al*: **Improved Pregnancy Outcomes in Women Exposed to Malaria With High Antibody Levels Against Plasmodium falciparum**. *Journal of Infectious Diseases* 2013, **207**(11):1664-1674.

101. O'Neil-Dunne I, Achur RN, Agbor-Enoh ST, Valiyaveettil M, Naik RS, Ockenhouse CF, Zhou AN, Megnekou R, Leke R, Taylor DW *et al*: **Gravidity-dependent production of antibodies that inhibit binding of Plasmodium falciparum-infected erythrocytes to placental chondroitin sulfate proteoglycan during pregnancy**. *Infection and Immunity* 2001, **69**(12):7487-7492.

102. Salanti A, Dahlback M, Turner L, Nielsen MA, Barfod L, Magistrado P, Jensen ATR, Lavstsen T, Ofori MF, Marsh K *et al*: **Evidence for the involvement of VAR2CSA in pregnancy-associated malaria**. *Journal of Experimental Medicine* 2004, **200**(9):1197-1203.

103. Siriwardhana C, Fang R, Salanti A, Leke RGF, Bobbili N, Taylor DW, Chen JJ: **Statistical prediction of immunity to placental malaria based on multi-assay antibody data for malarial antigens**. *Malar J* 2017, **16**(1):391.

104. Tuikue Ndam NG, Salanti A, Le-Hesran JY, Cottrell G, Fievet N, Turner L, Sow S, Dangou JM, Theander T, Deloron P: **Dynamics of anti-VAR2CSA immunoglobulin G response in a cohort of senegalese pregnant women**. *J Infect Dis* 2006, **193**(5):713-720.

105. Ndam NT, Denoeud-Ndam L, Doritchamou J, Viwami F, Salanti A, Nielsen MA, Fievet N, Massougbodji A, Luty AJ, Deloron P: **Protective Antibodies against Placental Malaria and Poor Outcomes during Pregnancy, Benin**. *Emerg Infect Dis* 2015, **21**(5):813-823.

106. Tutterrow YL, Avril M, Singh K, Long CA, Leke RJ, Sama G, Salanti A, Smith JD, Leke RGF, Taylor DW: **High Levels of Antibodies to Multiple Domains and Strains of VAR2CSA Correlate with the Absence of Placental Malaria in Cameroonian Women Living in an Area of High Plasmodium falciparum Transmission**. *Infection and Immunity* 2012, **80**(4):1479-1490.

107. Tutterrow YL, Salanti A, Avril M, Smith JD, Pagano IS, Ako S, Fogako J, Leke RG, Taylor DW: **High avidity antibodies to full-length VAR2CSA correlate with absence of placental malaria**. *Plos One* 2012, **7**(6):e40049.

108. Serra-Casas E, Menendez C, Bardaji A, Quinto L, Dobano C, Sigauque B, Jimenez A, Mandomando I, Chauhan VS, Chitnis CE *et al*: **The effect of intermittent preventive treatment during pregnancy on malarial antibodies depends on HIV status and is not associated with poor delivery outcomes**. *J Infect Dis* 2010, **201**(1):123-131.

109. Aitken EH, Mbewe B, Luntamo M, Kulmala T, Beeson JG, Ashorn P, Rogerson SJ: **Antibody to P. falciparum in pregnancy varies with intermittent preventive treatment regime and bed net use**. *Plos One* 2012, **7**(1):e29874.

110. Duffy MF, Maier AG, Byrne TJ, Marty AJ, Elliott SR, O'Neill MT, Payne PD, Rogerson SJ, Cowman AF, Crabb BS *et al*: **VAR2CSA is the principal ligand for chondroitin sulfate A in two allogeneic isolates of Plasmodium falciparum**. *Molecular and Biochemical Parasitology* 2006, **148**(2):117-124.

111. Fried M, Nosten F, Brockman A, Brabin BT, Duffy PE: **Maternal antibodies block malaria**. *Nature* 1998, **395**(6705):851-852.

112. Babakhanyan A, Tutterrow YL, Bobbili N, Salanti A, Wey A, Fogako J, Leke RJ, Leke RG, Taylor DW: **Influence of Intermittent Preventive Treatment on Antibodies to VAR2CSA in Pregnant Cameroonian Women**. *Am J Trop Med Hyg* 2016, **94**(3):640-649.

113. Brolin KJM, Persson KEM, Wahlgren M, Rogerson SJ, Chen QJ: **Differential Recognition of P. falciparum VAR2CSA Domains by Naturally Acquired Antibodies in Pregnant Women from a Malaria Endemic Area**. *Plos One* 2010, **5**(2).

114. Chia YS, Badaut C, Tuikue Ndam NG, Khattab A, Igonet S, Fievet N, Bentley GA, Deloron P, Klinkert MQ: **Functional and immunological characterization of a duffy binding-like- gamma domain from Plasmodium falciparum erythrocyte membrane protein-1 expressed by a placental isolate**. *J Infect Dis* 2005, **192**(7):1284-1293.

115. Fievet N, Le Hesran JY, Cottrell G, Doucoure S, Diouf I, Ndiaye JL, Bertin G, Gaye O, Sow S, Deloron P: **Acquisition of antibodies to variant antigens on the surface of Plasmodium falciparum-infected erythrocytes during pregnancy**. *Infection Genetics and Evolution* 2006, **6**(6):459-463.

116. Gangnard S, Badaut C, Ramboarina S, Baron B, Ramdani T, Gamain B, Deloron P, Lewit-Bentley A, Bentley GA: **Structural and Immunological Correlations between the Variable Blocks of the VAR2CSA Domain DBL6 epsilon from Two Plasmodium falciparum Parasite Lines**. *Journal of Molecular Biology* 2013, **425**(10):1697-1711.

117. Jaworowski A, Fernandes LA, Yosaatmadja F, Feng G, Mwapasa V, Molyneux ME, Meshnick SR, Lewis J, Rogerson SJ: **Relationship between human immunodeficiency virus type 1 coinfection, anemia, and levels and function of antibodies to variant surface antigens in pregnancy-associated malaria**. *Clin Vaccine Immunol* 2009, **16**(3):312-319.

118. Khattab A, Chia YS, May J, Le Hesran JY, Deloron P, Klinkert MQ: **The impact of IgG antibodies to recombinant Plasmodium falciparum 732var CIDR-1 alpha domain in mothers and their newborn babies**. *Parasitology Research* 2007, **101**(3):767-774.

119. Mayor A, Serra-Casas E, Rovira-Vallbona E, Jimenez A, Quinto L, Sigauque B, Dobano C, Bardaji A, Alonso PL, Menendez C: **Immunoglobulins against the surface of Plasmodium falciparum-infected erythrocytes increase one month after delivery**. *Malaria Journal* 2012, **11**.

120. Mount AM, Mwapasa V, Elliott SR, Beeson JG, Tadesse E, Lema VM, Molyneux ME, Meshnick SR, Rogerson SJ: **Impairment of humoral immunity to Plasmodium falciparum malaria in pregnancy by HIV infection**. *Lancet* 2004, **363**(9424):1860-1867.

121. Naniche D, Serra-Casas E, Bardaji A, Quinto L, Dobano C, Sigauque B, Cistero P, Chauhan VS, Chitnis CE, Alonso PL *et al*: **Reduction of Antimalarial Antibodies by HIV Infection Is Associated With Increased Risk of Plasmodium falciparum Cord Blood Infection**. *Journal of Infectious Diseases* 2012, **205**(4):568-577.

122. Taylor DW, Zhou A, Marsillio LE, Thuita LW, Leke EB, Branch O, Gowda DC, Long C, Leke RF: **Antibodies that inhibit binding of Plasmodium falciparum-infected erythrocytes to chondroitin sulfate A and to the C terminus of merozoite surface protein 1 correlate with reduced placental malaria in Cameroonian women**. *Infect Immun* 2004, **72**(3):1603-1607.
