# Supplementary material for: Pregnancy-specific malarial immunity and risk of malaria in pregnancy and adverse birth outcomes: a systematic review
Source: BMC Med. 2020 Jan 16;18:14. doi: 10.1186/s12916-019-1467-6 (PMC6964062; doi:10.1186/s12916-019-1467-6)
Supplement: Supplementary file 6 — Additional file 6. Details of parasites and recombinant antigens featured in review. [file 12916_2019_1467_MOESM6_ESM.docx]

Additional file 6: Details of antigens included in review.

| **Parasitized red blood cells (pRBC)** | | | |
| --- | --- | --- | --- |
| **Author, year** | **Parasite Isolate/Strain** | **Details** | **Parasite selection** |
| Aitken, 2010 | CS2^a^ | Established reference isolate/ *in vitro* adapted line | Not described, but line is CSA adherent |
| Ataide 2010, 2011 | CS2 | Established reference isolate/ *in vitro* adapted line | Not described, but line is CSA adherent |
| Beeson, 2004 | CS2 | Established reference isolate/ *in vitro* adapted line | Adhesion to CSA |
| Chandrasiri, 2014 | CS2 | Established reference isolate/ *in vitro* adapted line | Not described, but line is CSA adherent |
| Chandrasiri, 2016 | CS2 | Established reference isolate/ *in vitro* adapted line | Not described, but line is CSA adherent |
| Cox, 2005 | FCR3^a^ | Established reference isolate/ *in vitro* adapted line | Adhesion to CSA |
| Duffy, 2003 | Placental isolates | Freshly collected isolates from infected placentas in Kisumu, Kenya | NA |
| Feng, 2009 | CS2 | Established reference isolate/ *in vitro* adapted line | Not described, but line is CSA adherent |
| Hommel, 2010 | CS2 | Established reference isolate/ *in vitro* adapted line | Adhesion to CSA |
|  | Pf2006-CSA | Isolate from traveller who contracted malaria in Ghana | Adhesion to CSA |
|  | Pf2004-CSA | Isolate from traveller who contracted malaria in Ghana | Adhesion to CSA |
|  | 3D7-CSA | Established reference isolate/ *in vitro* adapted line | Adhesion to CSA |
|  | HCS3 | Isolated from traveller who contracted malaria in South East Asia | Adhesion to CSA |
|  | HB3-CSA | Established reference isolate/ *in vitro* adapted line | Adhesion to CSA |
|  | XIE-CSA | Isolated from peripheral blood of pregnant woman in PNG | Adhesion to CSA |
| Khattab, 2004 | Gb218 | Placental isolate from Gabonese study cohort | Adhesion to CSA |
|  | Gb337 | Placental isolate from Gabonese study cohort | Adhesion to CSA |
|  | vip43 | Placental isolate from Senegalese study cohort | Adhesion to CSA |
|  | vip42 | Placental isolate from Senegalese study cohort | Adhesion to CSA |
| Megnekou, 2005 | FCR3 | Established reference isolate/ *in vitro* adapted line | Preselection for non-adhesion to CHO-745 cells that do not express chondroitin sulfate proteoglycan, followed by selection for binding to CHO-K1 cells that express chondroitin sulfate proteoglycan |
| Mayor, 2011 | 193T_CSA_ | Established reference isolate/ *in vitro* adapted line | Not described, but line is CSA adherent |
|  | CS2_CSA_ | Established reference isolate/ *in vitro* adapted line (MRA-96 from MR4, Manassas, VA) | Not described, but line is CSA adherent |
|  | FCR3_CSA_ | Established reference isolate/ *in vitro* adapted line | Not described, but line is CSA adherent |
|  | Plac1-4 | Placental isolates | NA |
|  | Mot1-8 | Peripheral blood isolates from pregnant women | NA |
| Mayor, 2013 | Plac1-2 | Placental isolates | NA |
| O'Neil-Dunne, 2001 | 3D7 | Established reference isolate/ *in vitro* adapted line | Initially selected for adherence to bovine trachea C4S, then to CSPG from human placenta. |
| Serra-Casas, 2010 | CS2 | Established reference isolate/ *in vitro* adapted line | Not described, but line is CSA adherent |
| Staalsoe, 2001 | Palo Alto (PA) | Established reference isolate/ *in vitro* adapted line | Adhesion to CSA |
| Staalsoe, 2004 | EJ24^a^ | Placental isolate | Not described, but isolate is CSA adherent. |
| Teo, 2014 | CS2 | Established reference isolate/ *in vitro* adapted line | Not described, but line is CSA adherent |
| Tuikue Ndam, 2015 | FCR3 | Established reference isolate/ *in vitro* adapted line | Panning on BeWo cells |
| Tutterrow 2012 (PO) | 7G8 | Established reference isolate/ *in vitro* adapted line | Adhesion to CSA |

^a^Additional parasite lines were studied, but the isolate with the most seroprevalent response was selected for inclusion in this review.

|  | **Recombinant VAR2CSA antigens** | | | |
| --- | --- | --- | --- | --- |
| **Author, year** | **VAR2CSA domain** | **Allele/Strain** | **Domain boundaries (amino acids)** | **Expression system** |
| Babakhanyan, 2014 | FV2 | FCR3 | 1-2649 | Baculovirus/Sf9 |
|  | ID1-ID2a | FCR3 | DNS | Baculovirus/Sf9 |
| Babakhanyan, 2015 | FV2 | FCR3 | 1-2649 | Baculovirus/Sf9 |
|  | DBL1 | 3D7 | 58-433 | *Pichia pastoris* |
|  | DBL1 | 7G8 | 57-437 | *Pichia pastoris* |
|  | DBL1+2 | FCR3 | 1-893 | Baculovirus/Sf9 |
|  | DBL2 | FCR3 | DNS | Baculovirus/Sf9 |
|  | DBL3 | FCR3 | 1210-1587 | Baculovirus/Sf9 |
|  | DBL3 | 7G8 | 1199-1587 | *Pichia pastoris* |
|  | DBL3 | FCR3 | DNS | *E. coli* |
|  | DBL4 | FCR3 | 1583-1947 | Baculovirus/Sf9 |
|  | DBL4 | 7G8 | 1604-1942 | *Pichia pastoris* |
|  | DBL4 | FCR3/IT4 | DNS | *Pichia pastoris* |
|  | DBL5 | FCR3 | 1990-2328 | Baculovirus/Sf9 |
|  | DBL5 | 3D7 | 1888-2291 | *Pichia pastoris* |
|  | DBL5 | 7G8 | 2000-2318 | *Pichia pastoris* |
|  | DBL6 | FCR3 | 2307-2641 | Baculovirus/Sf9 |
|  | DBL6 | 7G8 | 2322-2590 | *Pichia pastoris* |
|  | DBL6 | FCR3/IT4 | DNS | *Pichia pastoris* |
| Chandrasiri, 2014 | DBL5Ɛ | 3D7 | 1888-2291^a^ | *Pichia pastoris* |
| Fowkes, 2012 | DBL5Ɛ | 3D7 | 1888-2291^a^ | *Pichia pastoris* |
| Fried, 2018 | DBL2 | FCR3 | 543-858 | *E. coli* |
|  | ID1-ID2a | Maternal isolate 1010 | 428-1024^b^ | Baculovirus |
|  | DBL3 | FCR3 | 1220-1541 | *E. coli* |
|  | DBL3-4 | FCR3 | 1445-1989 | *E. coli* |
|  | DBL4 | Maternal isolate 1010^c^ | 1583-1989^b^ | *E. coli* |
|  | DBL5 | Maternal isolate 466^d^ | 2003-2281^b^ | *E. coli* |
| Gnidehou, 2014 | ID1-ID2 | FCR3 | DNS | Baculovirus/Sf9 |
|  | DBL3X | FCR3 | DNS | Baculovirus/Sf9 |
|  | DBL5ε | FCR3 | DNS | Baculovirus/Sf9 |
| Guitard, 2008 | DBL5ε | 3D7 | DNS | Baculovirus/Sf9 |
| Lloyd, 2018 | FV2 | FCR3 | 1-2649 | Baculovirus/Sf9 |
| Mayor, 2013 | DBL2X | 3D7 | 535 to 934 | *E. coli* |
|  | DBL3X | 3D7 | 1214 to 1562 | *E. coli* |
|  | DBL5Ɛ | 3D7 | 1983 to 2291 | *E. coli* |
|  | DBL6Ɛ | 3D7 | 2333 to 2617 | *E. coli* |
| McLean, 2017 | DBL5 | 7G8 | 2003-2270 | DNS |
| Salanti, 2004 | DBL5ε | 3D7 | DNS | Baculovirus/Sf9 |
| Siriwardhana, 2017 | FV2 | FCR3 | 1-2649 | Baculovirus/Sf9 |
|  | DBL1 | IT4 | DNS | DNS |
|  | DBL1 | 3D7 | 58-433 | *Pichia pastoris* |
|  | DBL1 | 7G8 | 57-437 | *Pichia pastoris* |
|  | DBL1+2 | FCR3 | 1-893 | Baculovirus/Sf9 |
|  | ID1-ID2a | FCR3 | DNS | DNS |
|  | ID1-ID2a | 3D7 | DNS | DNS |
|  | DBL2 | FCR3 | DNS | DNS |
|  | DBL3 | FCR3 | 1210-1587 | Baculovirus/Sf9 |
|  | DBL3 | 7G8 | 1199-1587 | *Pichia pastoris* |
|  | DBL4 | FCR3 | DNS | DNS |
|  | DBL4 | 7G8 | 1604-1942 | *Pichia pastoris* |
|  | DBL5 | FCR3 | 1990-2328 | Baculovirus/Sf9 |
|  | DBL5 | 3D7 | 1888-2291 | *Pichia pastoris* |
|  | DBL5 | 7G8 | 2000-2318 | *Pichia pastoris* |
|  | DBL6 | FCR3 | 2307-2641 | Baculovirus/Sf9 |
|  | DBL6 | 7G8 | 2322-2590 | *Pichia pastoris* |
| Teo, 2014 | DBL5ε | 3D7 | 1888-2291^a^ | *Pichia pastoris* |
| Tuikue Ndam, 2006 | DBL1x | 3D7 | DNS | Baculovirus/Sf9 |
|  | DBL5ε | 3D7 | DNS | Baculovirus/Sf9 |
|  | DBL6ε | 3D7 | DNS | Baculovirus/Sf9 |
| Tuikue Ndam, 2015 | FV2 | FCR3 | 1-2649 | Baculovirus/Sf9 |
|  | DBL1-DBL2 | FCR3 | DNS | Baculovirus/Sf9 |
|  | DBL3 | FCR3 | DNS | Baculovirus/Sf9 |
|  | DBL4 | FCR3 | DNS | Baculovirus/Sf9 |
|  | DBL5 | FCR3 | DNS | Baculovirus/Sf9 |
|  | DBL6 | FCR3 | DNS | Baculovirus/Sf9 |
| Tutterrow, 2012 (I&I) | DBL1 | FCR3 | 58-383 | *Pichia pastoris* |
|  | DBL1 | 3D7 | 58-433 | *Pichia pastoris* |
|  | DBL1 | 7G8 | 57-437 | *Pichia pastoris* |
|  | DBL1+2 | FCR3 | 1-893 | Baculovirus/Sf9 |
|  | DBL3 | FCR3 | 1210-1587 | Baculovirus/Sf9 |
|  | DBL3 | 7G8 | 1199-1587 | *Pichia pastoris* |
|  | DBL4 | FCR3 | 1594-1888 | *Pichia pastoris* |
|  | DBL4 | FCR3 | 1583-1947 | Baculovirus/Sf9 |
|  | DBL4 | 7G8 | 1604-1942 | *Pichia pastoris* |
|  | DBL5 | 7G8 | 2000-2318 | *Pichia pastoris* |
|  | DBL5 | 3D7 | 1888-2291 | *Pichia pastoris* |
|  | DBL5 | FCR3 | 1990-2328 | Baculovirus/Sf9 |
|  | DBL6 | FCR3 | 2322-2590 | *Pichia pastoris* |
|  | DBL6 | 7G8 | 2322-2590 | *Pichia pastoris* |
|  | DBL6 | FCR3 | 2307-2641 | Baculovirus/Sf9 |
| Tutterrow, 2012 (PO) | FV2 | FCR3 | 1-2649 | Baculovirus/Sf9 |

^a^N-terminal truncation occurred at amino acid position 95, resulting in loss of ~10kDa.

^b^Domain boundaries refers to the amino acids in the homologous sequences VAR2CSA-FCR3

^c^Fried et al also measured antibody responses to additional DBL4 constructs (alleles FCR3, 3D7, and maternal isolate 711) but only responses to DBL4 maternal isolate 1010 are included in this review, as this construct elicited the most seroprevalent response in the cohort.

^d^Fried et al also measured antibody responses to additional DBL5 constructs (alleles FCR3 and maternal isolate 1010) but only responses to DBL5 maternal isolate 466 are included in this review, as this construct elicited the most seroprevalent response in the cohort.
