# Supplementary material for: Pregnancy-specific malarial immunity and risk of malaria in pregnancy and adverse birth outcomes: a systematic review
Source: BMC Med. 2020 Jan 16;18:14. doi: 10.1186/s12916-019-1467-6 (PMC6964062; doi:10.1186/s12916-019-1467-6)
Supplement: Supplementary file 7 — Additional file 7. Supplementary forest plots and results for additional outcomes. [file 12916_2019_1467_MOESM7_ESM.pdf]

Additional File 7: Supplementary forest plots and results for additional outcomes.

Anaemia

Four studies provided estimates for the association between antibody responses to pregnancy specific *P. falciparum* antigens and maternal anaemia at delivery [1-4], and one study examined antibody responses and anaemia during the second and third trimester [2]. Whilst the definitions of anaemia differed in each study, the overall trend was for increased odds or risk of anaemia in antibody responders compared to non-responders (Supplementary Figure 1, Additional file 7) [1-4]. Notably, Kenyan Ab responders to a placental isolate (EJ24) had a 2.6-fold increase in odds of severe anaemia (Hb <7.0g/dl; 95%CI, 1.81-3.68) compared to non-responders (Supplementary Figure 1, Additional file 7)[1]. Five studies included in narrative form found no association between antibodies to pregnancy-specific pRBC or VAR2CSA antigens and anaemia [5-9] or haematocrit [10]. In contrast, in studies of Malawian women, total IgG and opsonizing Abs to CS2 were positively associated with haemoglobin levels at 36 weeks [11] and decreased anaemia at delivery [12], suggesting antibodies may have a protective effect. Overall, the associations between antibody responses to pregnancy-specific *P. falciparum* antigens and anaemia were heterogeneous, and this is probably reflective of differences in the populations studied, in the specific outcome measurements, and the multiple (non-malarial) causes of anemia.

Severe malaria

A single Sudanese case-control study examined antibody responses in three groups of pregnant women: those with severe malaria, those with uncomplicated malaria, and uninfected controls. Women who were positive for total IgG or opsonic phagocytosis Abs to CS2 pRBC at T2/T3 had reduced odds of severe malaria, when uninfected controls were the reference group, compared to women who were negative for these antibodies (Supplementary Figure 2, Additional file 7); but this association was significant for phagocytic antibodies only [13]. In contrast, DBL5 responders had a non-significant increase in odds of severe malaria compared to non-responders, when uninfected controls were the reference group (Supplementary Figure 2, Additional file 7).

Preterm birth

Three studies provided estimates for the association between antibody responses to pregnancy-specific *P. falciparum* antigens at delivery and odds of preterm birth (PTB), with heterogeneous results (Supplementary Figures 3A, 11A, and 12A, Additional file 7). At delivery, Kenyan women positive for CSA adhesion inhibitory Abs [4] and Malian women positive for DBL3 Abs [14] had reduced odds of PTB, but Ab responders to FV2 had increased odds of PTB compared to non-responders [3]. Only two studies examined the association between antibody responses measured earlier in pregnancy and risk of PTB. Aitken et al found no association between antibodies to CS2, measured at T2 or T3, and PTB (Supplementary Figure 3B, Additional file 7)[2]. Fried et al found that antibody responders to ID1-ID2a at enrolment had increased odds of PTB compared to non-responders (Supplementary Figure 3B, Additional file 7), but this association was not observed for other VAR2CSA antigens, nor in secundigravidae/multigravidae (Supplementary Figure 12B,

Additional file 7) [14]. Conversely, among secundigravidae/multigravidae only, antibody responders to DBL3 at enrolment had decreased odds of PTB, but this association was not observed for other antigens (Supplementary Figure 12B, additional file 7).
Of the studies included in narrative form, one examined PTB as an outcome and found that of those women negative for peripheral infection at enrolment, higher CSPG-binding inhibitory capacity, but not total IgG, was associated with a lower risk PTB [8]. In addition, one study examined the association between antibody responses to a range of pRBC and VAR2CSA antigens and found that higher Abs against DBL2X were associated with younger gestational age and among women with at least one malaria episode during pregnancy, high Abs to a placental isolate, DBL3X and DBL6Ɛ were associated with increased gestational age [10].

References

1. Staalsoe T, Shulman CE, Bulmer JN, Kawuondo K, Marsh K, Hviid L: **Variant surface antigen-specific IgG and protection against clinical consequences of pregnancy-associated Plasmodium falciparum malaria**. *Lancet* 2004, **363**(9405):283-289.
2. Aitken EH, Mbewe B, Luntamo M, Maleta K, Kulmala T, Friso MJ, Fowkes FJI, Beeson JG, Ashorn P, Rogerson SJ: **Antibodies to Chondroitin Sulfate A- Binding Infected Erythrocytes: Dynamics and Protection during Pregnancy in Women Receiving Intermittent Preventive Treatment**. *Journal of Infectious Diseases* 2010, **201**(9):1316-1325.
3. Lloyd YM, Fang R, Bobbili N, Vanda K, Ngati E, Sanchez-Quintero MJ, Salanti A, Chen JJ, Leke RGF, Taylor DW: **Association of Antibodies to VAR2CSA and Merozoite Antigens with Pregnancy Outcomes in Women Living in Yaounde, Cameroon**. *Infect Immun* 2018, **86**(9).
4. Duffy PE, Fried M: **Antibodies that inhibit Plasmodium falciparum adhesion to chondroitin sulfate A are associated with increased birth weight and the gestational age of Newborns**. *Infection and Immunity* 2003, **71**(11):6620-6623.
5. Ataide R, Hasang W, Wilson DW, Beeson JG, Mwapasa V, Molyneux ME, Meshnick SR, Rogerson SJ: **Using an Improved Phagocytosis Assay to Evaluate the Effect of HIV on Specific Antibodies to Pregnancy-Associated Malaria**. *Plos One* 2010, **5**(5).
6. Ataide R, Mwapasa V, Molyneux ME, Meshnick SR, Rogerson SJ: **Antibodies That Induce Phagocytosis of Malaria Infected Erythrocytes: Effect of HIV Infection and Correlation with Clinical Outcomes**. *Plos One* 2011, **6**(7).
7. Tuikue Ndam NG, Salanti A, Le-Hesran JY, Cottrell G, Fievet N, Turner L, Sow S, Dangou JM, Theander T, Deloron P: **Dynamics of Anti-VAR2CSA immunoglobulin G response in a cohort of senegalese pregnant women**. *Journal of Infectious Diseases* 2006, **193**(5):713-720.
8. Tuikue Ndam N, Denoeud-Ndam L, Doritchamou J, Viwami F, Salanti A, Nielsen MA, Fievet N, Massougbodji A, Luty AJ, Deloron P: **Protective Antibodies against Placental Malaria and Poor Outcomes during Pregnancy, Benin**. *Emerging infectious diseases* 2015, **21**(5):813-823.
9. Serra-Casas E, Menendez C, Bardaji A, Quinto L, Dobano C, Sigauque B, Jimenez A, Mandomando I, Chauhan VS, Chitnis CE *et al*: **The effect of intermittent preventive**

treatment during pregnancy on malarial antibodies depends on HIV status and is not associated with poor delivery outcomes. *J Infect Dis* 2010, **201**(1):123-131.
10. Mayor A, Kumar U, Bardaji A, Gupta P, Jimenez A, Hamad A, Sigauque B, Singh B, Quinto L, Kumar S *et al*: **Improved Pregnancy Outcomes in Women Exposed to Malaria With High Antibody Levels Against Plasmodium falciparum**. *Journal of Infectious Diseases* 2013, **207**(11):1664-1674.
11. Chandrasiri UP, Fowkes FJ, Beeson JG, Richards JS, Kamiza S, Maleta K, Ashorn P, Rogerson SJ: **Association between malaria immunity and pregnancy outcomes among Malawian pregnant women receiving nutrient supplementation**. *Malar J* 2016, **15**(1):547.
12. Feng GQ, Aitken E, Yosaatmadja F, Kalilani L, Meshnick SR, Jaworowski A, Simpson JA, Rogerson SJ: **Antibodies to Variant Surface Antigens of Plasmodium falciparum-Infected Erythrocytes Are Associated with Protection from Treatment Failure and the Development of Anemia in Pregnancy**. *Journal of Infectious Diseases* 2009, **200**(2):299-306.
13. Chandrasiri UP, Randall LM, Saad AA, Bashir AM, Rogerson SJ, Adam I: **Low antibody levels to pregnancy-specific malaria antigens and heightened cytokine responses associated with severe malaria in pregnancy**. *J Infect Dis* 2014, **209**(9):1408-1417.
14. Fried M, Kurtis JD, Swihart B, Morrison R, Pond-Tor S, Barry A, Sidibe Y, Keita S, Mahamar A, Andemel N *et al*: **Antibody levels to recombinant VAR2CSA domains vary with Plasmodium falciparum parasitaemia, gestational age, and gravidity, but do not predict pregnancy outcomes**. *Malar J* 2018, **17**(1):106.

Study	Country	Design	Ab time	Test time	Assay	Antigen	Anaemia def.	n	ES (95% CI)
Aitken et al 2010	Malawi	CS	T2	T2	Flow cyto.	CS2	Hb <110 g/l	508	0.99 (0.52, 1.86)[a]
Aitken et al 2010	Malawi	RCT	T2	T3	Flow cyto.	CS2	Hb <110 g/l	472	1.43 (0.86, 2.38)[a]
Aitken et al 2010	Malawi	CS	T3	T3	Flow cyto.	CS2	Hb <110 g/l	88	1.94 (0.43, 8.66)[a]
Staalsoe et al 2004	Kenya	CS	delivery	delivery	Flow cyto.	EJ24	Hb <70 g/l	582	2.58 (1.81, 3.68)[a]
Duffy & Fried 2003	Kenya	CS	delivery	delivery	CSA adhes. inhib.	plac. isolate	Hb <110 g/l	170	1.29 (0.61, 2.72)[a]
Lloyd et al 2018	Cameroon	CC	delivery	delivery	Multiplex	FV2	<30% PCV	1342	1.32 (0.98, 1.76)[b]

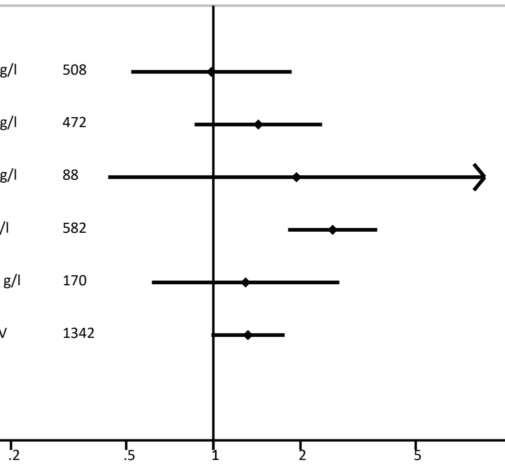

Supplementary Figure 1. Forest plot of the association between antibodies to pregnancy-associated *P. falciparum* antigens and anaemia. Estimates represent the odds (CS and CC studies) or risk (RCT) of anaemia or severe anaemia, as defined in individual publications, in Ab responders compared to Ab non-responders and are for women of all gravidities included in original studies. Timing of antibody determination (Ab time) and anaemia determination (Test time) are as indicated. [a]Data supplied by original authors and estimate calculated by current authors; [b]Estimate calculated by current authors from data in original publication. CS, cross-sectional; CSA adhes. inhib., CSA adhesion inhibition assay; CC, case-control; ES, estimate; Flow cyto., flow cytometry; Hb, haemoglobin; n, number of participants included in estimate; plac. isolate, placental isolate; PCV, packed cell volume; RCT, randomized controlled trial; T2, second trimester; T3, third trimester.

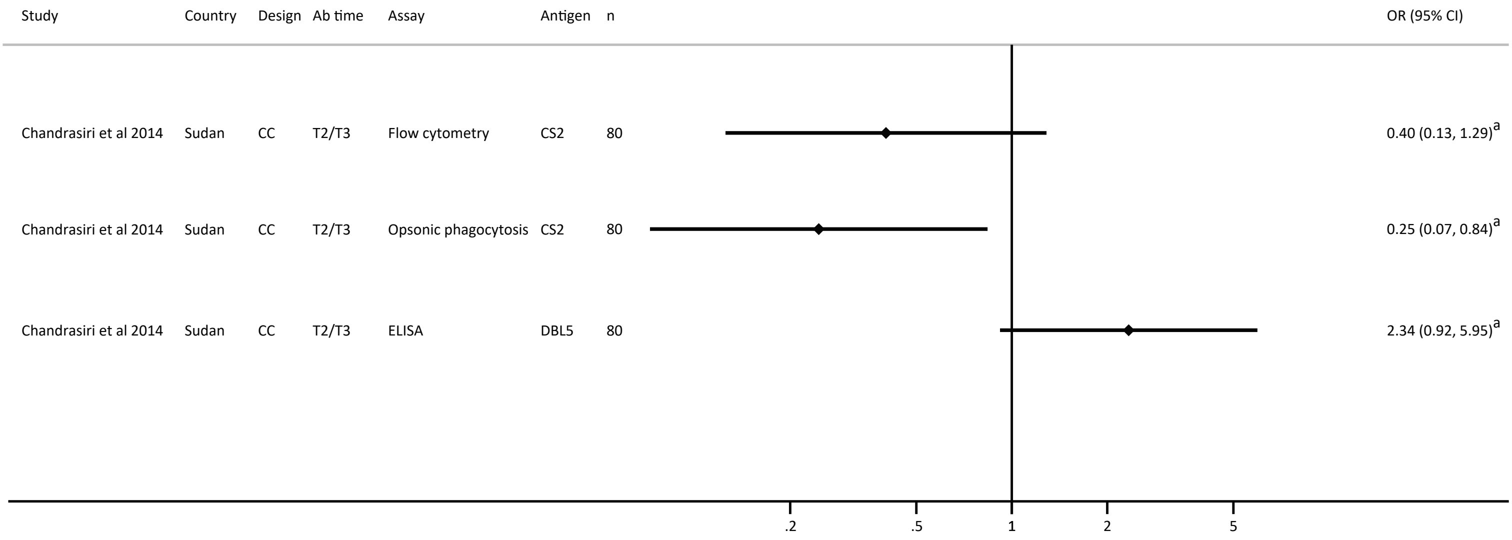

Supplementary Figure 2. Forest plot of the association between antibodies to pregnancy-associated *P. falciparum* antigens and severe malaria. Estimates represent the odds of severe malaria (compared to uninfected women) in Ab responders compared to Ab non-responders and are for women of all gravidities. [a]Estimate calculated by current authors from data in original publication. CC, case control; n, number of participants included in estimate; OR, odds ratio; T2, second trimester; T3, third trimester.

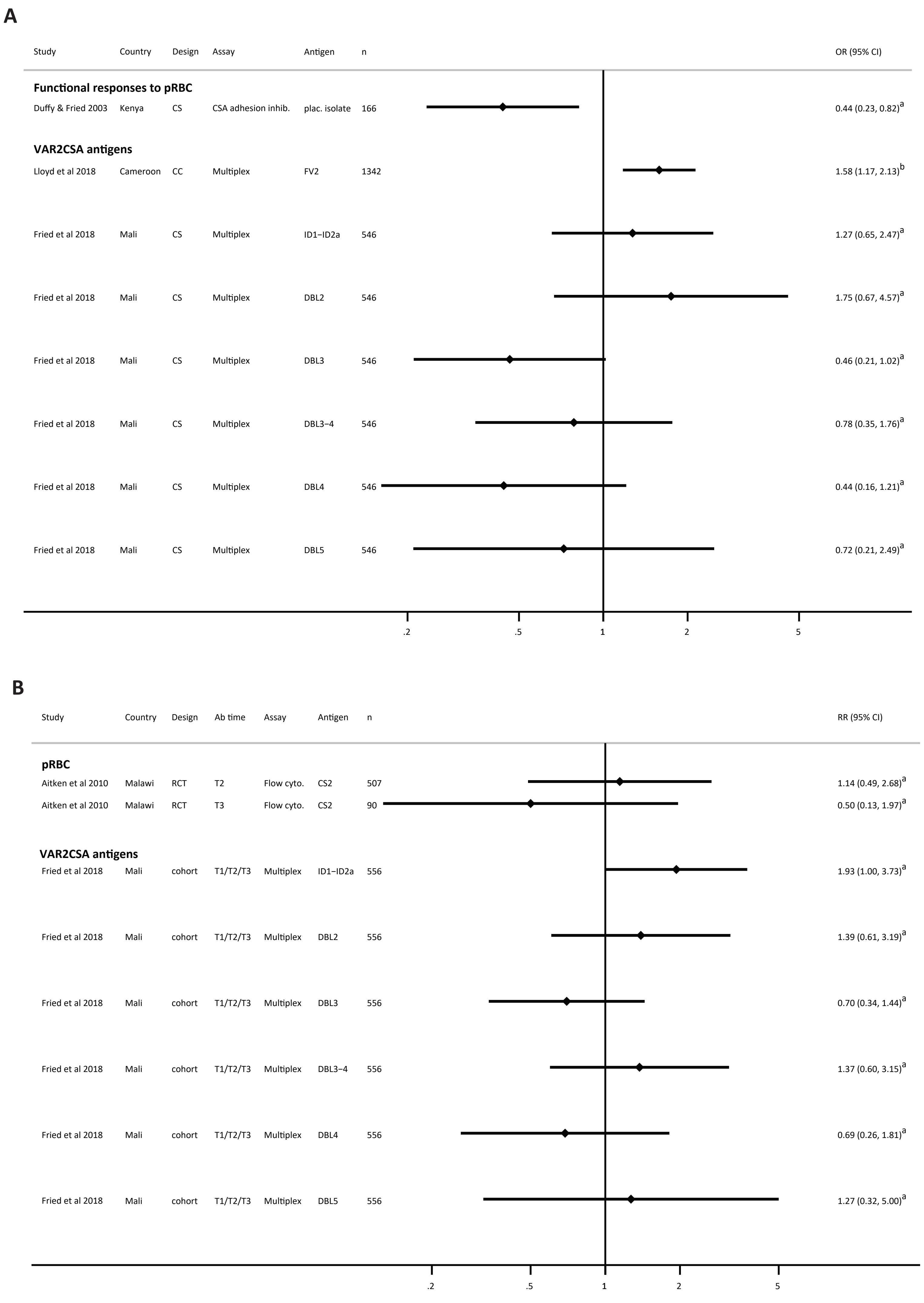
A
Study
Country
Design
Assay
Antigen
n
OR (95% CI)
Functional responses to pRBC
Duffy & Fried 2003 Kenya CS CSA adhesion inhib. plac. isolate 166 0.44 (0.23, 0.82)[a]
VAR2CSA antigens
Lloyd et al 2018 Cameroon CC Multiplex FV2 1342 1.58 (1.17, 2.13)[b]
Fried et al 2018 Mali CS Multiplex ID1–ID2a 546 1.27 (0.65, 2.47)[a]
Fried et al 2018 Mali CS Multiplex DBL2 546 1.75 (0.67, 4.57)[a]
Fried et al 2018 Mali CS Multiplex DBL3 546 0.46 (0.21, 1.02)[a]
Fried et al 2018 Mali CS Multiplex DBL3–4 546 0.78 (0.35, 1.76)[a]
Fried et al 2018 Mali CS Multiplex DBL4 546 0.44 (0.16, 1.21)[a]
Fried et al 2018 Mali CS Multiplex DBL5 546 0.72 (0.21, 2.49)[a]
.2
.5
1
2
5
B
Study
Country
Design
Ab time
Assay
Antigen
n
RR (95% CI)
pRBC
Aitken et al 2010 Malawi RCT T2 Flow cyto. CS2 507 1.14 (0.49, 2.68)[a]
Aitken et al 2010 Malawi RCT T3 Flow cyto. CS2 90 0.50 (0.13, 1.97)[a]
VAR2CSA antigens
Fried et al 2018 Mali cohort T1/T2/T3 Multiplex ID1–ID2a 556 1.93 (1.00, 3.73)[a]
Fried et al 2018 Mali cohort T1/T2/T3 Multiplex DBL2 556 1.39 (0.61, 3.19)[a]
Fried et al 2018 Mali cohort T1/T2/T3 Multiplex DBL3 556 0.70 (0.34, 1.44)[a]
Fried et al 2018 Mali cohort T1/T2/T3 Multiplex DBL3–4 556 1.37 (0.60, 3.15)[a]
Fried et al 2018 Mali cohort T1/T2/T3 Multiplex DBL4 556 0.69 (0.26, 1.81)[a]
Fried et al 2018 Mali cohort T1/T2/T3 Multiplex DBL5 556 1.27 (0.32, 5.00)[a]
.2
.5
1
2
5

Supplementary Figure 3. Forest plot of the association between antibodies to pregnancy-associated *P. falciparum* antigens and preterm birth. A. Estimates represent the odds of preterm birth in Ab responders compared to Ab non-responders, where antibodies were measured at delivery (cross-sectional studies). B. Estimates represent the risk of preterm birth in Ab responders compared to non-responders, where antibodies were measured at time-points prior to delivery, as indicated (prospective studies). Estimates are for women of all gravidities. Meta-analysis was not performed as antigens differed between studies [a]Data supplied by original authors and estimate calculated by current authors. CC, case control; CS, cross-sectional; CSA adhesion inhib., CSA adhesion inhibition assay; Flow cyto., flow cytometry; n, number of participants included in estimate; OR, odds ratio; plac. isolate, placental isolate; pRBC, parasitized red blood cells; RCT, randomized controlled trial; RR, risk ratio; T1, first trimester; T2, second trimester; T3, third trimester.

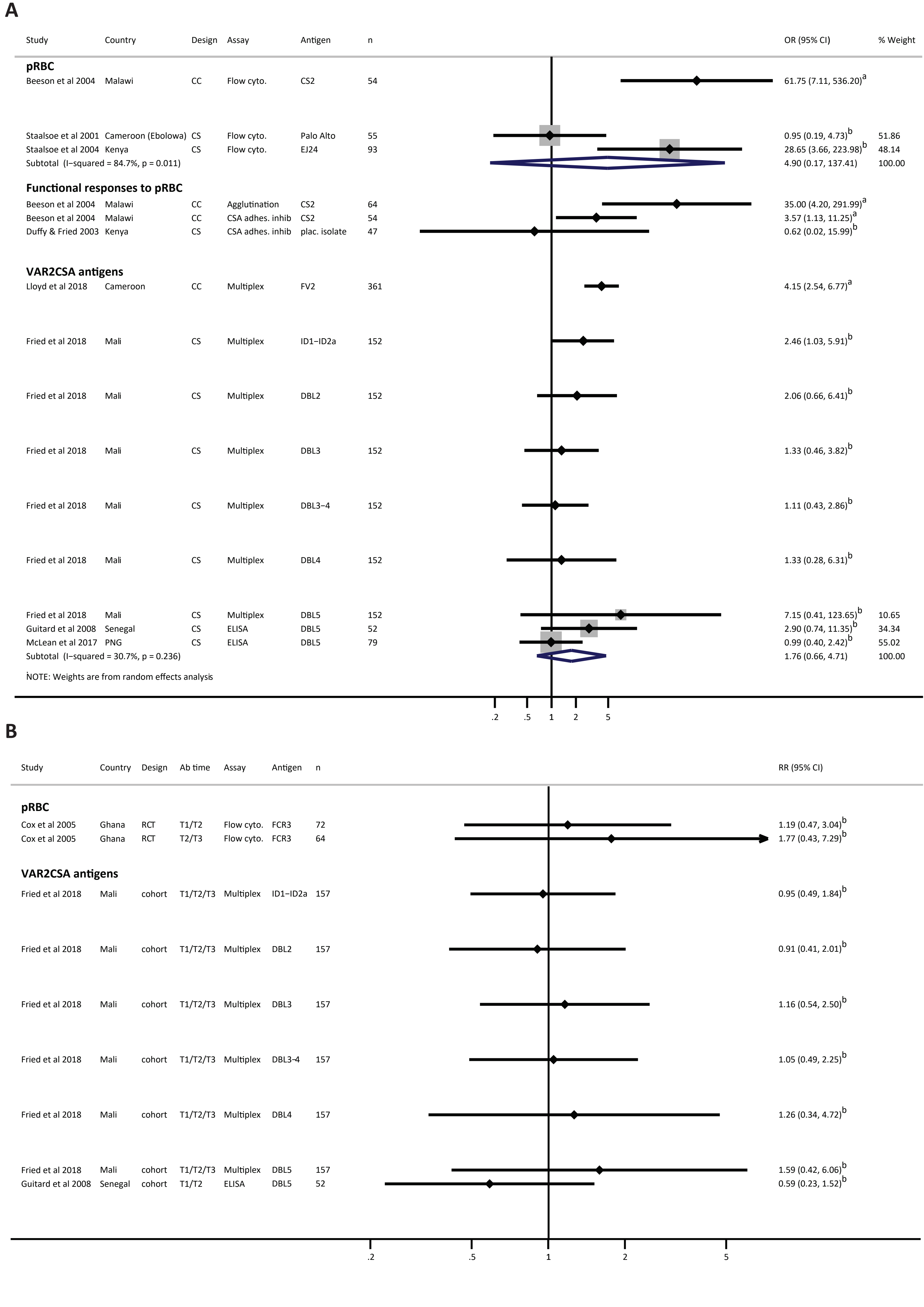
A
Study Country Design Assay Antigen n OR (95% CI) % Weight
pRBC
Beeson et al 2004 Malawi CC Flow cyto. CS2 54 61.75 (7.11, 536.20)a
Staalsoe et al 2001 Cameroon (Ebolowa) CS Flow cyto. Palo Alto 55 0.95 (0.19, 4.73)b 51.86
Staalsoe et al 2004 Kenya CS Flow cyto. EJ24 93 28.65 (3.66, 223.98)b 48.14
Subtotal (I−squared = 84.7%, p = 0.011) 4.90 (0.17, 137.41) 100.00
Functional responses to pRBC
Beeson et al 2004 Malawi CC Agglutination CS2 64 35.00 (4.20, 291.99)a
Beeson et al 2004 Malawi CC CSA adhes. inhib CS2 54 3.57 (1.13, 11.25)a
Duffy & Fried 2003 Kenya CS CSA adhes. inhib plac. isolate 47 0.62 (0.02, 15.99)b
VAR2CSA antigens
Lloyd et al 2018 Cameroon CC Multiplex FV2 361 4.15 (2.54, 6.77)a
Fried et al 2018 Mali CS Multiplex ID1−ID2a 152 2.46 (1.03, 5.91)b
Fried et al 2018 Mali CS Multiplex DBL2 152 2.06 (0.66, 6.41)b
Fried et al 2018 Mali CS Multiplex DBL3 152 1.33 (0.46, 3.82)b
Fried et al 2018 Mali CS Multiplex DBL3−4 152 1.11 (0.43, 2.86)b
Fried et al 2018 Mali CS Multiplex DBL4 152 1.33 (0.28, 6.31)b
Fried et al 2018 Mali CS Multiplex DBL5 152 7.15 (0.41, 123.65)b 10.65
Guitard et al 2008 Senegal CS ELISA DBL5 52 2.90 (0.74, 11.35)b 34.34
McLean et al 2017 PNG CS ELISA DBL5 79 0.99 (0.40, 2.42)b 55.02
Subtotal (I−squared = 30.7%, p = 0.236) 1.76 (0.66, 4.71) 100.00
NOTE: Weights are from random effects analysis
.2 .5 1 2 5
B
Study Country Design Ab time Assay Antigen n RR (95% CI)
pRBC
Cox et al 2005 Ghana RCT T1/T2 Flow cyto. FCR3 72 1.19 (0.47, 3.04)b
Cox et al 2005 Ghana RCT T2/T3 Flow cyto. FCR3 64 1.77 (0.43, 7.29)b
VAR2CSA antigens
Fried et al 2018 Mali cohort T1/T2/T3 Multiplex ID1−ID2a 157 0.95 (0.49, 1.84)b
Fried et al 2018 Mali cohort T1/T2/T3 Multiplex DBL2 157 0.91 (0.41, 2.01)b
Fried et al 2018 Mali cohort T1/T2/T3 Multiplex DBL3 157 1.16 (0.54, 2.50)b
Fried et al 2018 Mali cohort T1/T2/T3 Multiplex DBL3-4 157 1.05 (0.49, 2.25)b
Fried et al 2018 Mali cohort T1/T2/T3 Multiplex DBL4 157 1.26 (0.34, 4.72)b
Fried et al 2018 Mali cohort T1/T2/T3 Multiplex DBL5 157 1.59 (0.42, 6.06)b
Guitard et al 2008 Senegal cohort T1/T2 ELISA DBL5 52 0.59 (0.23, 1.52)b
.2 .5 1 2 5

Supplementary Figure 4. Forest plot of the association between antibodies to pregnancy-associated *P. falciparum* antigens and placental malaria in primigravidae. A. Estimates represent the odds of placental malaria in Ab responders compared to Ab non-responders, where antibodies were measured at delivery (cross-sectional studies). B. Estimates represent the risk of placental malaria in Ab responders compared to non-responders, where antibodies were measured at time-points prior to delivery, as indicated (prospective studies). Estimates for McLean et al 2017 represent IgG3 responses, as total IgG was not available. Meta-analysis was only performed on estimates where VAR2CSA antigen or functional assay (where applicable) and timing of antibody determination were the same. [a]Estimate calculated by current authors from data in original publication; [b]Data supplied by original authors and estimate calculated by current authors. CC, case control; CS, cross-sectional; CSA adhes. inhib., CSA adhesion inhibition assay; n, number of participants included in estimate; OR, odds ratio; plac. isolate, placental isolate; pRBC, parasitized red blood cells; RR, risk ratio; T1, first trimester; T2, second trimester; T3, third trimester.

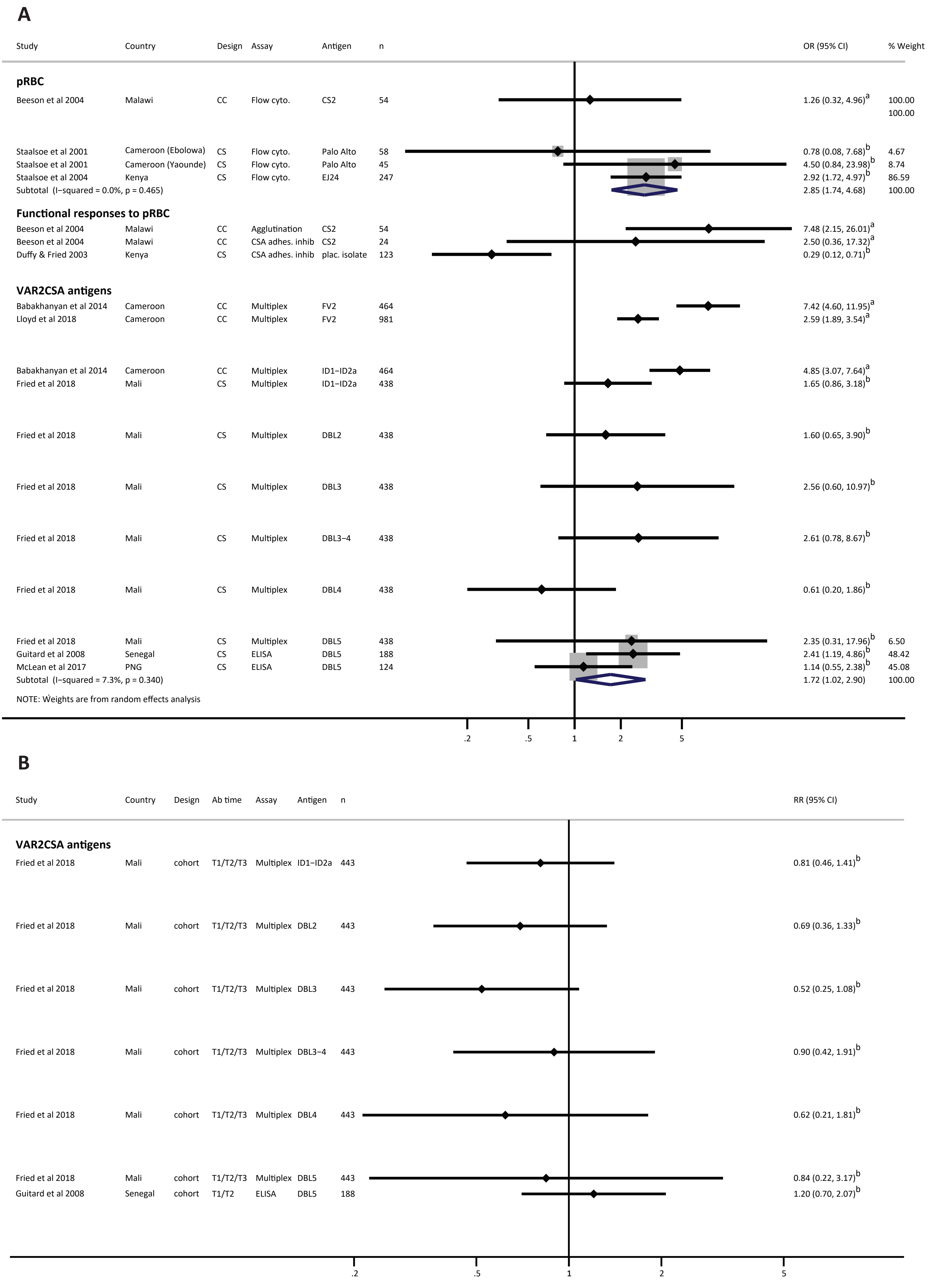
A
Study Country Design Assay Antigen n OR (95% CI) % Weight
pRBC
Beeson et al 2004 Malawi CC Flow cyto. CS2 54 1.26 (0.32, 4.96)[a] 100.00
100.00
Staalsoe et al 2001 Cameroon (Ebolowa) CS Flow cyto. Palo Alto 58 0.78 (0.08, 7.68)[b] 4.67
Staalsoe et al 2001 Cameroon (Yaounde) CS Flow cyto. Palo Alto 45 4.50 (0.84, 23.98)[b] 8.74
Staalsoe et al 2004 Kenya CS Flow cyto. EJ24 247 2.92 (1.72, 4.97)[b] 86.59
Subtotal (I−squared = 0.0%, p = 0.465) 2.85 (1.74, 4.68) 100.00
Functional responses to pRBC
Beeson et al 2004 Malawi CC Agglutination CS2 54 7.48 (2.15, 26.01)[a]
Beeson et al 2004 Malawi CC CSA adhes. inhib CS2 24 2.50 (0.36, 17.32)[a]
Duffy & Fried 2003 Kenya CS CSA adhes. inhib plac. isolate 123 0.29 (0.12, 0.71)[b]
VAR2CSA antigens
Babakhanyan et al 2014 Cameroon CC Multiplex FV2 464 7.42 (4.60, 11.95)[a]
Lloyd et al 2018 Cameroon CC Multiplex FV2 981 2.59 (1.89, 3.54)[a]
Babakhanyan et al 2014 Cameroon CC Multiplex ID1−ID2a 464 4.85 (3.07, 7.64)[a]
Fried et al 2018 Mali CS Multiplex ID1−ID2a 438 1.65 (0.86, 3.18)[b]
Fried et al 2018 Mali CS Multiplex DBL2 438 1.60 (0.65, 3.90)[b]
Fried et al 2018 Mali CS Multiplex DBL3 438 2.56 (0.60, 10.97)[b]
Fried et al 2018 Mali CS Multiplex DBL3−4 438 2.61 (0.78, 8.67)[b]
Fried et al 2018 Mali CS Multiplex DBL4 438 0.61 (0.20, 1.86)[b]
Fried et al 2018 Mali CS Multiplex DBL5 438 2.35 (0.31, 17.96)[b] 6.50
Guitard et al 2008 Senegal CS ELISA DBL5 188 2.41 (1.19, 4.86)[b] 48.42
McLean et al 2017 PNG CS ELISA DBL5 124 1.14 (0.55, 2.38)[b] 45.08
Subtotal (I−squared = 7.3%, p = 0.340) 1.72 (1.02, 2.90) 100.00
NOTE: Weights are from random effects analysis
.2 .5 1 2 5
B
Study Country Design Ab time Assay Antigen n RR (95% CI)
VAR2CSA antigens
Fried et al 2018 Mali cohort T1/T2/T3 Multiplex ID1−ID2a 443 0.81 (0.46, 1.41)[b]
Fried et al 2018 Mali cohort T1/T2/T3 Multiplex DBL2 443 0.69 (0.36, 1.33)[b]
Fried et al 2018 Mali cohort T1/T2/T3 Multiplex DBL3 443 0.52 (0.25, 1.08)[b]
Fried et al 2018 Mali cohort T1/T2/T3 Multiplex DBL3−4 443 0.90 (0.42, 1.91)[b]
Fried et al 2018 Mali cohort T1/T2/T3 Multiplex DBL4 443 0.62 (0.21, 1.81)[b]
Fried et al 2018 Mali cohort T1/T2/T3 Multiplex DBL5 443 0.84 (0.22, 3.17)[b]
Guitard et al 2008 Senegal cohort T1/T2 ELISA DBL5 188 1.20 (0.70, 2.07)[b]
.2 .5 1 2 5

Supplementary Figure 5. Forest plot of the association between antibodies to pregnancy-associated *P. falciparum* antigens and placental malaria in secundigravidae and multigravidae. A. Estimates represent the odds of placental malaria in Ab responders compared to Ab non-responders, where antibodies were measured at delivery (cross-sectional studies). B. Estimates represent the risk of placental malaria in Ab responders compared to non-responders, where antibodies were measured at time-points prior to delivery, as indicated (prospective studies). Estimates for McLean et al 2017 represent IgG3 responses, as total IgG was not available. Meta-analysis was only performed on estimates where VAR2CSA antigen or functional assay (where applicable) and timing of antibody determination were the same. [a]Estimate calculated by current authors from data in original publication; [b]Data supplied by original authors and estimate calculated by current authors. CC, case control; CS, cross-sectional; CSA adhes. inhib., CSA adhesion inhibition assay; n, number of participants included in estimate; OR, odds ratio; plac. isolate, placental isolate; pRBC, parasitized red blood cells; RR, risk ratio; T1, first trimester; T2, second trimester; T3, third trimester.

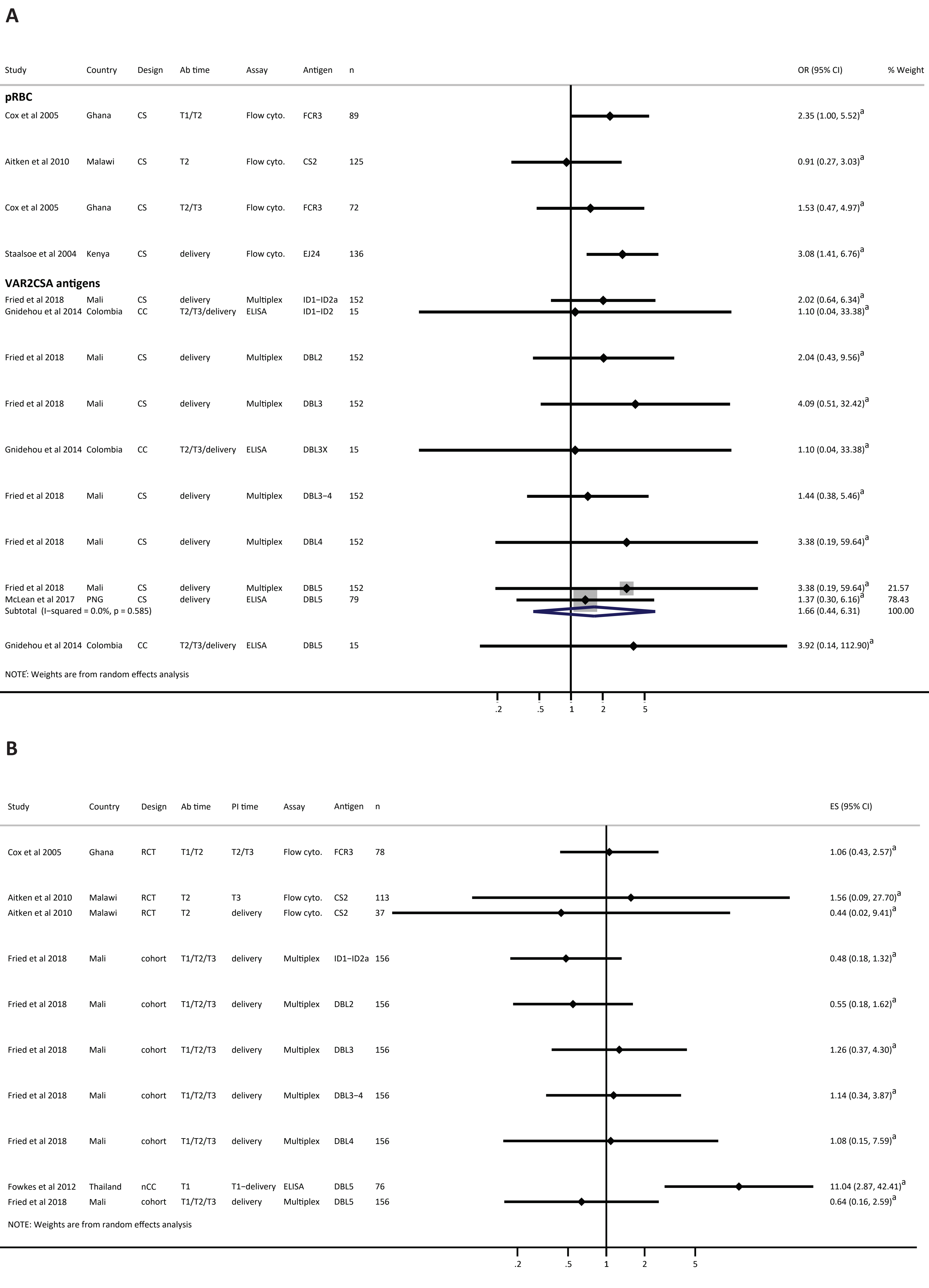
A
Study Country Design Ab time Assay Antigen n OR (95% CI) % Weight
pRBC
Cox et al 2005 Ghana CS T1/T2 Flow cyto. FCR3 89 2.35 (1.00, 5.52)[a]
Aitken et al 2010 Malawi CS T2 Flow cyto. CS2 125 0.91 (0.27, 3.03)[a]
Cox et al 2005 Ghana CS T2/T3 Flow cyto. FCR3 72 1.53 (0.47, 4.97)[a]
Staalsoe et al 2004 Kenya CS delivery Flow cyto. EJ24 136 3.08 (1.41, 6.76)[a]
VAR2CSA antigens
Fried et al 2018 Mali CS delivery Multiplex ID1−ID2a 152 2.02 (0.64, 6.34)[a]
Gnidehou et al 2014 Colombia CC T2/T3/delivery ELISA ID1−ID2 15 1.10 (0.04, 33.38)[a]
Fried et al 2018 Mali CS delivery Multiplex DBL2 152 2.04 (0.43, 9.56)[a]
Fried et al 2018 Mali CS delivery Multiplex DBL3 152 4.09 (0.51, 32.42)[a]
Gnidehou et al 2014 Colombia CC T2/T3/delivery ELISA DBL3X 15 1.10 (0.04, 33.38)[a]
Fried et al 2018 Mali CS delivery Multiplex DBL3−4 152 1.44 (0.38, 5.46)[a]
Fried et al 2018 Mali CS delivery Multiplex DBL4 152 3.38 (0.19, 59.64)[a]
Fried et al 2018 Mali CS delivery Multiplex DBL5 152 3.38 (0.19, 59.64)[a] 21.57
McLean et al 2017 PNG CS delivery ELISA DBL5 79 1.37 (0.30, 6.16)[a] 78.43
Subtotal (I−squared = 0.0%, p = 0.585) 1.66 (0.44, 6.31) 100.00
Gnidehou et al 2014 Colombia CC T2/T3/delivery ELISA DBL5 15 3.92 (0.14, 112.90)[a]
NOTE: Weights are from random effects analysis
.2 .5 1 2 5
B
Study Country Design Ab time PI time Assay Antigen n ES (95% CI)
Cox et al 2005 Ghana RCT T1/T2 T2/T3 Flow cyto. FCR3 78 1.06 (0.43, 2.57)[a]
Aitken et al 2010 Malawi RCT T2 T3 Flow cyto. CS2 113 1.56 (0.09, 27.70)[a]
Aitken et al 2010 Malawi RCT T2 delivery Flow cyto. CS2 37 0.44 (0.02, 9.41)[a]
Fried et al 2018 Mali cohort T1/T2/T3 delivery Multiplex ID1−ID2a 156 0.48 (0.18, 1.32)[a]
Fried et al 2018 Mali cohort T1/T2/T3 delivery Multiplex DBL2 156 0.55 (0.18, 1.62)[a]
Fried et al 2018 Mali cohort T1/T2/T3 delivery Multiplex DBL3 156 1.26 (0.37, 4.30)[a]
Fried et al 2018 Mali cohort T1/T2/T3 delivery Multiplex DBL3−4 156 1.14 (0.34, 3.87)[a]
Fried et al 2018 Mali cohort T1/T2/T3 delivery Multiplex DBL4 156 1.08 (0.15, 7.59)[a]
Fowkes et al 2012 Thailand nCC T1 T1−delivery ELISA DBL5 76 11.04 (2.87, 42.41)[a]
Fried et al 2018 Mali cohort T1/T2/T3 delivery Multiplex DBL5 156 0.64 (0.16, 2.59)[a]
NOTE: Weights are from random effects analysis
.2 .5 1 2 5

Supplementary Figure 6. Forest plot of the association between antibodies to pregnancy-associated *P. falciparum* antigens and peripheral parasitaemia in primigravidae. A. Estimates represent the odds of peripheral P. falciparum parasitaemia in Ab responders compared to Ab non-responders, where antibodies were measured at the same time point as parasitaemia (cross-sectional studies). B. Estimates of peripheral *P. falciparum* parasitaemia in Ab responders compared to Ab non-responders, were antibodies were measured at time-points prior to parasitaemia determination. Estimates for RCT and cohort studies are risk ratios and estimate for the nested case control study is an odds ratio. The timing of antibody and parasitaemia determination is indicated. Estimate for McLean et al 2017 represents IgG3 responders only as total IgG was not available. DBL5 estimates were only combined when study design was the same. [a]Data supplied by original authors and estimate calculated by current authors. CS, cross-sectional; ES, estimate; n, number of participants included in estimate; Flow cyto., flow cytometry; nCC, nested case control; OR, odds ratio; PI time, timing of determination of parasitaemia; pRBC, parasitized red blood cells; RCT, randomized controlled trial; T1, first trimester; T2, second trimester; T3, third trimester.

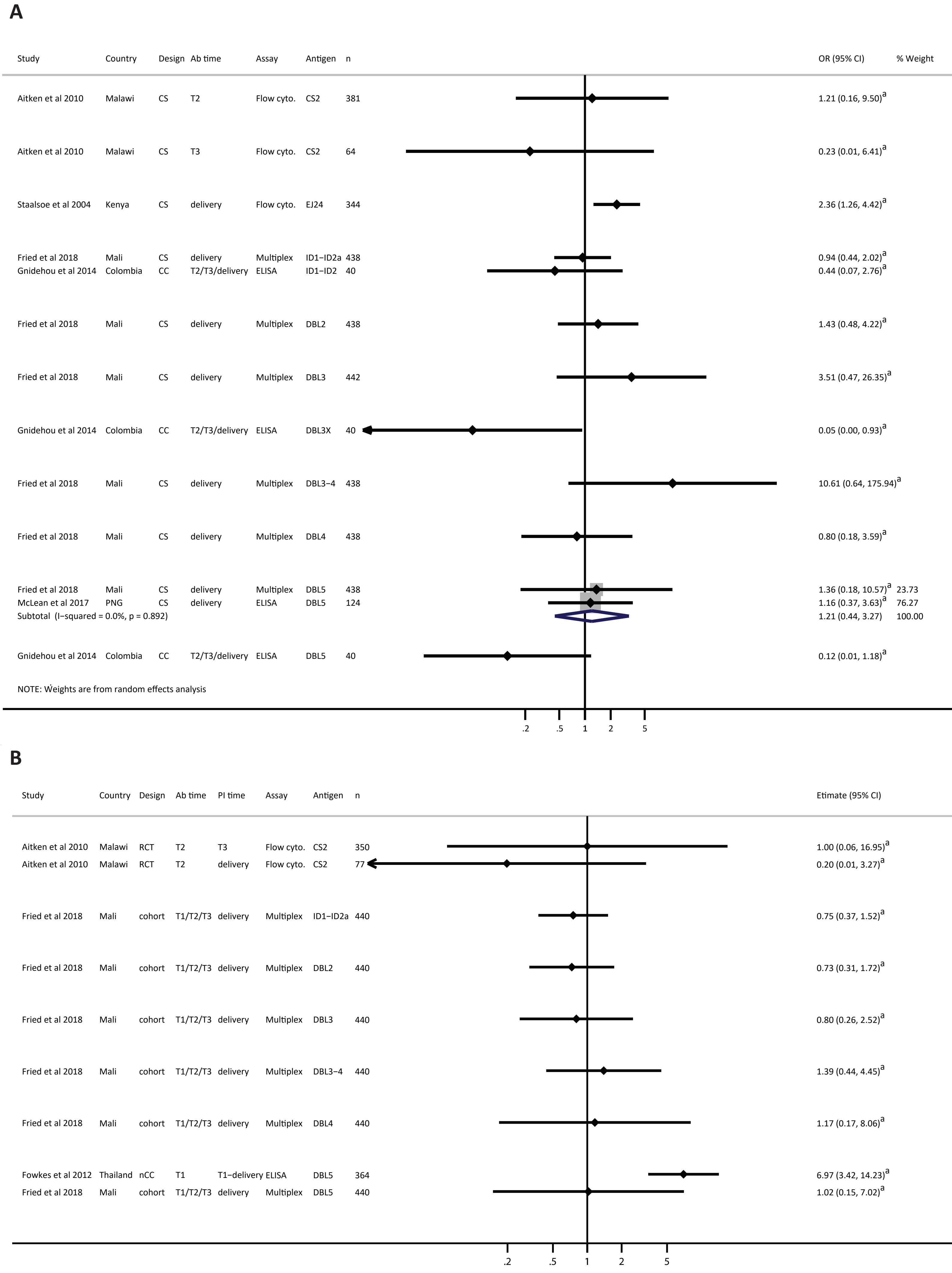
A
Study Country Design Ab time Assay Antigen n
OR (95% CI) % Weight
Aitken et al 2010 Malawi CS T2 Flow cyto. CS2 381 1.21 (0.16, 9.50)a
Aitken et al 2010 Malawi CS T3 Flow cyto. CS2 64 0.23 (0.01, 6.41)a
Staalsoe et al 2004 Kenya CS delivery Flow cyto. EJ24 344 2.36 (1.26, 4.42)a
Fried et al 2018 Mali CS delivery Multiplex ID1−ID2a 438 0.94 (0.44, 2.02)a
Gnidehou et al 2014 Colombia CC T2/T3/delivery ELISA ID1−ID2 40 0.44 (0.07, 2.76)a
Fried et al 2018 Mali CS delivery Multiplex DBL2 438 1.43 (0.48, 4.22)a
Fried et al 2018 Mali CS delivery Multiplex DBL3 442 3.51 (0.47, 26.35)a
Gnidehou et al 2014 Colombia CC T2/T3/delivery ELISA DBL3X 40 0.05 (0.00, 0.93)a
Fried et al 2018 Mali CS delivery Multiplex DBL3−4 438 10.61 (0.64, 175.94)a
Fried et al 2018 Mali CS delivery Multiplex DBL4 438 0.80 (0.18, 3.59)a
Fried et al 2018 Mali CS delivery Multiplex DBL5 438 1.36 (0.18, 10.57)a 23.73
McLean et al 2017 PNG CS delivery ELISA DBL5 124 1.16 (0.37, 3.63)a 76.27
Subtotal (I−squared = 0.0%, p = 0.892) 1.21 (0.44, 3.27) 100.00
Gnidehou et al 2014 Colombia CC T2/T3/delivery ELISA DBL5 40 0.12 (0.01, 1.18)a
NOTE: Weights are from random effects analysis
.2 .5 1 2 5
B
Study Country Design Ab time PI time Assay Antigen n
Etimate (95% CI)
Aitken et al 2010 Malawi RCT T2 T3 Flow cyto. CS2 350 1.00 (0.06, 16.95)a
Aitken et al 2010 Malawi RCT T2 delivery Flow cyto. CS2 77 0.20 (0.01, 3.27)a
Fried et al 2018 Mali cohort T1/T2/T3 delivery Multiplex ID1−ID2a 440 0.75 (0.37, 1.52)a
Fried et al 2018 Mali cohort T1/T2/T3 delivery Multiplex DBL2 440 0.73 (0.31, 1.72)a
Fried et al 2018 Mali cohort T1/T2/T3 delivery Multiplex DBL3 440 0.80 (0.26, 2.52)a
Fried et al 2018 Mali cohort T1/T2/T3 delivery Multiplex DBL3−4 440 1.39 (0.44, 4.45)a
Fried et al 2018 Mali cohort T1/T2/T3 delivery Multiplex DBL4 440 1.17 (0.17, 8.06)a
Fowkes et al 2012 Thailand nCC T1 T1−delivery ELISA DBL5 364 6.97 (3.42, 14.23)a
Fried et al 2018 Mali cohort T1/T2/T3 delivery Multiplex DBL5 440 1.02 (0.15, 7.02)a
.2 .5 1 2 5

Supplementary Figure 7. Forest plot of the association between antibodies to pregnancy-associated *P. falciparum* antigens and peripheral parasitaemia in secundigravidae and multigravidae. A. Estimates represent the odds of peripheral *P. falciparum* parasitaemia in Ab responders compared to Ab non-responders, where antibodies were measured at the same time point as parasitaemia (cross-sectional studies). B. Estimates of peripheral *P. falciparum* parasitaemia in Ab responders compared to Ab non-responders, were antibodies were measured at time-points prior to parasitaemia determination. Estimates for RCT and cohort studies are risk ratios and estimate for the nested case control study is an odds ratio. The timing of antibody and parasitaemia determination is indicated. Estimate for McLean et al 2017 represents IgG3 responders only as total IgG was not available. DBL5 estimates were only combined when study design was the same. [a]Data supplied by original authors and estimate calculated by current authors. CC, case control; CS, cross-sectional; ES, estimate; n, number of participants included in estimate; Flow cyto., flow cytometry; nCC, nested case control; OR, odds ratio; PI time, timing of determination of parasitaemia; pRBC, parasitized red blood cells; RCT, randomized controlled trial; T1, first trimester; T2, second trimester; T3, third trimester.

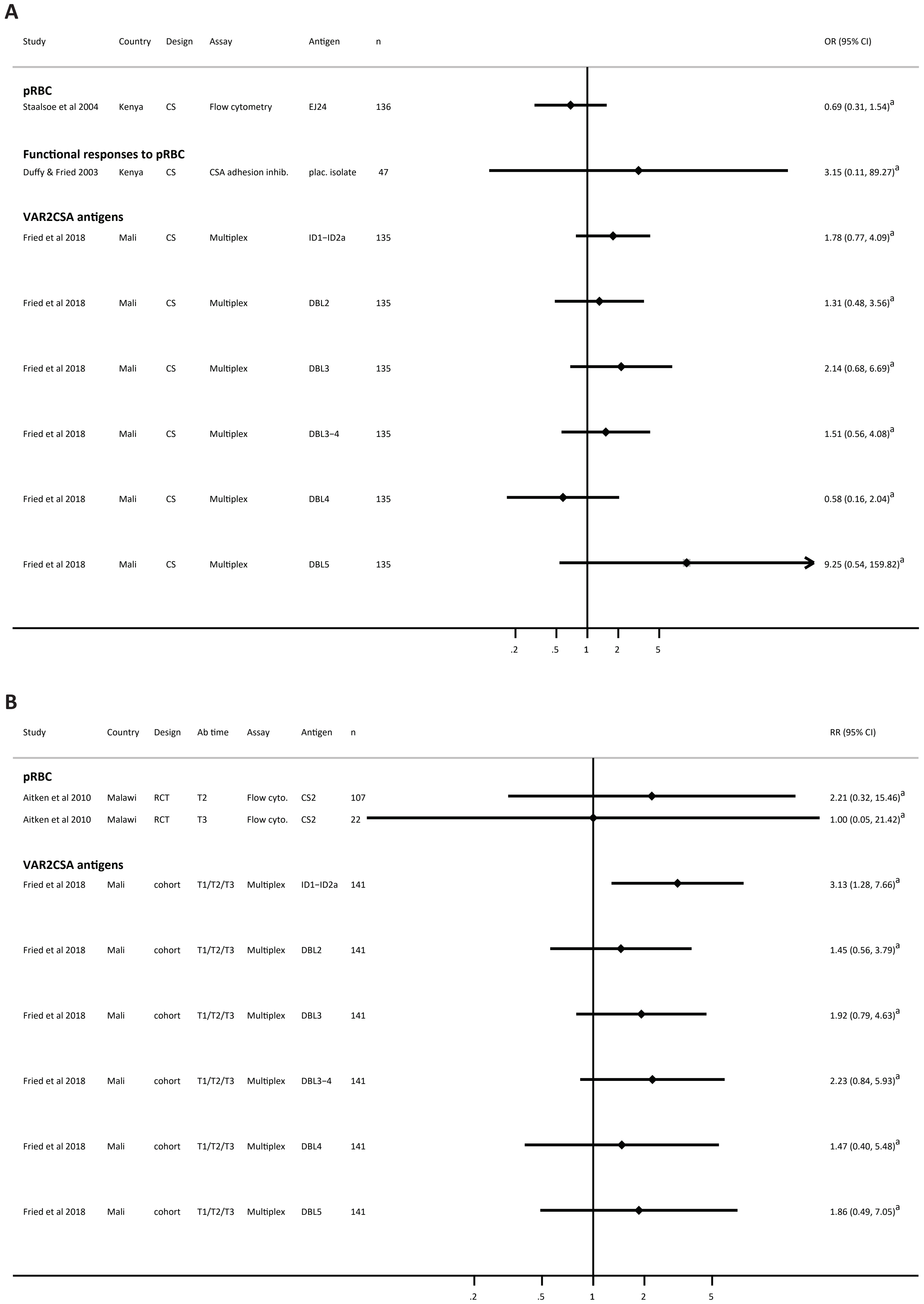

A
Study
Country
Design
Assay
Antigen
n
OR (95% CI)
pRBC
Staalsoe et al 2004 Kenya CS Flow cytometry EJ24 136 0.69 (0.31, 1.54)[a]
Functional responses to pRBC
Duffy & Fried 2003 Kenya CS CSA adhesion inhib. plac. isolate 47 3.15 (0.11, 89.27)[a]
VAR2CSA antigens
Fried et al 2018 Mali CS Multiplex ID1−ID2a 135 1.78 (0.77, 4.09)[a]
Fried et al 2018 Mali CS Multiplex DBL2 135 1.31 (0.48, 3.56)[a]
Fried et al 2018 Mali CS Multiplex DBL3 135 2.14 (0.68, 6.69)[a]
Fried et al 2018 Mali CS Multiplex DBL3−4 135 1.51 (0.56, 4.08)[a]
Fried et al 2018 Mali CS Multiplex DBL4 135 0.58 (0.16, 2.04)[a]
Fried et al 2018 Mali CS Multiplex DBL5 135 9.25 (0.54, 159.82)[a]
.2
.5
1
2
5
B
Study
Country
Design
Ab time
Assay
Antigen
n
RR (95% CI)
pRBC
Aitken et al 2010 Malawi RCT T2 Flow cyto. CS2 107 2.21 (0.32, 15.46)[a]
Aitken et al 2010 Malawi RCT T3 Flow cyto. CS2 22 1.00 (0.05, 21.42)[a]
VAR2CSA antigens
Fried et al 2018 Mali cohort T1/T2/T3 Multiplex ID1−ID2a 141 3.13 (1.28, 7.66)[a]
Fried et al 2018 Mali cohort T1/T2/T3 Multiplex DBL2 141 1.45 (0.56, 3.79)[a]
Fried et al 2018 Mali cohort T1/T2/T3 Multiplex DBL3 141 1.92 (0.79, 4.63)[a]
Fried et al 2018 Mali cohort T1/T2/T3 Multiplex DBL3−4 141 2.23 (0.84, 5.93)[a]
Fried et al 2018 Mali cohort T1/T2/T3 Multiplex DBL4 141 1.47 (0.40, 5.48)[a]
Fried et al 2018 Mali cohort T1/T2/T3 Multiplex DBL5 141 1.86 (0.49, 7.05)[a]
.2
.5
1
2
5

Supplementary Figure 8. Forest plot of the association between antibodies to pregnancy-associated *P. falciparum* antigens and low birthweight in primigravidae. A. Estimates represent the odds of low birthweight birth in Ab responders compared with Ab non-responders, where antibodies were measured at delivery (cross-sectional studies). B. Estimates represent the risk of low birthweight birth in Ab responders compared with Ab non-responders, where antibodies were measured at time-points prior to delivery, as indicated (prospective studies). [a]Data supplied by original authors and estimate calculated by current authors. CC, case-control; CS, cross-sectional; n, number of participants included in estimate; OR, odds ratio; pRBC, parasitized red blood cells; RCT, randomized controlled trial; RR, risk ratio; T1, first trimester; T2, second trimester; T3, third trimester.

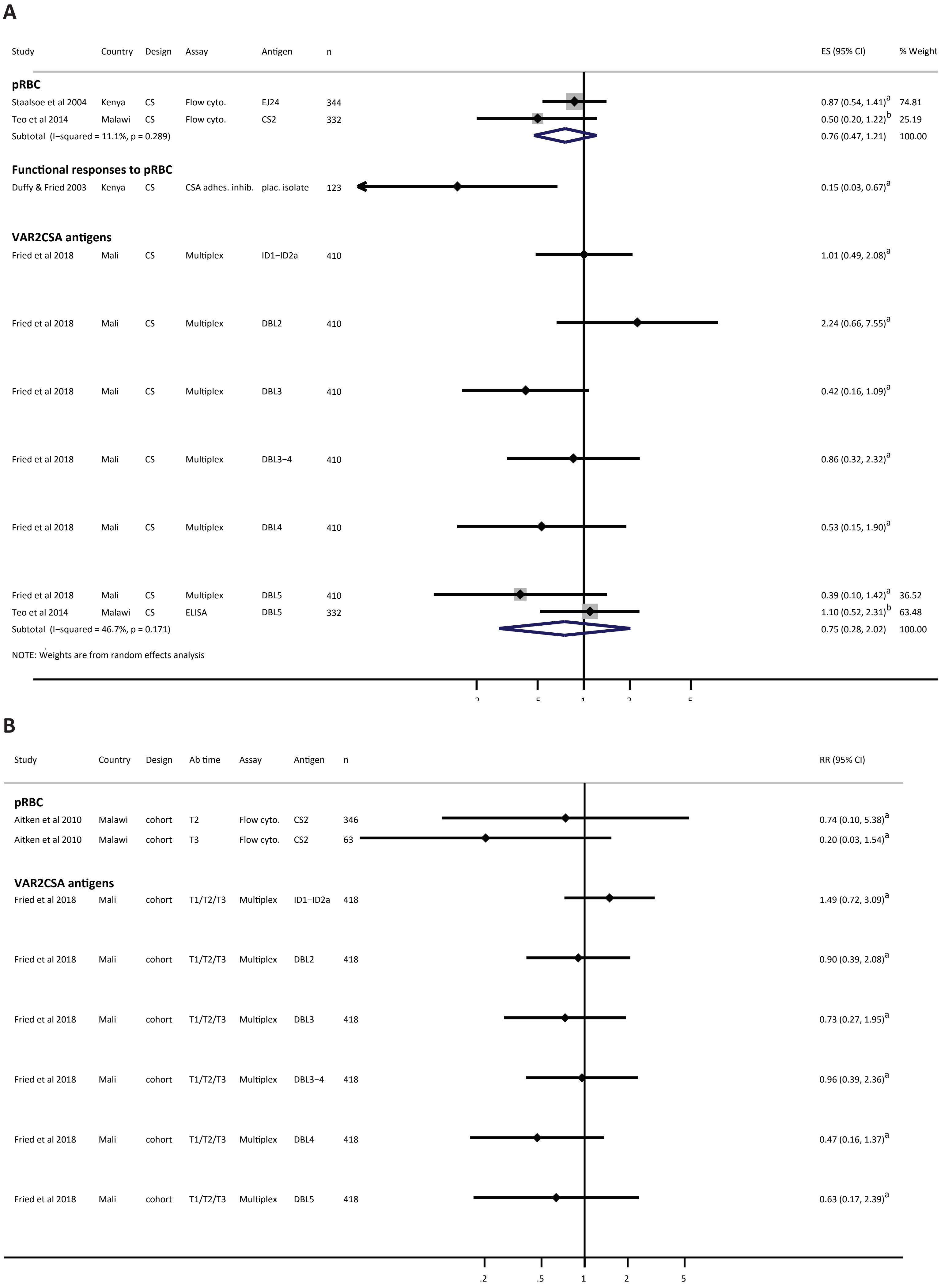
A
Study Country Design Assay Antigen n ES (95% CI) % Weight
pRBC
Staalsoe et al 2004 Kenya CS Flow cyto. EJ24 344 0.87 (0.54, 1.41)a 74.81
Teo et al 2014 Malawi CS Flow cyto. CS2 332 0.50 (0.20, 1.22)b 25.19
Subtotal (I−squared = 11.1%, p = 0.289) 0.76 (0.47, 1.21) 100.00
Functional responses to pRBC
Duffy & Fried 2003 Kenya CS CSA adhes. inhib. plac. isolate 123 0.15 (0.03, 0.67)a
VAR2CSA antigens
Fried et al 2018 Mali CS Multiplex ID1−ID2a 410 1.01 (0.49, 2.08)a
Fried et al 2018 Mali CS Multiplex DBL2 410 2.24 (0.66, 7.55)a
Fried et al 2018 Mali CS Multiplex DBL3 410 0.42 (0.16, 1.09)a
Fried et al 2018 Mali CS Multiplex DBL3−4 410 0.86 (0.32, 2.32)a
Fried et al 2018 Mali CS Multiplex DBL4 410 0.53 (0.15, 1.90)a
Fried et al 2018 Mali CS Multiplex DBL5 410 0.39 (0.10, 1.42)a 36.52
Teo et al 2014 Malawi CS ELISA DBL5 332 1.10 (0.52, 2.31)b 63.48
Subtotal (I−squared = 46.7%, p = 0.171) 0.75 (0.28, 2.02) 100.00
NOTE: Weights are from random effects analysis
B
Study Country Design Ab time Assay Antigen n RR (95% CI)
pRBC
Aitken et al 2010 Malawi cohort T2 Flow cyto. CS2 346 0.74 (0.10, 5.38)a
Aitken et al 2010 Malawi cohort T3 Flow cyto. CS2 63 0.20 (0.03, 1.54)a
VAR2CSA antigens
Fried et al 2018 Mali cohort T1/T2/T3 Multiplex ID1−ID2a 418 1.49 (0.72, 3.09)a
Fried et al 2018 Mali cohort T1/T2/T3 Multiplex DBL2 418 0.90 (0.39, 2.08)a
Fried et al 2018 Mali cohort T1/T2/T3 Multiplex DBL3 418 0.73 (0.27, 1.95)a
Fried et al 2018 Mali cohort T1/T2/T3 Multiplex DBL3−4 418 0.96 (0.39, 2.36)a
Fried et al 2018 Mali cohort T1/T2/T3 Multiplex DBL4 418 0.47 (0.16, 1.37)a
Fried et al 2018 Mali cohort T1/T2/T3 Multiplex DBL5 418 0.63 (0.17, 2.39)a
.2
.5
1
2
5

Supplementary Figure 9. Forest plot of the association between antibodies to pregnancy-associated *P. falciparum* antigens and low birthweight in secundigravidae and multigravidae. A. Estimates represent the odds of low birthweight birth in Ab responders compared with Ab non-responders, where antibodies were measured at delivery (cross-sectional studies). B. Estimates represent the risk of low birthweight birth in Ab responders compared with Ab non-responders, where antibodies were measured at time-points prior to delivery, as indicated (prospective studies). [a]Data supplied by original authors and estimate calculated by current authors. CC, case-control; CS, cross-sectional; ES, estimate; n, number of participants included in estimate; pRBC, parasitized red blood cells; RCT, randomized controlled trial; RR, risk ratio; T1, first trimester; T2, second trimester; T3, third trimester.

A

Study	Country	Design	Ab time	Test time	Assay	Antigen	Anaemia def.	n	ES (95% CI)
Aitken et al 2010	Malawi	CS	T2	T2	Flow cyto.	CS2	Hb <110 g/l	125	0.73 (0.26, 2.01)[a]
Aitken et al 2010	Malawi	RCT	T2	T3	Flow cyto.	CS2	Hb <110 g/l	116	0.76 (0.47, 1.24)[a]
Aitken et al 2010	Malawi	CS	T3	T3	Flow cyto.	CS2	Hb <110 g/l	23	12.18 (0.57, 257.97)[a]
Staalsoe et al 2004	Kenya	CS	D	D	Flow cyto.	EJ24	Hb <70 g/l	136	0.78 (0.28, 2.16)[a]
Duffy & Fried 2003	Kenya	CS	D	D	CSA adhes. inhib.	plac. isolate	Hb <110 g/l	47	0.11 (0.00, 2.84)[a]

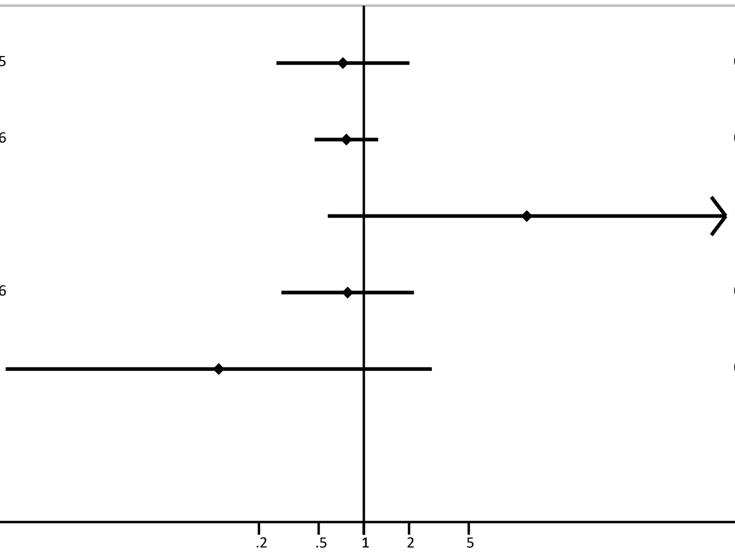

B

Study	Country	Design	Ab time	Test time	Assay	Antigen	Anaemia def.	n	ES (95% CI)
Aitken et al 2010	Malawi	CS	T2	T2	Flow cyto.	CS2	Hb <110 g/l	383	1.57 (0.64, 3.88)[a]
Aitken et al 2010	Malawi	RCT	T2	T3	Flow cyto.	CS2	Hb <110 g/l	356	4.45 (1.18, 16.73)[a]
Aitken et al 2010	Malawi	CS	T3	T3	Flow cyto.	CS2	Hb <110 g/l	62.5	0.44 (0.04, 4.74)[a]
Staalsoe et al 2004	Kenya	CS	D	D	Flow cyto.	EJ24	Hb <70g/l	344	1.19 (0.65, 2.20)[a]
Duffy & Fried 2003	Kenya	CS	D	D	CSA adhes. inhib.	plac. isolate	Hb <110 g/l	123	1.25 (0.44, 3.55)[a]

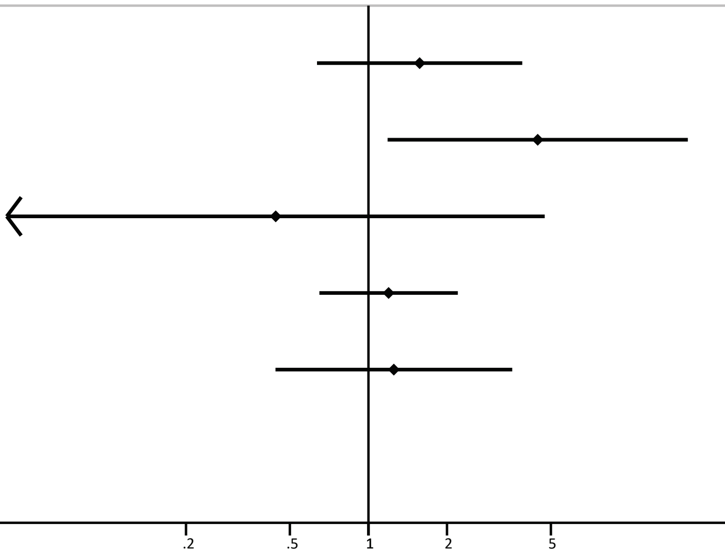

Supplementary Figure 10. Forest plot of the association between antibodies to pregnancy-associated *P. falciparum* antigens and anaemia in primigravidae (A) and secundigravidae and multigravidae (B). Estimates represent the odds (CS and CC studies) or risk (RCT) of anaemia or severe anaemia, as defined in individual publications, in Ab responders compared to Ab non-responders. Timing of antibody determination (Ab time) and anaemia determination (Test time) are as indicated. [a]Data supplied by original authors and estimate calculated by current authors; [b]Estimate calculated by current authors from data in original publication. CS, cross-sectional; CSA adhes. inhib., CSA adhesion inhibition assay; CC, case-control; ES, estimate; Flow cyto., flow cytometry; Hb, haemoglobin; n, number of participants included in estimate; plac. isolate, placental isolate; PCV, packed cell volume; RCT, randomized controlled trial; T2, second trimester; T3, third trimester.

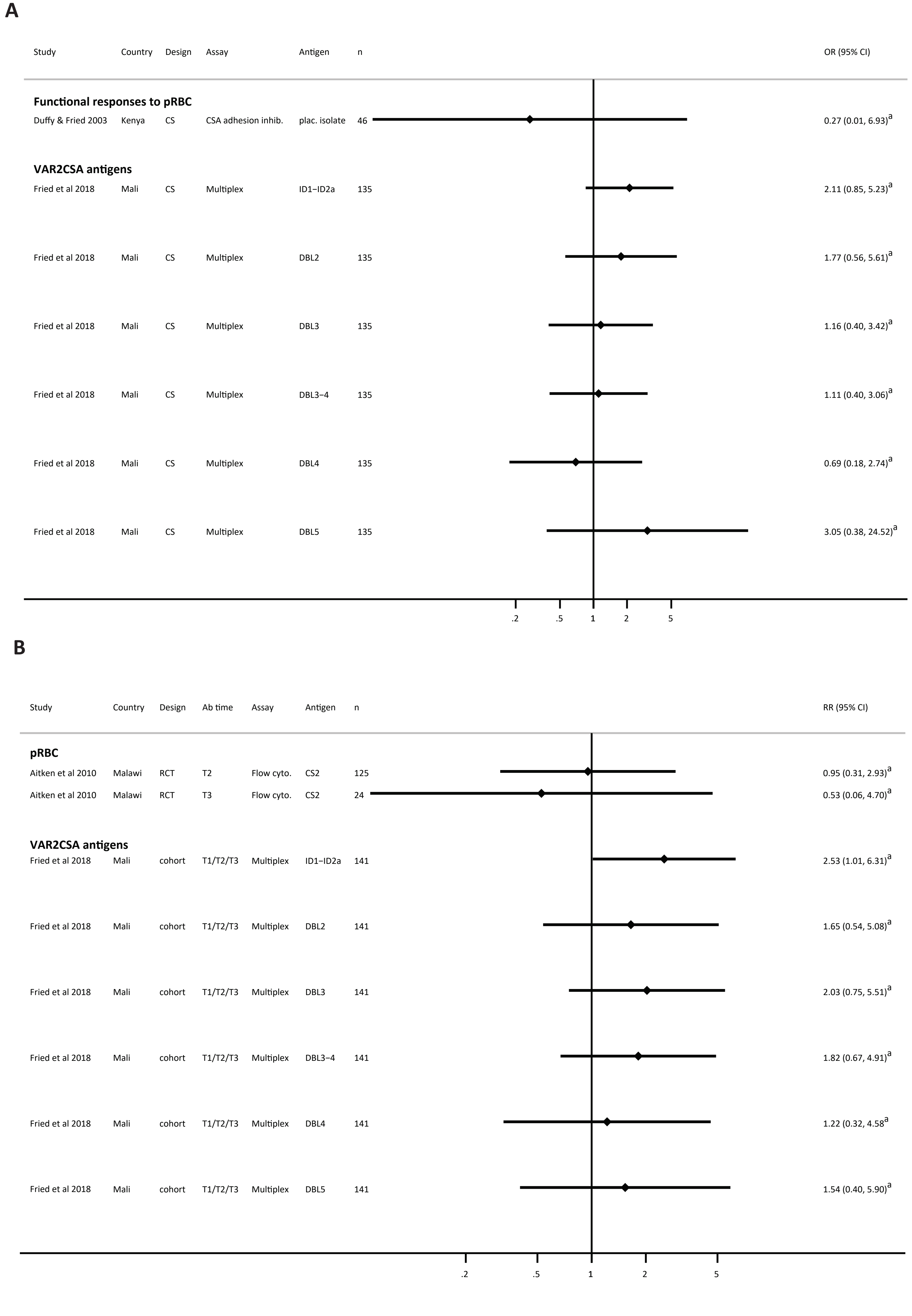
A
Study Country Design Assay Antigen n
OR (95% CI)
Functional responses to pRBC
Duffy & Fried 2003 Kenya CS CSA adhesion inhib. plac. isolate 46
0.27 (0.01, 6.93)a
VAR2CSA antigens
Fried et al 2018 Mali CS Multiplex ID1–ID2a 135
2.11 (0.85, 5.23)a
Fried et al 2018 Mali CS Multiplex DBL2 135
1.77 (0.56, 5.61)a
Fried et al 2018 Mali CS Multiplex DBL3 135
1.16 (0.40, 3.42)a
Fried et al 2018 Mali CS Multiplex DBL3–4 135
1.11 (0.40, 3.06)a
Fried et al 2018 Mali CS Multiplex DBL4 135
0.69 (0.18, 2.74)a
Fried et al 2018 Mali CS Multiplex DBL5 135
3.05 (0.38, 24.52)a
.2
.5
1
2
5
B
Study Country Design Ab time Assay Antigen n
RR (95% CI)
pRBC
Aitken et al 2010 Malawi RCT T2 Flow cyto. CS2 125
0.95 (0.31, 2.93)a
Aitken et al 2010 Malawi RCT T3 Flow cyto. CS2 24
0.53 (0.06, 4.70)a
VAR2CSA antigens
Fried et al 2018 Mali cohort T1/T2/T3 Multiplex ID1–ID2a 141
2.53 (1.01, 6.31)a
Fried et al 2018 Mali cohort T1/T2/T3 Multiplex DBL2 141
1.65 (0.54, 5.08)a
Fried et al 2018 Mali cohort T1/T2/T3 Multiplex DBL3 141
2.03 (0.75, 5.51)a
Fried et al 2018 Mali cohort T1/T2/T3 Multiplex DBL3–4 141
1.82 (0.67, 4.91)a
Fried et al 2018 Mali cohort T1/T2/T3 Multiplex DBL4 141
1.22 (0.32, 4.58a
Fried et al 2018 Mali cohort T1/T2/T3 Multiplex DBL5 141
1.54 (0.40, 5.90)a
.2
.5
1
2
5

Supplementary Figure 11. Forest plot of the association between antibodies to pregnancy-associated *P. falciparum* antigens and preterm birth in primigravidae. A. Estimates represent the odds of preterm birth in Ab responders compared to Ab non-responders, where antibodies were measured at delivery (cross-sectional studies). B. Estimates represent the risk of preterm birth in Ab responders compared to non-responders, where antibodies were measured at time-points prior to delivery, as indicated (prospective studies). Meta-analysis was not performed as antigens differed between studies [a]Data supplied by original authors and estimate calculated by current authors. CC, case control; CS, cross-sectional; CSA adhesion inhib., CSA adhesion inhibition assay; Flow cyto., flow cytometry; n, number of participants included in estimate; OR, odds ratio; plac. isolate, placental isolate; pRBC, parasitized red blood cells; RCT, randomized controlled trial; RR, risk ratio; T1, first trimester; T2, second trimester; T3, third trimester.

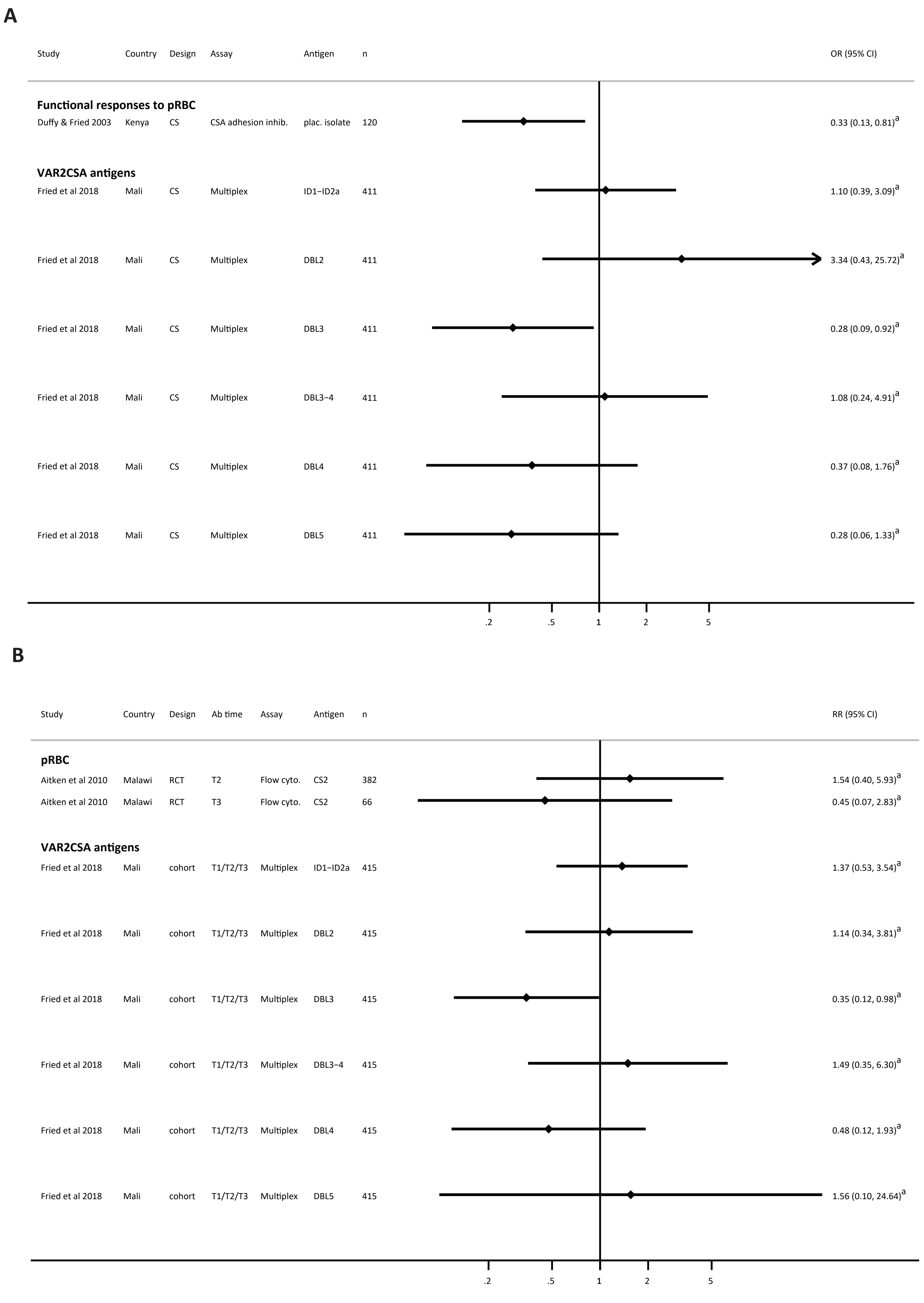
A
Study
Country
Design
Assay
Antigen
n
OR (95% CI)
Functional responses to pRBC
Duffy & Fried 2003 Kenya CS CSA adhesion inhib. plac. isolate 120 0.33 (0.13, 0.81)a
VAR2CSA antigens
Fried et al 2018 Mali CS Multiplex ID1–ID2a 411 1.10 (0.39, 3.09)a
Fried et al 2018 Mali CS Multiplex DBL2 411 3.34 (0.43, 25.72)a
Fried et al 2018 Mali CS Multiplex DBL3 411 0.28 (0.09, 0.92)a
Fried et al 2018 Mali CS Multiplex DBL3–4 411 1.08 (0.24, 4.91)a
Fried et al 2018 Mali CS Multiplex DBL4 411 0.37 (0.08, 1.76)a
Fried et al 2018 Mali CS Multiplex DBL5 411 0.28 (0.06, 1.33)a
.2
.5
1
2
5
B
Study
Country
Design
Ab time
Assay
Antigen
n
RR (95% CI)
pRBC
Aitken et al 2010 Malawi RCT T2 Flow cyto. CS2 382 1.54 (0.40, 5.93)a
Aitken et al 2010 Malawi RCT T3 Flow cyto. CS2 66 0.45 (0.07, 2.83)a
VAR2CSA antigens
Fried et al 2018 Mali cohort T1/T2/T3 Multiplex ID1–ID2a 415 1.37 (0.53, 3.54)a
Fried et al 2018 Mali cohort T1/T2/T3 Multiplex DBL2 415 1.14 (0.34, 3.81)a
Fried et al 2018 Mali cohort T1/T2/T3 Multiplex DBL3 415 0.35 (0.12, 0.98)a
Fried et al 2018 Mali cohort T1/T2/T3 Multiplex DBL3–4 415 1.49 (0.35, 6.30)a
Fried et al 2018 Mali cohort T1/T2/T3 Multiplex DBL4 415 0.48 (0.12, 1.93)a
Fried et al 2018 Mali cohort T1/T2/T3 Multiplex DBL5 415 1.56 (0.10, 24.64)a
.2
.5
1
2
5

Supplementary Figure 12. Forest plot of the association between antibodies to pregnancy-associated *P. falciparum* antigens and preterm birth in secundigravidae and multigravidae. A. Estimates represent the odds of preterm birth in Ab responders compared to Ab non-responders, where antibodies were measured at delivery (cross-sectional studies). B. Estimates represent the risk of preterm birth in Ab responders compared to non-responders, where antibodies were measured at time-points prior to delivery, as indicated (prospective studies). Meta-analysis was not performed as antigens differed between studies [a]Data supplied by original authors and estimate calculated by current authors. CC, case control; CS, cross-sectional; CSA adhesion inhib., CSA adhesion inhibition assay; Flow cyto., flow cytometry; n, number of participants included in estimate; OR, odds ratio; plac. isolate, placental isolate; pRBC, parasitized red blood cells; RCT, randomized controlled trial; RR, risk ratio; T1, first trimester; T2, second trimester; T3, third trimester.
